# Post-transcriptional modification of m^6^A methylase METTL3 regulates ERK-induced androgen-deprived treatment resistance prostate cancer

**DOI:** 10.1038/s41419-023-05773-5

**Published:** 2023-04-24

**Authors:** Yang Li, Shimiao Zhu, Yutong Chen, Qianwang Ma, Duo Kan, Wenyue Yu, Boya Zhang, Xuanrong Chen, Wanqing Wei, Yi Shao, Keruo Wang, Mingpeng Zhang, Shu Deng, Yuanjie Niu, Zhiqun Shang

**Affiliations:** 1 Tianjin Institute of Urology, The Second Hospital of Tianjin Medical University, Tianjin, China; 2 Lianshui People’s Hospital of Kangda College affiliated with Nanjing Medical University, Huai’an, China

**Keywords:** Prostate cancer, Drug development, Antisense oligonucleotide therapy

## Abstract

As the most common modification of RNA, N^6^-methyladenosin (m^6^A) has been confirmed to be involved in the occurrence and development of various cancers. However, the relationship between m^6^A and castration resistance prostate cancer (CRPC), has not been fully studied. By m^6^A-sequencing of patient cancer tissues, we identified that the overall level of m^6^A in CRPC was up-regulated than castration sensitive prostate cancer (CSPC). Based on the analysis of m^6^A-sequencing data, we found m^6^A modification level of HRas proto-oncogene, GTPase (HRAS) and mitogen-activated protein kinase kinase 2 (MEK2 or MAP2K2) were enhanced in CRPC. Specifically, tissue microarray analysis and molecular biology experiments confirmed that METTL3, an m^6^A “writer” up-regulated after castration, activated the ERK pathway to contribute to malignant phenotype including ADT resistance, cell proliferation and invasion. We revealed that METTL3-mediated ERK phosphorylation by stabilizing the transcription of HRAS and positively regulating the translation of MEK2. In the Enzalutamide-resistant (Enz-R) C4-2 and LNCap cell line (C4-2^R^, LNCap^R^) established in the current study, the ERK pathway was confirmed to be regulated by METTL3. We also found that applying antisense oligonucleotides (ASOs) to target the METTL3/ERK axis can restore Enzalutamide resistance in vitro and in vivo. In conclusion, METTL3 activated the ERK pathway and induced the resistance to Enzalutamide by regulating the m^6^A level of critical gene transcription in the ERK pathway.

## Introduction

Prostate cancer is a genitourinary malignancy that challenges significantly the male population [1]. In North America, PCa has surpassed lung cancer possessing the highest cumulative incidence among all the malignant tumors in men [2]. Although the androgen receptor (AR) is the most important treatment target for prostate cancer, with the widespread use of Enzalutamide, an increasing number of patients receiving androgen deprivation treatment (ADT) have progressed to the CRPC stage [3–5]. Therefore, developing new therapeutic targets in the CRPC stage has been one of the most widely studied targets for contemporary medical research [6].

Even though m^6^A has been discovered for more than 40 years, the mechanism of its biological function has been gradually uncovered in the recent years. As we all know, m^6^A is regulated by the methyltransferase complex (“writer”), demethylase (“eraser”) and RNA binding protein (“reader”) [7]. Methyltransferase-like 3 (METTL3), methyltransferase 14 (METTL14) and Wilms’ tumor 1 binding protein (WTAP) form a core methyltransferase complex [7]. The two demethylases are Fat mass/obesity-related protein (FTO) and AlkB homolog 5 (ALKBH5) [7]. m^6^A can regulate the alternative splicing, histone methylation [8, 9], translation efficiency [10] and decay rate of coding or non-coding RNA [11, 12] affecting various biological processes, such as cell differentiation, cell metabolism [13], cell senescence [14], and stress response [15]. In cancer-associated biological processes, METTL3, the most important subunit of the methyltransferase complex, has been reported to promote the hypoxic tolerance [16], drug resistance [17, 18], immune response [19] and EMT [20] in multiple cancer cells.

The ERK signaling pathway is one of the most common overactivated signaling pathways in cancer [21]. In some CRPC patients, especially who received ADT treatment, the AR signaling pathway gradually loses its dominant position and is supplanted by some pathways such as ERK or AKT signaling pathways to drive the progression of prostate cancer [22–24]. Combining preclinical and clinical studies of CRPC, we found that the RAS/RAF/MEK/ERK axis not only enhances the malignancy of tumors, but also generates early recurrence and reduces disease-specific survival rates [25]. Furthermore, the ERK pathway could interestingly enhance the transdifferentiation of CRPC to neuroendocrine prostate cancer (NEPC) to a certain extent [26].

Here, we demonstrate that ERK pathway was activated by METTL3 in CRPC. Our group first confirmed the up-regulation of m^6^A in CRPC than CSPC. Then, our results revealed the increased level of METTL3 protein in CRPC vs CSPC, and found its positively correlation with ERK pathway genes. In molecular mechanism-related studies, we have confirmed that METTL3 regulates the transcriptional stability of HRAS gene and the protein quantity of MEK2 gene through m^6^A. Finally, METTL3 was upregulated and the ERK pathway was activated in drug-resistant cells. In vitro and in vivo experiments have confirmed that ASO targeting METTL3 can reduce the proliferation of drug-resistant prostate cancer when combined with Enzalutamine.

## Results

### MeRIP-seq of CRPC tumours with enhanced m^6^A methylation

**Fig. 1:**
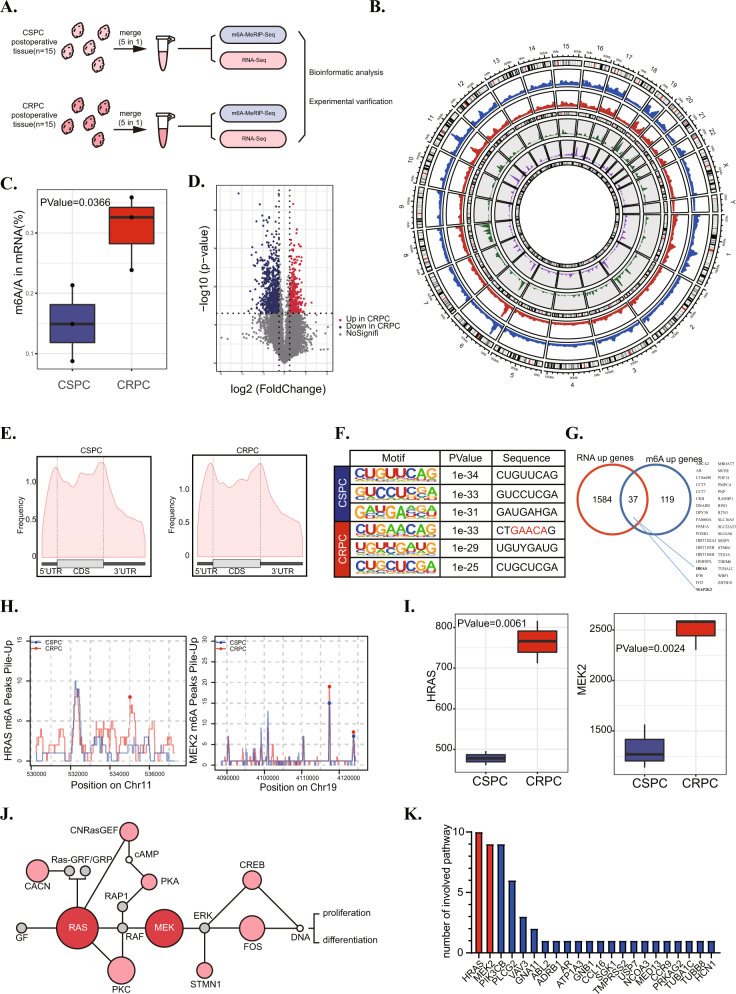
MeRIP-seq of CRPC tumours with enhanced m^6^A methylation. **A** Flow chart of MERIP&RNA sequencing to detect CSPC and CRPC tissue. **B** Overall display of MERIP&RNA sequencing data in the Circos figure. **C** The total m^6^A level of CSPC and CRPC tissue. **D** The volcanoplot shows the results of RNA sequencing of CSPC and CRPC tissues from a global perspective. **E** Metagene showed an m^6^A distribution pattern in CRPC and CSPC tissue. The distribution of m^6^A was determined by MeRIP-seq. **F** The m^6^A motif and sequence enriched in CSPC and CRPC was showed. **G** Venn diagram showing significant overlap among variated genes in CRPC/CSPC and m^6^A enriched genes in CRPC/CSPC. **H** The data of MeRIP-seq data showed the level of m^6^A modification of HRAS and MEK2 in CSPC and CRPC tissues. **I** The results of RNA-seq showed the RNA levels of HRAS and MEK2 in CSPC and CRPC tissues. **J** The figure ranks genes with differences in both m^6^A and RNA levels by the number they are involved in the signaling pathway. HRAS and MEK2 are marked in red in the figure. **K** ERK signal pathway diagram, in which the genes with significant differences in m^6^A and RNA levels in CSPC and CRPC are marked in red, while genes with only RNA levels are labeled in pink.

To investigate whether there is a difference in N6-methyladenosine(m^6^A) modification between castration-resistant prostate cancer (CRPC) and castration-sensitive prostate cancer (CSPC), we collected 30 specimens, including 15 CRPC and 15 CSPC specimens, to perform RNA-seq and MeRIP-seq. All specimens were postoperative tissues and each 5 CRPC or CSPC specimens were mixed into 1 sample to meet the RNA dosage of RNA-seq and MeRIP-seq (Fig. 1A). The overall display of both sequencing results is shown in the circos figure (Fig. 1B). Interestingly, MeRIP-seq showed the level of m^6^A in CRPC tissues was significantly higher than that in CSPC tissues (Fig. 1C). Volcanoplot analyzed from RNA-seq data listed the differential expression genes between CSPC and CRPC (Fig. 1D). The GO analysis of DEGs are shown in Supplementary Fig. 1 (Fig. S1A). Metagene analysis of MeRIP-Seq data showed that m^6^A sites were enriched near start codons and stop codons (Fig. 1E). Motif analysis of CSPC and CRPC was conducted and is shown in Fig. 1F.

To further evaluate the role of m^6^A in CRPC, our group explored and located the signaling pathway regulated by the different m^6^A levels between CRPC and CSPC. MeRIP-sequence and RNA-sequence data were analyzed to screen out the transcripts with both regulated m^6^A modification and expression levels (Fig. 1G). Strikingly, HRas proto-oncogene GTPase (HRAS) and mitogen-activated protein kinase kinase 2 (MAP2K2 or MEK2) were found to have a higher m^6^A level as well as an expression level in CRPC (Fig. 1H, I). MeRIP-qPCR assay was used to verify the m^6^A level of these two genes in CRPC tissues (Fig. S1B). Importantly, these two genes were reported to participate in the extracellular signal-regulated kinase pathway (ERK or MAPK pathway) and play a critical role in the “core region” of the pathway (Fig. 1J) [23]. Genes in Fig. 1. G were ranked and prioritized by the number of KEGG pathways they participated in (Fig. 1K). HRAS and MEK2 are extensively involved in many signaling pathways that promote PCa progression, including the MAPK, AKT, and AR signaling pathways [27–30].

### Clinical analysis confirmed the positive correlation of METTL3 and HRAS/MEK2 in CRPC

**Fig. 2:**
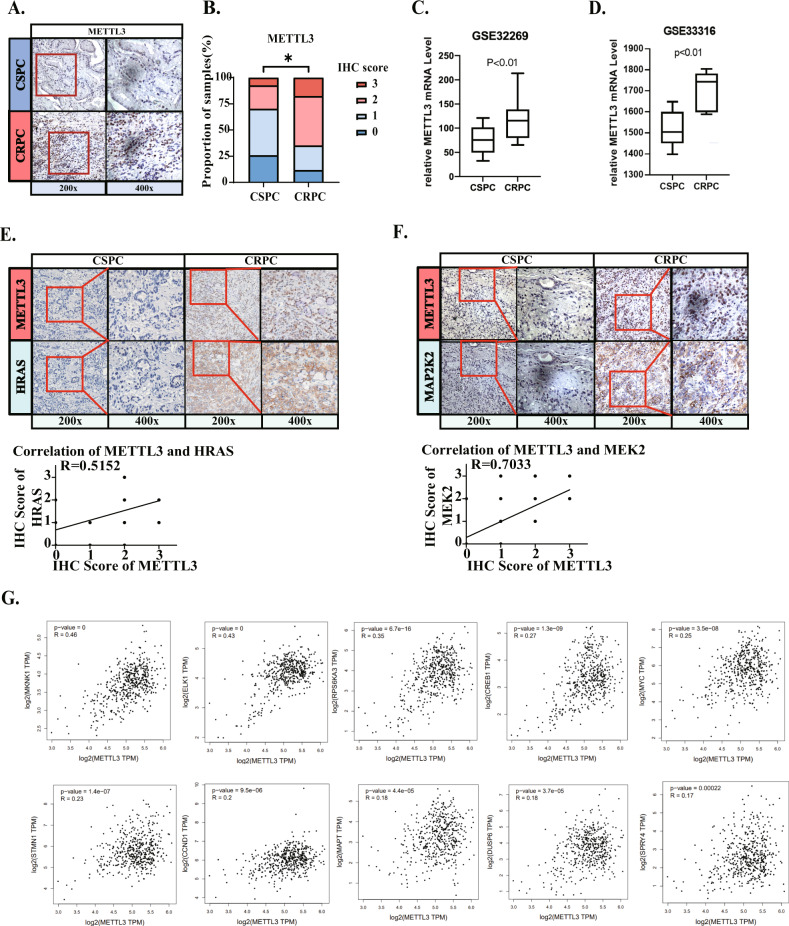
Clinical analysis confirmed the positive correlated of METTL3 and HRAS/MEK2 in CRPC. **A** Images of IHC-stained tissue microarray showed that METTL3 protein levels in CSPC and CRPC. **B** Statistical results of the IHC-stained tissue microarray showing METTL3 protein levels in CRPC vs CSPC. **C**, **D** Visually analyze the transcriptome data from the GEO database to compare the mRNA levels of METTL3 (GSE32269, clinical CRPC/CSPC tissue data and GSE33316, PDX model before vs after castration). **E**, **F** The correlation of METTL3 and HRAS&MEK2 protein expression was verified by an IHC-stained tissue microarray. **G** TCGA database was analyzed and we found that METTL3 and ERK1/2 downstream genes are widely positively correlated at the RNA level.

To evaluate the mechanism leading to the different m^6^A modification levels, 5 tissue microarrays, including 18 CRPC and 30 CSPC tissues, were immunohistochemically stained to detect the protein levels of the m^6^A”writer” and”eraser” (Fig. 2A and Fig. S2A–C). After analyzing the IHC score statistics data, we found that the protein levels of METTL3 and METTL14 were significantly up-regulated in CRPC tissues (Fig. 2B and Fig. S2A–C). Analysis of another human CRPC database (GSE32269, including 22 primary CSPC versus 29 CRPC [31]) found statistically significant increases in METTL3 in CRPC (Fig. 2C). Furthermore, a human prostate cancer xenograft model database analysis (GSE33316, the expression changes after castration of a PDX model [32]) revealed PCa after androgen deprivation exhibited significantly enhanced expression of the METTL3 m^6^A methyltransferase (Fig. 2D). Combining the level of ERK pathway regulation and the dominant function in the methyltransferase complex, we considered that METTL3 makes major contributions to the hyper-activation of the ERK signaling axis in CRPC.

Next, we used PCa microarrays to check the protein level correlation between METTL3 and HRAS/MEK2 in human tissues, and found that METLL3 has a strong correlation with these two genes, confirming the existence of the positively correlation between METTL3 and HRAS/MEK2 in CRPC (Fig. 2E, F). The TCGA database showed that at the mRNA level, METTL3 was positively correlated with almost all ERK downstream genes (Fig. 2G).

### Reduced METTL3 decreases cell proliferation and migration by regulating the ERK pathway

To further validate the function of METTL3 in the progression of CRPC, we used the CSPC cell line LNCap and CRPC cell lines LNCap-AI and C4-2 to perform follow-up experiments. The generation of the LNCap-AI cell line acted as a simulation of the androgen-deprived treatment in clinic. Then, we characterized the m^6^A level, METTL3 expression and phosphorylated-ERK in LNCap-AI cells. The dot-blotting results demonstrated that the total m^6^A level of LNCap-AI was significantly up-regulated compared with that of LNCap (Fig. S2A). The qPCR and western blotting results revealed that the mRNA and protein levels of METTL3 were up-regulated in the LNCap-AI cell line compared with the parental cell line LNCap (Fig. S2B). The ERK signal pathway is known to be constitutively activated in CSPCs after long-term androgen deprivation [33, 34]. In our data, ADT condition caused an up-regulation of phosphorylated ERK (p-ERK) as well as HRAS and MEK2 levels in LNCap-AI cells (Fig. S2C).

**Fig. 3:**
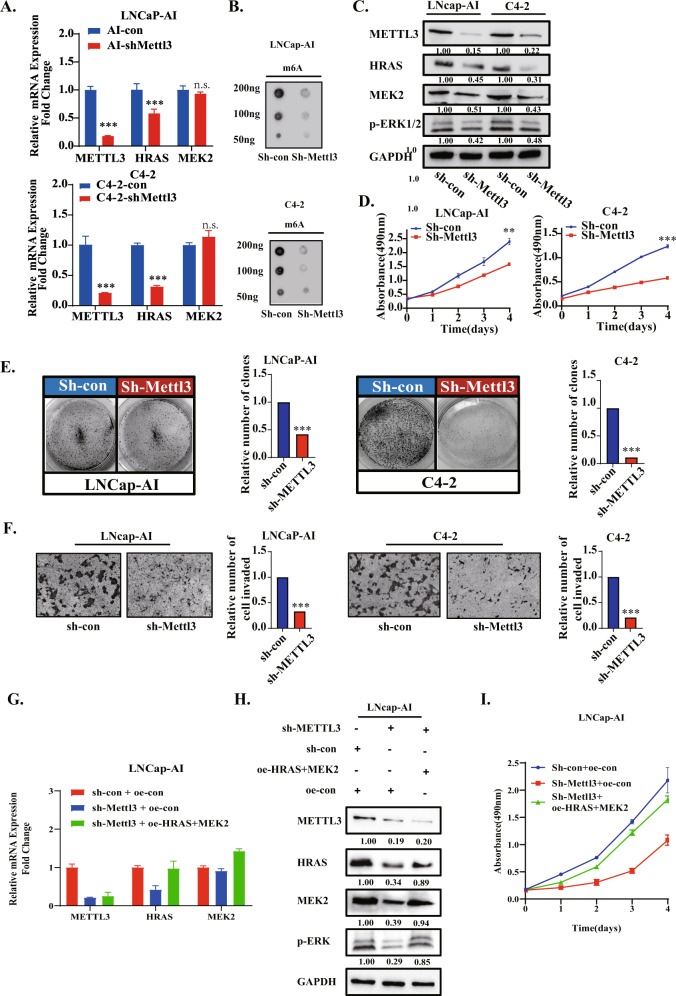
Reduced METTL3 decreases cell proliferation and migration by regulating the ERK pathway. **A** The RNA levels of METTL3, HRAS and MEK2 were detected in LNcap-AI and C4-2 cells respectively transfected with sh-con and sh-METTL3 by RT-qPCR, and GAPDH was used as an internal reference. **B** The m^6^A levels were detected in 4 cell lines (LNcap-AI and C4-2transfected with sh-con, sh-METTL3, respectively) by dot-blotting. **C** The protein levels of METTL3, HRAS and MEK2 and phosphorylation of ERK1/2 were detected in 4 cell lines (LNcap-AI and C4-2 respectively transfected with sh-con, sh-METTL3) by Western blotting, and GAPDH was used as internal reference. **D** An MTT assay was used to detect the changes in cell viability after METTL3 knockdown. **E** The changes in cell proliferation after METTL3 knockdown were detected by plate cloning assay. Statistical results (right side) of the above proliferation assay. **F** Transwell assays were used to detect the changes in cell migration and invasion after METTL3 knockdown. **G**, **H** After overexpression of HRAS and MEK2 in LNCap-AI sh-METTL3 cells, the changes in RNA (**G**) and protein levels (**H**) were detected to determine knockdown efficiency. **I** After overexpression of HRAS and MEK2 in LNCap-AI sh-METTL3 cells, the changes in cell viability were observed by MTT assay.

To further evaluate the importance of METTL3, we knocked down METTL3 by lentivirus in CRPC (LNCap-AI, C4-2 and 22Rv1)cell lines. The mRNA and protein levels of gene HRAS and gene MEK2 were detected by qPCR and Western blotting, respectively. At the same time, the knockdown efficiency of METTL3 is also shown here. The level of phosphorylated ERK was also tested. (Fig. 3A–C, Fig. S2D). Shockingly, we found that knockdown of METTL3 can attenuate the mRNA and protein expression levels of the HRAS gene to various degrees, but for MEK2, only the protein level was affected, and the mRNA level did not change. This suggests that the two genes may be influenced by different mechanisms. We selected two downstream genes (CCND1 and C-fos) for verification, and qPCR results confirmed the regulatory effect of METTL3 on ERK downstream genes (Fig. S2E).

METTL3 has been identified as an oncogene in prostate cancer [35–37]. The results of a series of phenotypic experiments using the CRPC cell line and its stable cell line with METTL3 knockdown also proved this. From the results of the MTT assay (Fig. 3D), clone formation assay (Fig. 3E) and migration and invasion assay (Fig. 3F), we can see that the proliferation, invasion and cell viability of C4-2 and LNCap-AI cells decreased significantly after METTL3 knockdown (Fig. 3D–F). While ablation of METTL3 led to decreased cell growth in LNCaP-AI cells, overexpression of HRAS and MEK2 almost completely restored the proliferation ability of these cells (Fig. 3G–I). Taken together, the results from Fig. 3A–I suggest that METTL3 contributes to HRAS&MEK2-promoted ERK signaling pathway activation and CRPC progression.

### Overexpression of METTL3 rather than catalytic mutation of METTL3 increases cell proliferation and migration by up-regulating the ERK pathway

From a previous experiment, we confirmed the lower expression of METTL3 in LNCaP cells than in LNCaP-AI cells. Then, we further overexpressed METTL3 (oe-M3) and its catalytic mutant form (oe-M3-mut) in the LNCaP cell line. Consistent with our expectations, we found that the mRNA and protein levels of the HRAS were enhanced in oe-M3 cells but not in oe-M3-mut cells (Fig. 3A, C). Correspondingly, the protein level of the MEK2 gene but not the mRNA level, was also up-regulated, and the level of phosphorylated ERK1/2 was significantly increased. Similarly, we can see that in oe-M3 cell lines, the protein translation of MEK2 is also enhanced. These data indicate that METTL3 boosts the HRAS and MEK2 protein expression through its m^6^A “writer” but not other functions, and substantially promotes the phosphorylation of ERK1/2.

**Fig. 4:**
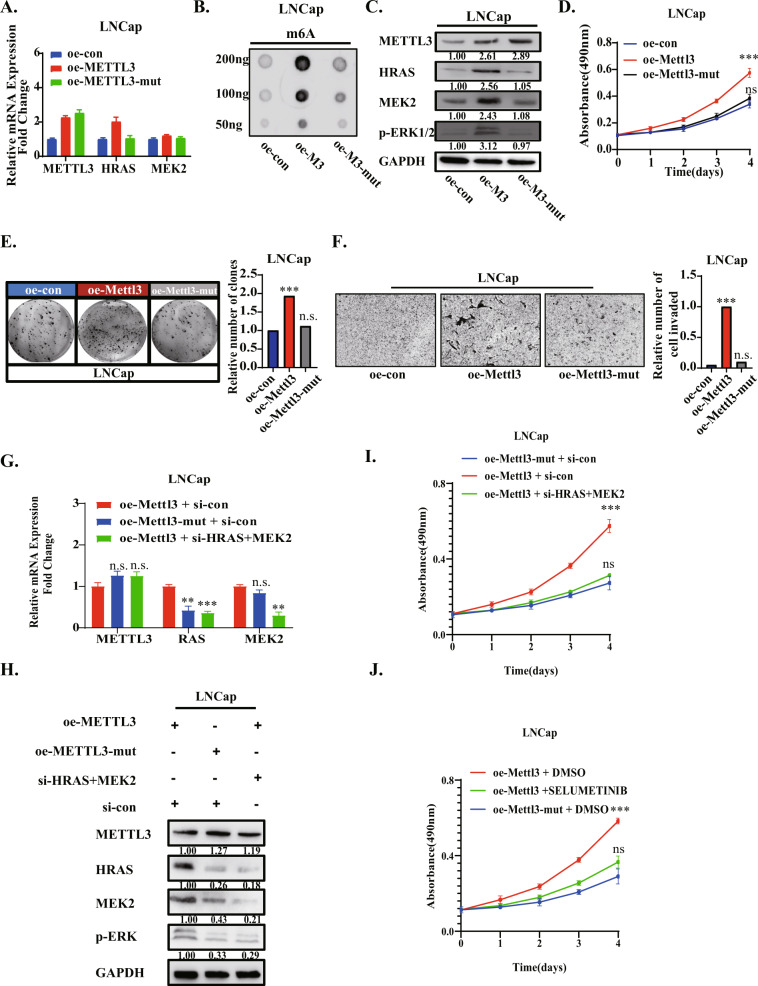
Overexpression of METTL3 rather than catalytic mutation of METTL3 increases cell proliferation and migration by up-regulating the ERK pathway. **A** The mRNA levels of METTL3, HRAS and MEK2 were observed in LNCaP cells overexpressing METTL3 (oe-M3), the catalytic mutant form(oe-M3-mut) and the control group (oe-con). **B** The m^6^A levels were detected in LNCaP overexpressing METTL3 (oe-M3), catalytic mutant form (oe-M3-mut) and control group (oe-con) cell lines by dot-blotting. **C** The protein levels of METTL3, HRAS, MEK2 and phosphorylation of ERK1/2 were detected in LNCaP overexpressing METTL3 (oe-M3), catalytic mutant form (oe-M3-mut) and control group (oe-con) by Western blotting, and GAPDH was used as internal reference. **D** An MTT assay was used to detect the changes of cell viability after overexpression of METTL3 and its catalytic domain mutants. **E** The changes of cell proliferation after overexpression of METTL3 and its catalytic domain mutants were detected by plate cloning assay. **F** Transwell assays were used to detect the changes of cell migration and invasion ability after overexpression of METTL3 and its catalytic domain mutants. **G**, **H** After knockdown of HRAS and MEK2 in LNCap oe-METTL3, the changes of RNA (**G**) and protein levels (**H**) were detected to determine knockdown efficiency. **I** After knockdown of HRAS, MEK2, HRAS & MEK2 in LNCap oe-METTL3, the changes of cell viability were observed by MTT assay. **J** After treatment selumetinib (MAPK pathway inhibitor) in LNCap oe-METTL3, the changes of cell viability compared with LNCap oe-METTL3-DMSO and LNCap oe-METTL3-mut were observed by MTT assay.

Similarly, we conducted a series of phenotypic experiments using the stable cell line of the CSPC cell line overexpressing METTL3 and the catalytic domain mutant METTL3 (Fig. 4E–G). The results showed that METTL3, rather than the m^6^A catalytic domain mutant METTL3, can promote the cell viability, cell proliferation and invasion ability of LNCap cells. Furthermore, we knocked down HRAS and MEK2 in cell lines overexpressing the METTL3 or METTL3 catalytic mutant form (Fig. 4G, H), and found that after knocking down these two genes, the proliferation ability enhanced by overexpression of METTL3 was completely restored (Fig. 4I). We also used the ERK pathway inhibitor selumetinib to perform phenotypic recovery experiments and achieved the same effect (Fig. 4J). Overall, we can know that higher METTL3 expression augments the HRAS and MEK2 genes by regulating m^6^A, then activating the ERK pathway and promoting the malignant cell phenotype.

### The m^6^A modification of HRAS and MEK2 mediates different mechanisms to regulate their respective protein levels

**Fig. 5:**
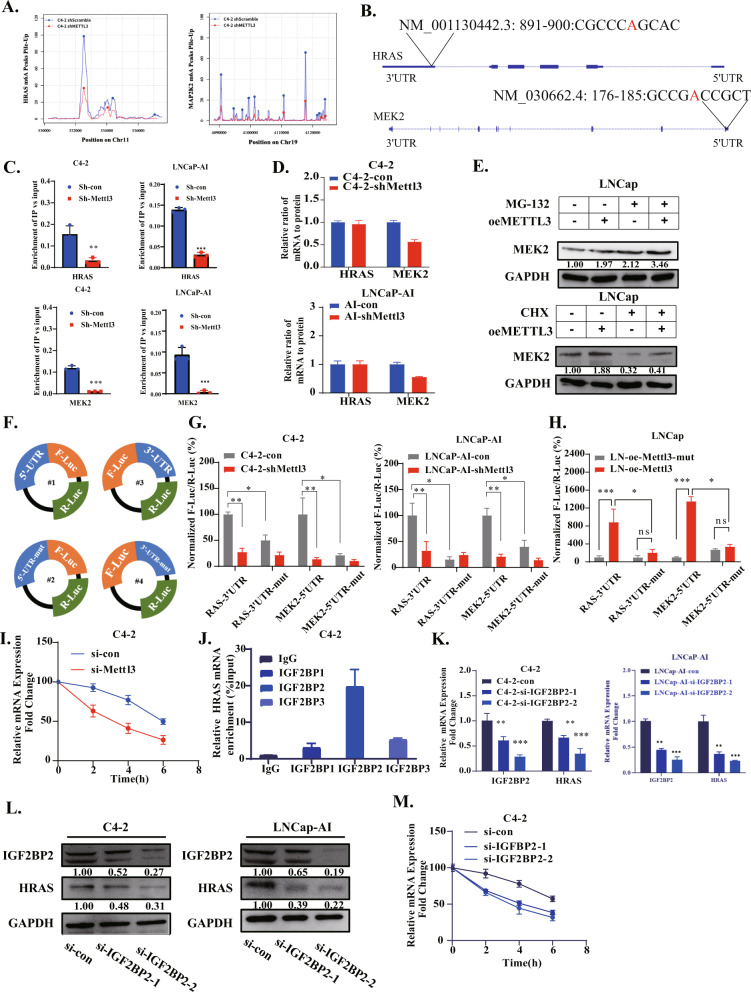
The m^6^A modification of HRAS and MEK2 mediates different mechanisms to regulate their respective protein levels. **A** The results of MERIP-seq performed by C4-2 sh-con and C4-2 sh-METTL3 cell lines showed that the m^6^A modification sites of HRAS and MEK2. **B** Schematic representation of m^6^A position of HRAS and MEK2. **C** The results of MeRIP-qPCR showed that the enrichment of HRAS and MEK2 genes changed after METTL3 knockdown in C4-2/LNCap-AI cell line. **D** The relative ratio of protein to mRNA of HRAS and MEK2 genes in 4 cell lines. **E** After MG-132 and CHX were used to treat the two cell lines, respectively, the protein level of MEK2 was detected. **F**. Schematic representation of luciferase plasmid containing 3’UTR of HRAS, 5’UTR of MEK2 and their mutants. **G** After transfection of four plasmids in C4-2/LNCap-AI sh-con and C4-2/LNCap-AI sh-METTL3 cell lines for 24 h, fluorescence strength of luciferase were detected. **H** After four plasmids were transfected into LNCap oe-METTL3 and LNCap oe-METTL3-mut cell lines for 24 h, the fluorescence intensity of luciferase was detected. **I** After Act-D inhibited transcription, the RNA level of HRAS was determined after knocking down METTL3 for 2, 4 and 6 h. **J** RIP-qPCR was performed with IGF2BP1/2/3 antibody, and the RNA enrichment level of HRAS was detected. **K**. After knockdown of IGF2BP2, the RNA level of HRAS gene changed in C4-2. **L** After IGF2BP2 knockdown, the protein level of HRAS gene changed in C4-2. **M** After act-D inhibited transcription, the RNA level of HRAS decreased after IGF2BP2 knockdown, which indicated that IGF2BP2 knockdown could reduce the stability of HRAS mRNA.

In further experiments, we aimed to determine the different mechanisms by which METTL3 regulates HRAS and MEK2. The protein translation of MEK2 is regulated by METTL3, while for HRAS, METTL3 directly affects its mRNA expression. We supposed this may be because of the alternative biofunctions of m^6^A in different mRNA regions, hence, we performed MeRIP-sequencing (using the C4-2 and C4-2 sh-M3 cell lines) to detect m^6^A peak sites. As previously predicted, after knocking down METTL3, the m^6^A levels of the HRAS and MEK2 genes decreased significantly (Fig. 5A). Interestingly, we found that the m^6^A peaks of HRAS and MEK2 are situated at various positions on their transcripts (Fig. 5A). The highest m^6^A peak of HRAS is located at the 3’UTR, but MEK2’s peak is located at the 5’UTR (Fig. 3B), which may lead to diverse molecular functions. MeRIP-qPCR assays were performed on C4-2 vs C4-2 sh-M3 cells and LNCap-AI vs LNCap-AI sh-M3, and the position of m^6^A peak of HRAS and MEK2 was verified again (Fig. 5C).

Furthermore, we calculated the relative ratio of mRNA to protein (using the ratio of quantified WB grayscale values to the relative RNA expression levels detected by qPCR) of these two genes and found that after knocking down METTL3, the relative ratio of mRNA to protein of MEK2 was significantly down-regulated, while the relative ratio of mRNA to protein of HRAS showed no significant difference (Fig. 5D). It has been reported that the m^6^A peak in 5’UTR region can promote cap-independent translation [7]. Then, MG-132 and CHX (lysosome and proteasome inhibitors) were used to test the protein translation of MEK2. Our data showed that when protein degradation was inhibited, MEK2 protein level was significantly up-regulated after overexpression of METTL3 (Fig. 5E), indicating that the up-regulation of protein was not due to the reduced degradation but to increased of protein translation.

Furthermore, we mapped these two locations of the m^6^A peak across the human RNA transcriptome to construct luciferase reporter gene plasmids. Dual luciferase reporter gene experiments have often been used to reveal the specific mechanism of m^6^A modification affecting protein translation in previous studies. Our group inserted the original or mutated sequence of MEK2’s 5’-UTR into the site before the CDS region of the firefly luciferase gene (F-luc), and the mutation site was the m^6^A peak. Similarly, the wild-type or mutated 3’-UTR of HRAS was inserted into the site after the CDS of the luciferase to test the biofunction of m^6^A peaks. Both plasmids contained the Renilla luciferase (R-luc) gene as an internal reference (Fig. 5F). The results of the dual-luciferase reporter gene assay showed that the fluorescence was significantly decreased after mutated the m^6^A peak (Fig. 5G). Moreover, there was no significant difference between transfection of mutant and nonmutant plasmids in the METTL3 knockdown cell line, which shows that METTL3 plays a decisive role in the regulation of m^6^A(Fig. 5H). Thus far, we have confirmed that the m^6^A peak in the 5’-UTR of MEK2 can significantly regulate protein translation, which may be related to m^6^A-mediated 5’ cap-independent translation. Furthermore, we tried to explore how the mRNA level of the HRAS gene is regulated by m^6^A. Actinomycin-D (ACT-D) is a transcription inhibitor. After administering Actinomycin-D to the C4-2 cell line, we administered si-METTL3, and then observed the degradation rate of the HRAS gene. After knocking down METTL3, the degradation rate of HRAS mRNA was significantly faster than that of the control group (Fig. 5I). We speculate that METTL3 regulates the mRNA stability of the HRAS gene by regulating m^6^A. This is consistent with reports in the literature that the m^6^A site located in the 3’-UTR can mediate mRNA stability [7, 38].

IGF2BPs are a group of proteins that read m^6^A, including the three proteins IGF2BP1/2/3. It has been reported that this group of reading proteins can bind to m^6^A and stabilize its mRNA. The RIP experiment was used to detect these three proteins separately and it is found that IGF2BP2 showed more binding in the 3’UTR region of the HRAS gene than the other two proteins (Fig. 5J). Furthermore, we used a pair of siRNAs to knock down IGF2BP2 and found that the mRNA and protein levels of HRAS were also significantly down-regulated after knockdown (Fig. 5K, L).We further examined the degradation rate of HRAS mRNA after knockdown of IGF2BP2. We found that the degradation rate was significantly faster than that of control group (Fig. 5M). In conclusion, we confirmed that in CRPC, METTL3 mediates the changes in RNA stability and protein translation by regulating the m^6^A modification of HRAS and MEK2 mRNA, and then affects the activation of the ERK pathway.

### High METTL3 expression is involved in the development of resistance to enzalutamide

**Fig. 6:**
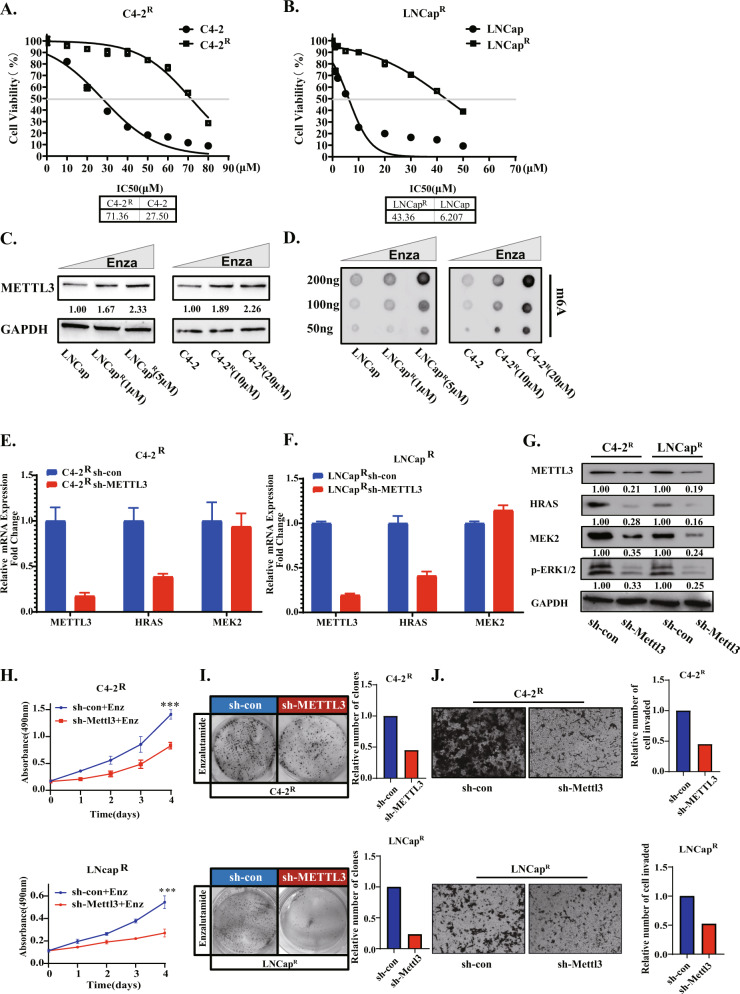
High METTL3 expression is involved in the development of resistance to enzalutamide. **A**, **B** Cell viability assay following treatment for 48 h with indicated concentrations of enzalutamide in C4-2/C4-2R and LNCap/LNCap^R^ cell lines. **C** Western blotting showed the protein alteration of METTL3 in long-term enzalutamide treated in LNCap and C4-2 cell lines. **D** The total m^6^A levels of C4-2 and C4-2^R^ cell lines were detected by dot blotting assay. **E**–**G** qPCR and Western blotting showed the mRNA and protein levels of METTL3, HRAS, MEK2 and phosphorylation of ERK between C4-2^R^ sh-con/LNCap^R^ sh-con and C4-2^R^ sh-METTL3/LNCap^R^ sh-METTL3 cell lines. **H** An MTT assay was used to detect the changes of cell viability before and after METTL3 knockdown in the C4-2^R^ and LNCap^R^ cell lines. Cells were cultured with enzaluamide (20 nm). A *P* value of < 0.05 was considered significant. ***represents *P* < 0.001. **I** The cell proliferation of C4-2^R^ and LNCap^R^ cell lines before and after METTL3 knockdown was detected by plate cloning assay. **J** Transwell assays were used to detect the migration and invasion abilities of C4-2^R^ and LNCap^R^ cells before and after METTL3 knockdown.

In the process of PCa establishing resistance to enzalutamide, the activation of the ERK signaling pathway has been proven to be an important factor contributing to resistance [22, 23]. Since METTL3 has the function of activating the ERK pathway, we explore whether it played a role in the enzalutamide-resistance cell line. First, we used the C4-2 and LNCap cell lines to construct enzalutamide-resistant cell lines C4-2^R^ and LNCap^R^, and the method of cell line construction is detailed in previous articles [23, 39]. Comparing the IC_50_ of enzalutamide between C4-2^R^/LNCap^R^ and C4-2/LNCap confirmed the resistance to enzalutamide (Fig. 6A, B). Previous reports showed that p-ERK1/2 was up-regulated in enzalutamide-resistant cells [22, 23]. We detected the level of METTL3 and m^6^A in enzalutimide-resistant cells and sensitive cells, and found that the level of METTL3 and m^6^A in drug-resistant cell lines was significantly up-regulated (Fig. 6C, D). We also constructed stable cell lines by lentivirus(sh-METTL3). Then, we tested the change of HRAS/MEK2/p-ERK signaling axis by qPCR and Western blotting (Fig. 6E–G). The changes in cell viability, invasion and proliferation ability of the C4-2^R^ and LNCap^R^ before and after knockdown METTL3 was also detected (Fig. 6H–J). As expected, phenotypic experimental data showed that knocking down of METTL3 reduced the tolerance to enzalutamide. In addition, we also administered enzalutamide to LNcap-AI cells and C4-2 cell lines knocked down METTL3 and their respective control groups and found that the knockdown group had weaker enzalutamide tolerance (Fig. S4A, B). After over-expressed of METTL3 in the LNCap cell line, and then treatment with a lower concentration of enzalutamide (5 μM), the MTT assay showed that its resistance to enzalutamide was enhanced (Fig. S4C).

### ASO targeting METTL3 combined with enzalutamide inhibits the proliferation of enzalutamide-resistant PCa in vitro and in vivo

**Fig. 7:**
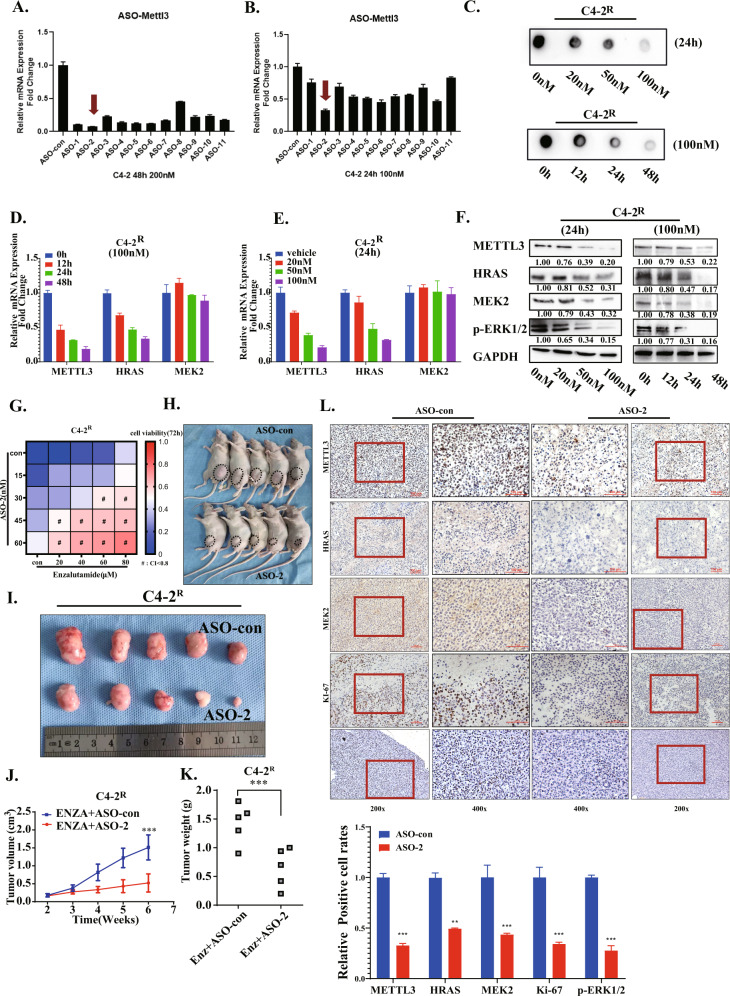
ASO targeting METTL3 combined with enzalutamide inhibits the proliferation of enzalutamide-resistant PCa in vitro and in vivo. **A** qPCR results showed the mRNA level of METTL3 transfected with 10 candidate ASO sequences in C4-2R cell line. ASOs were transfected at a concentration of 200 nm and RNA samples were harvested 48 h after transfection. **B** qPCR results showed the mRNA level of METTL3 transfected with 10 candidate ASO sequences in C4-2R cell line. ASOs were transfected at a concentration of 100 nm and RNA samples were harvested 24 h after transfection. **C** Dot blotting assay showing the total RNA m^6^A level of C4-2R cells transfected with ASO-2 at different concentrations and for different times. **D**, **E** qPCR assays showed the mRNA levels of METTL3/HRAS/MEK2 in C4-2R cell line after transfection with ASO-2 at different concentrations and for different times. **F** Western blotting showed the protein levels of METTL3/HRAS/MEK2 in C4-2R cell line after transfection with ASO-2 at different concentrations and for different times. **G**. Heatmap summarizing the combination index (CI) of ASO-2 and enzalutamide in C4-2^R^ cell line. (CI less than 0.8 was marked with an #). **H** Xenograft tumor experiment were carried out in nude mice using C4-2^R^ cell line. After tumor formation, ASO-2 or ASO-con was given to different groups, while the enzalutamide was given to all mice. This image shows the nude mice bearing tumor after being killed at the sixth week. **I** A photo of xenograft tumors in nude mice after dissection. The upper part was the control group, while the lower part shows the ASO-2 treatment group. **J** The curve depicts the volume change of xenograft tumors in nude mice of control group and ASO-2 experimental group.***represents *P* < 0.001. **K** The scatter plot depicts the weight of xenograft tumors in nude mice of control group and ASO-2 experimental group.***represents *P* < 0.001. **L** IHC staining showed the protein levels of METTL3, HRAS, MEK2, p-ERK1/2 and Ki-67 in xenografts of control group and ASO-2 experimental group. Statistical analysis are shown below. A *P* value of < 0.05 was considered significant. *represents *P* < 0.05, **represents *P* < 0.01 and ***represents P < 0.001.

There are currently few studies on targeting METTL3 by ASO to delay cancer progression. We used an online RNA-motif prediction tool (Sfold) to screen and verify a series of 20 bp ASO sequences specifically targeting METTL3 mRNA [40–43]. We selected 10 candidate ASOs (S Table 3), including desired motifs [44] and modified them into the second generation “GAPMER” style (details of modification are described in method). We transfected these 10 ASOs into enzalutamide-resistant cell lines (C4-2^R^) and used qPCR experiments to detect their knockdown efficiency (Fig. 7A, B). Thereby, the NO.2 ASO sequence candidate (ASO-2) with the strongest ability to reduce the mRNA level of METTL3 was selected for the following experiment. We used ASO-2 to treat the C4-2^R^ cell line and observed that it reduced the overall m^6^A level in a concentration- and time-dependent manner (Fig. 7C). In addition, the C4-2^R^ cell line was treated with an ASO-2 gradient for 24 h, and we found that the mRNA and protein reductions in METTL3 and ERK signaling axis genes detected above were negatively correlated with ASO-2 concentration and time, except for the mRNA level of MEK2 (Fig. 7D–F). The results of the MTT experiment showed the drug combination index (CI) of ASO-2 and enzalutamide at different concentrations (Fig. 7G). This result showed the potential of ASO-2 as a combination therapy for enzalutamide-resistant PCa cells.

To further evaluate the effect of ASO-2 in vivo, we used a nude mouse xenograft tumor model to construct an animal model of enzalutamide resistance. Two weeks after xenograft tumor inoculation, we administered both enzalutamide and ASO-2. We found that the tumor proliferation rate of the combined treatment group was significantly lower than that of the enzalutamide-only group (Fig. 7H to K). In addition, we checked the reduction in METTL3 expression by ASO-2 with immunohistochemical staining and found that the level of METTL3 protein in tumors in the combined treatment group was significantly reduced, and the protein level of HRAS/MEK2/KI-67/p-ERK1/2 was also significantly down-regulated (Fig. 7L). All data above confirmed that ASO-2 can decrease METTL3 expression and mitigate the overactivation of the ERK signaling pathway in vitro and in vivo, thereby achieving the enzalutamide-resistant cell proliferation inhibition.

## Discussion

**Fig. 8:**
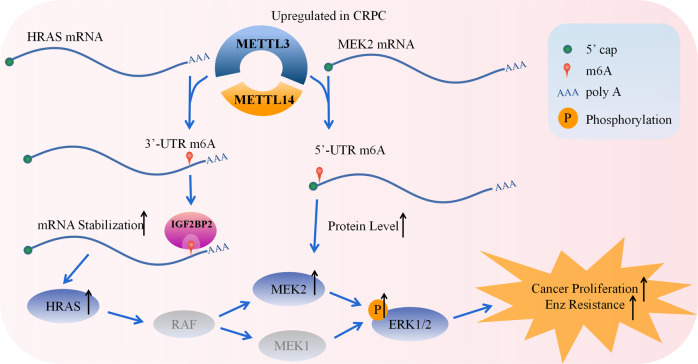
Schematic describing how the upregulated METTL3&14 in CRPC activated the ERK pathway by regulating protein levels of HRAS and MEK2. METTL3 enhances RNA stability of HRAS and enhances protein translation of MEK2 to activate the ERK pathway.

METTL3 has been widely studied and reported to play diverse roles in the onset and proliferation of tumors, and its biofunctional spectrum in human cancers has gradually been completed in recent years [45–47]. Specific to urogenital tumors, the carcinogenesis of METTL3 in bladder cancer has been deeply uncovered [48, 49], but there is still a controversy in kidney cancer [50–52]. In testicular carcinoma, the pro-oncogenic effect of m^6^A methylation has also been analyzed both preclinically and clinically [53, 54]. In prostate cancer, the literature has confirmed that METTL3 is significantly up-regulated in cancer tissues compared to normal prostate tissues [35]. In addition, we found that most studies suggest that METTL3 is not only correlated with the malignant transformation, but also activated in the onset of cancer metastasis [12, 36, 37]. Our data further revealed that when it progressed to the CRPC stage, METTL3 was further increased and exerted its effect, resulting in resistance to AR targeting therapy drugs (such as enzalutamide) (Fig. 7). Although previous studies have discussed the relationship between RNA methylation and cancer drug resistance in other tumors, the current study could be a milestone to complete this process as we first discovered and confirmed the role of RNA methylation in CRPC. The research group conducted a series of cell phenotyping experiments, which proved that cell viability, cell proliferation, migration, invasion and drug resistance acquirement were significantly reduced after METTL3 knockdown by shRNA in the CRPC cell lines Fig. 8. The above experimental results, when combined strongly support the conclusion that METTL3 is an oncogene in CRPC. Some scholars, such as Cotter, mentioned that METTL3 may have a tumor suppressor effect when prostate cancer cells transdifferentiate to NEPC [55]. Our experimental data first clearly and systematically indicated that METTL3 has a significant cancer-promoting effect at least in the CRPC stage.

In recent years, a large number of investigations have discovered a relationship between m^6^A modification and epigenetic regulation, including transcriptional regulation [8], histone modification [9], and noncoding RNA expression [11, 12]. Moreover, m^6^A has been reported to deal with RNA expression by regulating the stability and alternative splicing of transcripts [7], or to influence protein generation by regulating the translation efficiency [10]. In our study, we verified that the changes in m^6^A in the HRAS 3’-UTR and MEK2 5’-UTR regions have distinct effects on the RNA of these two genes through a dual luciferase reporter assay. Through the results of MeRIP-qPCR, we confirmed that m^6^A in the 3’-UTR of HRAS enhances the RNA stability through IGF2BP2. We combined the results of luciferase assay and qPCR in order to study the relative ratio of mRNA to protein. The experimental data show that the change in of m^6^A in the 5’-UTR of MEK2 can change its protein translation. The above mechanism caused the upregulation of HRAS and MEK2 at the protein level and jointly promoted the phosphorylation of ERK.

In colorectal cancer, Peng showed that METTL3 affects the ERK pathway by regulating the miR-1246/SPRED2 signaling axis and enhancing the invasion ability of cancer [56]. In gastric cancer, m^6^A modification activated the ERK pathway by inhibiting BATF2 [57]. Furthermore, it has been reported that ERK can mediate the phosphorylation of METTL3 and regulate its stability [58]. Although the relationship between METTL3 and the ERK signaling pathway in cancer has not been established for the first time, a very limited number of studies have been carried out in the past to reveal how METTL3/ERK axis promotes the progression of drug resistance in PCa. Similarly, the connection between METTL3 and the AKT pathway has also been described in PCa, but not in the CRPC stage [35]. Combined with our experimental data and the upregulating of METTL3 by ERK [58], we assumed that METTL3 and ERK may form a positive feedback loop in prostate cancer, thereby promoting cancer progression. We considered that METTL3 is indispensable in CRPC (especially after ADT) since it can activate downstream genes, such as ERK, AKT or MYC pathways, which could succeed the blocked AR signaling axis to promote proliferation. In addition, some of our data reveal that METTL3 is likely to be involved in the hyperactivation of some genes related to cell stemness, such as YAP1 (Additional file 1). The relevance between these genes and METTL3 has been reported in other malignant tumors, and its specific mechanism in PCa may be discussed later by our group.

Recently, with the progress of ASO-related pharmacological research [59–62], many ASO drugs have been approved by the FDA to enter the market [63–66]. The associated preclinical studies in prostate cancer treatment have also increased, including targeted AR signaling pathways (including AR variants) [67, 68] and non-AR signaling pathways [69, 70]. Specific small molecule inhibitors of METTL3 (STM2457) have been discovered [71]. Nevertheless, the ASO sequences targeted METTL3 mRNA screened by our group still have their clinical value, and the side effects of the small molecule inhibitors have not been investigated systematically. ASOs exhibit higher target specificity with fewer side effects and better biocompatibility due to their wide presence in organisms [72]. Inherently, the adverse reactions caused by nucleic acid drugs are tiny and consistent no matter how the ASO base sequence changes. In general, there are two kinds of mechanisms by which ASOs play their biological role inside the cell [73], including non-RNA-degradation (such as switch-splicing and exon jumping) [68, 74, 75] and RNaseH-mediated RNA degradation [69, 70, 76–78]. In our study, we selected ASOs that can mediate RNA degradation to reduce the mRNA level of METTL3 in cells. In regard to ASO modification, we used the classic second-generation GAPMER strategy [61], which has been verified by clinical trials in PCa [63], to increase the stability and targeting of ASOs. With the improvement of tumor-targeting nano-ASO delivery systems [79, 80], the clinical potential of ASO application in cancer therapy will become a very promising clinical strategy.

In summary, we confirmed that there was a high level of m^6^A modification of RNA in CRPC, which was mainly caused by the up-regulation of METTL3. Further experiments showed that METTL3 mediated the progression of CRPC by activating the ERK signaling pathway. Our results show that METTL3 promotes the resistance of prostate cancer cells to ADT drugs such as enzalutamide. Combined with the data from ASO animal experiments, we believe that METTL3 may become a potential target for the treatment of CRPC in the future.

## Materials and Methods

### RNA-Seq analysis, MeRIP-Seq and data analysis

Total RNA from the transfected C4-2 cells and tissue samples was extracted with TRIzol (Invitrogen). Then, mRNA sequencing and m^6^A sequencing were synchronously performed (Cloud-Seq Biotech, Shanghai, China). Briefly, 500 ng fragmented mRNAs were saved as input control for RNA-Seq, and 5 μg of fragmented mRNAs were incubated with 5 μg of anti-m^6^A polyclonal antibody (Synaptic Systems, 202003) in IP buffer (150 mM NaCl, 0.1% NP-40, 10 mM Tris-HCl, pH 7.4) for 2 h at 4 °C. The mixture was then immunoprecipitated by incubation with protein-A beads (Thermo Fisher) at 4 °C for an additional 2 h. Then, bound mRNAs were eluted from the beads and then extracted with TRIzol reagent (Thermo Fisher) according to the manufacturer’s instructions. Purified mRNAs were used for RNA-Seq library generation with the NEB Next Ultra TM RNA Library Prep Kit (NEB). Both the input samples (without immunoprecipitation) and the m^6^A IP samples were subjected to 150 bp paired-end sequencing on an Illumina HiSeq sequencer.

Paired-end reads were harvested from Illumina HiSeq 4000 sequencer, and quality was controlled by Q30. After 3’adaptor-trimming and low-quality reads removing by cutadapt software (v1.9.3), the reads were aligned to the reference genome (UCSC MM10) with Hisat2 software (v2.0.4). Methylated sites on RNAs (peaks) were identified by MACS software. Nonmatching methylated sites on RNAs were identified by diffReps. These peaks identified by both software overlapping with exons of mRNA were figured out and chosen by homemade scripts.

The original MeRIP sequencing data has been uploaded to NCBI Gene Expression Omnibus database, and the accession number is GSE221961.

### Immunohistochemistry, tissue microarray analysis and tissue specimens

Tissue microarrays encompassing 28 CSPC tissues and 13 CRPC tissues were designed and manufactured by OUTDO BIOTECH (Shanghai, China). The prostate tissue specimens used in this study were surgical specimens from patients underwent complete clinicopathological. CSPC specimens (*n* = 31) were acquired by radical prostatectomy, and CRPC specimens (*n* = 18) were acquired by transurethral resection of the prostate. Formalin-fixed paraffin-embedded (FFPE) sections of 5 mm thickness were prepared on charged glass slides. After deparaffinization and rehydration, for METTL3 (ab195352), HRAS (ab86696), MEK2 (ab32517), WTAP (ab195377), ALKBH5 (ab195377), FTO (ab124892), heat-mediated antigen trieval was carried out with 10 mM sodium citrate (pH=6), for METTL14 (ab220030), antigen retrieval was carried out with 10 mM Tris base, 1 mM EDTA (pH=9). Endogenous peroxidase activity was blocked by adding 3% hydrogen peroxide. The sections were incubated with diluted antibodies followed by polymerconjugated horseradish peroxidase in a humidified chamber. Standard DAB staining was performed for chromogenic detection of the IHC targets. All tissues were assigned a score based on staining intensity in the epithelial compartment (0, no staining; 1, low positive; 2, positive;3, high positive). All the stained specimens were reviewed, calibrated and optimized by strictly following the protocols from our previously study. All studies were approved by the Ethics Committee of the Second Hospital of Tianjin Medical University,and informed consent was obtained from all patients.

### Cell culture, cell lines and transfection

The LNCaP and C4-2 cells used in this study were purchased from ATCC (Manassas, VA, USA) and cultured in RPMI-1640 medium (Gibco, Waltham, MA, USA) supplemented with 10% fetal bovine serum (Gibco) and 1% penicillin. For androgen deprivation, parental LNCaP cells were cultured in RPMI 1640 medium supplemented with 10% charcoal-stripped fetal bovine serum (BI, Cromwell, CT, USA). The LNCaP-AI line was generated following long-term culture of the parental LNCaP cells under androgen-deprived conditions [24]. LNCaP^R^ and C4-2^R^ cells were generated by culturing LNCaP/ C4-2 cells under increasing Enzalutamide concentrations from 1 to 10 μM for LNCap and 10 μM to 40 μM for C4-2 (every 20d) for 3 month [81]. After generation, LNCaP^R^/C4-2^R^ were maintained in media with 5/15 μM Enzalutamide. Transfection of LNCaP, LNCaP-AI, C4-2, C4-2^R^ and LNCaP^R^ cells reaching 50–70% confluency with siRNA or plasmid constructed was performed by using Lipofectamine 3000 (Invitrogen,Waltham, MA, USA) and X-treme GENE HP Transfection Reagent (Roche, Indianapolis, IN, USA), respectively, according to the manufacturers’ instructions. For the construction of stable cell lines, we used lentiviruses ordered from Sheweisi Inc, Tianjin, China. Before transfection, cells were passaged into T25 cell culture flasks. When the cells were adherent and the degree of cell confluence was approximately 60%, 20ul of the lentivirus solution was added to the medium. Sequences for the siRNAs and shRNAs are listed in S Table2.

### RNA isolation and RT-qPCR

Total RNA was isolated from cells using TRIzol reagent (Invitrogen), RNA concentration was measured by ultraviolet absorbance at 260 nm and used for the first strand cDNA synthesis with the Reverse Transcription System (Roche) following the manufacturer’s protocol.

The obtained cDNA was then analyzed by PCR using Applied Biosystems 7900 Real-Time PCR System (Thermo Scientific) and SYBR Green PCR Master Mix (Roche) according to the manufacturers’ instructions. GAPDH was used as an internal control. The relative expression of RNAs was calculated using the comparative Ct method.Primer sequences are listed in the sequence table (S Table1).

### m^6^A dot blot assay

Total RNA were extracted from the PCa cells with Trizol reagent (Invitrogen), RNA concentration was measured by ultraviolet absorbance at 260 nm. The RNA obtained was diluted to 100/50/25 ng/μl, after which 2 ul of the diluted RNA was separately added onto the nitrocellulose filter membrane (Invitrogen). After being dried, RNAs on the nitrocellulose filter membrane were UV cross-linked in a Ultraviolet Crosslinker. The cross-linked membrane blocked with 5% skim milk at room temperature for 1 h. After being washed for 3 times, the cross-linked membrane was incubated with m^6^A antibody overnight at 4 °C. After washing three times, membrane was incubated with horseradish peroxidase (HRP)-conjugated secondary antibodies (1:10,000 dilution) for 1 h at room temperature. The secondary antibody was washed with TBST and prepared for exposure.

### Western blot analysis

Total cellular proteins were extracted using RIPA buffer (Thermo Scientific, 89,901). Approximately 50 μg of total protein was separated on sodium dodecyl sulfate-polyacrylamide gel electrophoresis (SDS-PAGE), transferred onto polyvinylidene fluoride (PVDF) membranes, blocked with 5% skim milk at room temperature for 2 h, after washed 3 times with TBST the primary antibody was applied and incubated overnight at 4 °C. After washed three times, the membrane was incubated with horseradish peroxidase (HRP)-conjugated secondary antibodies (1:10,000 dilution) for 1 h at room temperature. The secondary antibody was washed with TBST and prepared for exposure. The prepared developing solutions A and B are mixed in proportion (Immobilon Western, Chemiluminescent HRP Substrate, Millipore Corporation, Billerica, MA, USA), and the mixed liquid is dropped on the corresponding molecular weight strip of the PVDF film and placed in an exposure machine for exposure.

The expression levels of target genes relative to reference genes were calculated according to the gray values of all WB bands (software: Tanon Gis). The quantized values of all WB bands were labeled below the bands.

### Cell proliferation, colony formation and invasion

Cell proliferation was tested by MTT kit (Sigma, USA) according to previous studies [24]. For colony formation, about 10^3^ cells (trail set and control set) were seeded in six-well plate, with three replicates. At week 2, colonies were stained with 0.5% crystal violet, imaged and counted. Cell invasion was examined by transwell invasion assay according to previous studies [26].

### Animal studies

The animal studies were approved by Tianjin Institute of Urology (Tianjin, China). 10 Male BALB/c nude mice (7 weeks old) were purchased from SPF Biotechnology co. Ltd. (Beijing, China). In animal experiments, nude mice were randomly divided into two groups. Subcutaneous tumor growth assays were performed with C4-2/C4-2 sh-METTL3 or C4-2^R^/C4-2^R^ sh-METTL3 cells (10^7^ cells per capital). The control group (*n* = 5) and the treatment group (*n* = 5) were injected with PCa cells in 100 μl PBS with 100 μl of Matrigel matrix (BD Bioscience). The growth of tumors was monitored weekly by measuring tumor size from the outside of mouse skin. The investigator measured tumors blinding to group allocation during experiments. The volume was calculated with V = 1/2× larger diameter × (smaller diameter)^2^. After 6 weeks,the difference in tumor size in these two groups was captured and measured, and the tumors were harvested under standard, institutionally approved processes. Tumor samples were paraffin fixed and processed for immunohistochemistry analysis.

### RNA immunoprecipitation (RIP)

RIP experiments were performed using the Magna RIP RNA-binding protein immunoprecipitation kit (Millipore, Billerica, MA, USA) and the m^6^A antibody (Abcam, Cambridge, MA, USA) following the manufacturer’s protocol.

Briefly, cells (1×10^7^) were lysed with ice-cold RIP Lysis Buffer. Collect and store the cell lysate at -80 °C. Prepare the magnetic bead-antibody complex with 5 μg of the above-mentioned target antibody or control IgG and 50 μl of protein A/G magnetic beads, rotating at room temperature for 30 min. Take an equal volume of cell lysate and incubate the magnetic bead-antibody complex with rotation at 4 °C overnight so that the antibody can fully contact and bind to the protein. At the same time,10 μl cell lysate was extracted and used as input. Next day, RNA was extracted and purified with the prepared proteinase K buffer. Acquired RNA was used as a template to synthesize the corresponding cDNA.

Co-precipitated RNAs used for the first strand cDNA synthesis with the Reverse Transcription System (Roche) following the manufacturer’s protocol. Acquired cDNA was then analyzed by RT-qPCR. The information of IGF2BP1/2/3 antibody are ab184305, ab117809 and ab177477 (Abcam, Cambridge, MA, USA).

### Luciferase reporter assay

To evaluate the effect of 3′-UTR on HRAS expression, the wild type or mutant of 3′-UTR of HRAS was inserted behind the CDS region of the firefly luciferase (F-luc). Both vectors were transfected into wild type or METTL3-Mut/- PCa cells for 24 h, the firefly luciferase (F-luc) and Renilla luciferase (R-luc) were assayed by Dual-Glo Luciferase Assay system (Promega). Similarly, to evaluate the potential roles of 5′-UTR in MEK2 expression, the wild type or mutant 5′UTR ligated with promoter of MEK2 was inserted in the front of the F-luccoding region of the plasmid to generate MEK2-5′UTR, MEK2-5′UTR-Mut, respectively. Both plasmids were transfected into wild-type or METTL3-Mut/- PCa cells for 24 h. The Firefly luciferase (F-luc) and Renilla luciferase (R-luc) were assayed by Dual-Glo Luciferase Assay system (Promega). Specific steps of Dual-luciferase assay according to described protocol. Renilla luciferase (R-luc) was used to normalize Firefly luciferase (F-luc) activity. Experiments were carried out for three times to minimize the experimental bias.

### RNA stability

Stability of RNA in sh-con and sh-METTL3 cells was achieved by incubating cells with Actinomycin D (Act-D, Sigma, U.S.A) at 5 μg/ml. Cells were then collected at the indicated times and RNA was isolated for RT-qPCR.

### ASO design and usage

We used the online RNA-motif prediction tool (Sfold) to screen 20 bp ASO sequences specifically targeting METTL3 mRNA [27–30]. The mRNA sequence of METTL3 was obtained from NCBI. We selected 10 candidate ASOs including desired motifs [31] and modified them in the second generation “GAPMER” style, including phosphorothioate modification and 2’-O-MOE modification with 5 bases at both ends. ASOs for cell experiments were purchased from Sangon Biotech (Shanghai, China), while all ASOs for animal experiments were purchased from RiboBio (Guangzhou, China). All transfections in cells were performed by X-treme GENE HP Transfection Reagent (Roche). Two weeks after subcutaneous PCa cell injection, mice bearing C4-2^R^ tumors were randomly divided into ASO-con or ASO-2 groups and treated with intraperitoneal injections at 15 mg/kg daily for 1 day followed by 1 day off treatment for a total of 26 days. Mice were treated with enzalutamide (10 mg/kg) or vehicle from the 2nd week to the 6th week.

### Statistical analysis

For statistical analysis,the Student’s t-test and ANOVA tests were used to compare significant differences between the experimental and control groups. All analyses were performed using GraphPad Prism 8.0 (La Jolla, CA, USA) or SPSS 22 statistical software (SPSS, IBM Corporation, Armonk, NY, USA) and a two-tailed values of *p ≤ 0.05, **p ≤ 0.01, and ***p ≤ 0.001 were considered statistically significant. Data are reported as the mean ± SD from at least three independent experiments.

### Reporting summary

Further information on research design is available in the Nature Research Reporting Summary linked to this article.

## Supplementary information

S1

S-TABLE1-3

Supplementary Legends

Sample X Library QC

addtional file 1

Reporting Summary

Original Data File

## Data Availability

The MeRIP-seq data have been deposited in NCBI Gene Expression Omnibus database, and the accession number is GSE221961. The datasets generated and/or analysed during the current study are available from the corresponding author on reasonable request.
