# Supplementary material for: Post-transcriptional modification of m6A methylase METTL3 regulates ERK-induced androgen-deprived treatment resistance prostate cancer
Source: Cell Death Dis. 2023 Apr 24;14(4):289. doi: 10.1038/s41419-023-05773-5 (PMC10126012; doi:10.1038/s41419-023-05773-5)
Supplement: Supplementary file 3 — Supplementary Legends [file 41419_2023_5773_MOESM3_ESM.docx]

**Supplementary Legends**

**Fig.S1-S4**

S1.(**A**) The GO analysis showed the pathway of differential expression genes from RNA sequence.

S1.(**B**) The MeRIP-qPCR assay showed that the m^6^A level of HRAS and MEK2 in CRPC and CSPC.

S2.(**A**-**C**) The analysis of the IHC-stained tissue microarray showed that METTL14 (A), ALKBH5 (B) and FTO (C) protein levels in CSPC and CRPC.

S3.(**A-C**) There were significant differences in the levels of total m^6^A (A), RNA of METTL3 and METTL14 (B) and protein of METTL3, HRAS, MEK2 and p-ERK (C) between CSPC and CRPC cell lines (LNCap vs LNCap-AI).

S3.(**D**) After 22RV1 knockdown of METTL3, the RNA and protein levels of HRAS and MEK2 were detected by RT-qPCR and western blotting.

S3.(**E**) The mRNA levels of CCND1 and c-fos were detected in 4 cell lines (LNcap-AI and C4-2 transfected with sh-con, sh-METTL3, respectively) by qPCR.

S4. (**A-B**) MTT assay was used to detect the changes of cell viability before and after METTL3 knockdown in LNCap-AI and C4-2 cell lines. Cells were cultured with enzalutamide (20 nm).

S4. (**C**) An MTT assay was used to detect the changes of cell viability of LNCap oe-METTL3-mut and LNCap oe-METTL3 cell lines. Cells were cultured with enzalutamide (5 nm).

**S Table 1-3**

S Table 1. Primer sequence table.

S Table 2. shRNA and siRNA sequence table.

S Table 3. ASO sequence table.
