# Supplementary material for: Post-transcriptional modification of m6A methylase METTL3 regulates ERK-induced androgen-deprived treatment resistance prostate cancer
Source: Cell Death Dis. 2023 Apr 24;14(4):289. doi: 10.1038/s41419-023-05773-5 (PMC10126012; doi:10.1038/s41419-023-05773-5)
Supplement: Supplementary file 4 — Sample X Library QC [file 41419_2023_5773_MOESM4_ESM.pdf]

MeRIP-Seq QC Report

Quality Control (QC) of RNA

1. RNA Quantification and Quality Assurance by NanoDrop ND-1000

Sample ID	Sample Name	OD260/280 Ratio	Conc. (ng/μl)	Volume (μl)	Quantity (μg)	QC result
1	1-1	1.93	1153.21	100	115.32	Pass
2	1-2	1.89	1338.44	130	174.00	Pass
3	1-3	1.91	1362.76	150	204.41	Pass
4	2-1	1.93	1224.89	150	183.73	Pass
5	2-2	1.96	1103.76	150	165.56	Pass
6	2-3	1.93	1063.04	100	106.30	Pass
7	3-1	1.94	1190.93	150	178.64	Pass
8	3-2	1.93	1404.71	150	210.71	Pass
9	3-3	1.94	1565.08	150	234.76	Pass

2. RNA Integrity and gDNA contamination test by Denaturing Agarose Gel Electrophoresis

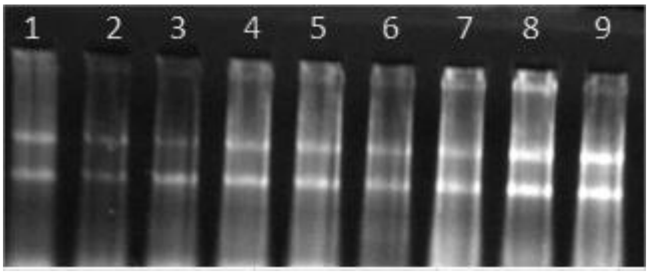

Lane 1: 1-1
Lane 2: 1-2
Lane 3: 1-3
Lane 4: 2-1
Lane 5: 2-2
Lane 6: 2-3
Lane 7: 3-1
Lane 8: 3-2
Lane 9: 3-3

Add: Building 71, Number 1066, North Qinzhou Road, Shanghai
Tel: 021-64878766
Web: http://www.cloud-seq.com.cn
Email: support@cloud-seq.com.cn

Quality Assessment of MeRIP

1. RNA Quantification and Quality Assurance by Qubit3.

	Sample ID	Sample Name	Concentration (ng/ul)	Volume (μl)	Quantity (μg)
MeRIP	1	1-1.IP	71.61	13	0.93
	2	1-2.IP	21.36	13	0.28
	3	1-3.IP	15.93	13	0.21
	4	2-1.IP	30.92	13	0.40
	5	2-2.IP	16.93	13	0.22
	6	2-3.IP	21.46	13	0.28
	7	3-1.IP	18.93	13	0.25
	8	3-2.IP	25.63	13	0.33
	9	3-3.IP	21.63	13	0.28
Input	10	1-1.Input	653.21	2	1.31
	11	1-2.Input	538.44	2	1.08
	12	1-3.Input	462.76	2	0.93
	13	2-1.Input	524.89	2	1.05
	14	2-2.Input	503.76	2	1.01
	15	2-3.Input	563.04	2	1.13
	16	3-1.Input	490.93	2	0.98
	17	3-2.Input	504.71	2	1.01
	18	3-3.Input	565.08	2	1.13

Library QC Report

Quality Assessment of Sequencing Library

Sequencing library was determined by Agilent 2100 Bioanalyzer using the Agilent DNA 1000 chip kit (Agilent, part # 5067-1504)

Sample Name	Size (bp)	Conc. (ng/μl)	Conc. (nmol/L)	Volume (μl)*	Total Amount (ng)
1-1.IP	278	6.80	37.1	10	68.0
1-2.IP	275	7.56	41.7	10	75.6
1-3.IP	278	4.25	23.2	10	42.5
2-1.IP	273	5.05	28.0	10	50.5
2-2.IP	274	7.01	38.8	10	70.1
2-3.IP	275	5.61	30.9	10	56.1
3-1.IP	274	7.70	42.6	10	77.0
3-2.IP	275	6.53	36.0	10	65.3
3-3.IP	275	7.88	43.5	10	78.8
1-1.Input	305	9.57	47.5	10	95.7
1-2.Input	298	12.99	66.0	10	129.9
1-3.Input	313	7.28	35.2	10	72.8
2-1.Input	299	5.63	28.6	10	56.3
2-2.Input	329	7.73	35.6	10	77.3
2-3.Input	301	5.31	26.7	10	53.1
3-1.Input	310	7.08	34.6	10	70.8
3-2.Input	306	5.81	28.8	10	58.1
3-3.Input	299	4.38	22.2	10	43.8

*The libraries were adjusted to 10nM before cluster generation.

Add: Building 71, Number 1066, North Qinzhou Road, Shanghai
Web: http://www.cloud-seq.com.cn
Tel: 021-64878766
Email: support@cloud-seq.com.cn

Sample 1-1.IP

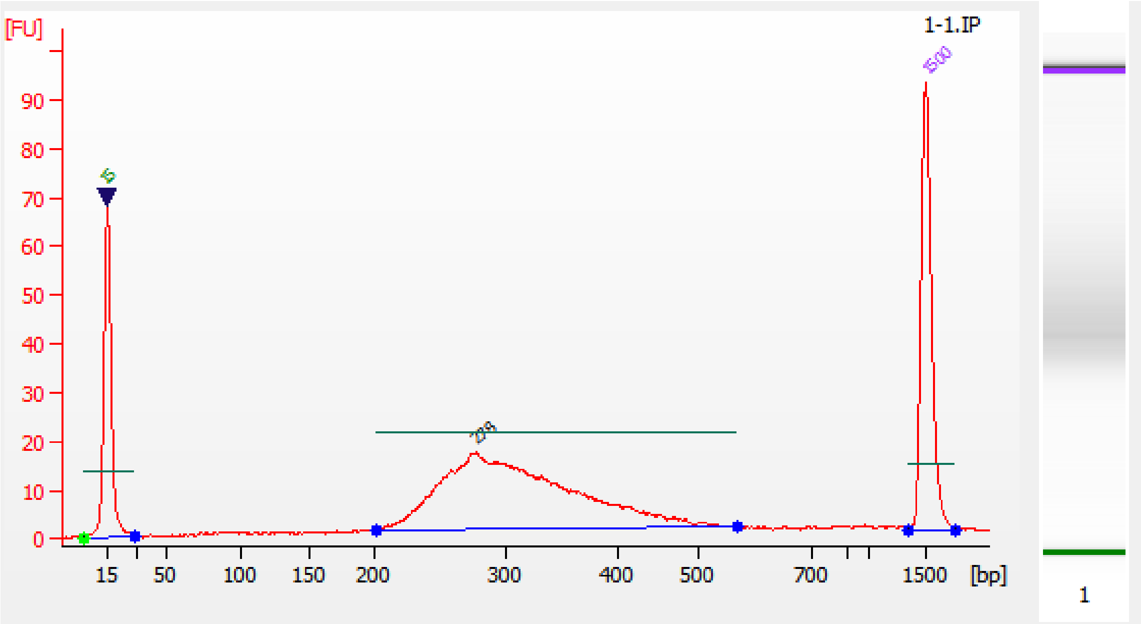

		Size [bp]	Conc. [ng/µl]	Molarity [nmol/l]	Observations
▶	◀	15	4.20	424.2	Lower Marker
2		278	6.80	37.1	
3	▶	1,500	2.10	2.1	Upper Marker

Sample 1-2.IP

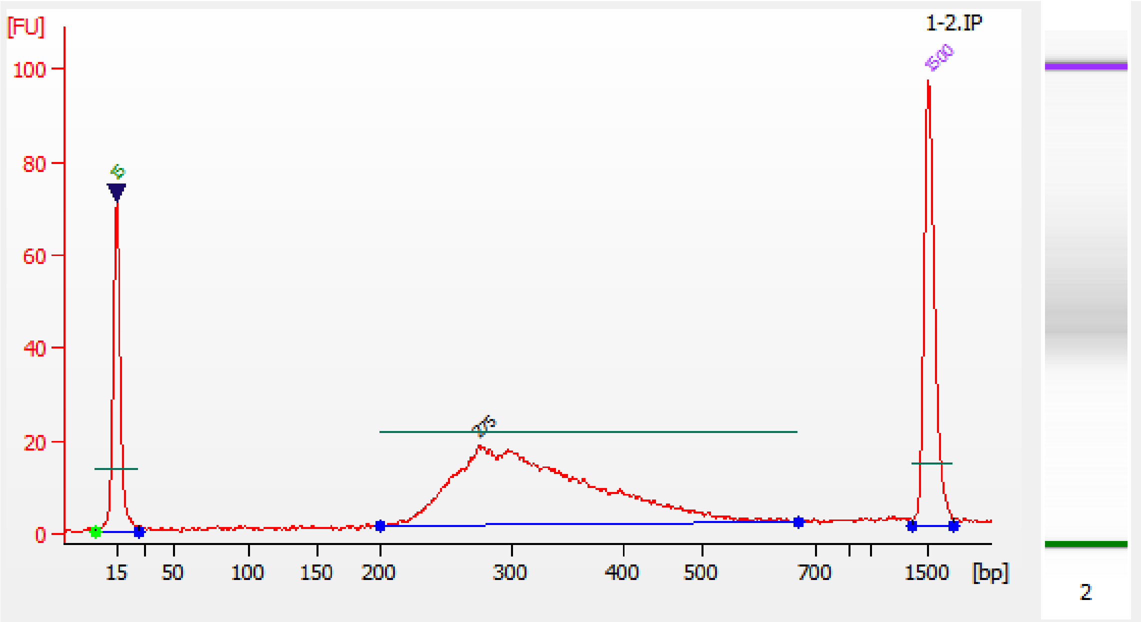

		Size [bp]	Conc. [ng/µl]	Molarity [nmol/l]	Observations
▶	◀	15	4.20	424.2	Lower Marker
2		275	7.56	41.7	
3	▶	1,500	2.10	2.1	Upper Marker

Add: Building 71, Number 1066, North Qinzhou Road, Shanghai
Tel: 021-64878766
Web: http://www.cloud-seq.com.cn
Email: support@cloud-seq.com.cn

Sample 1-3.IP

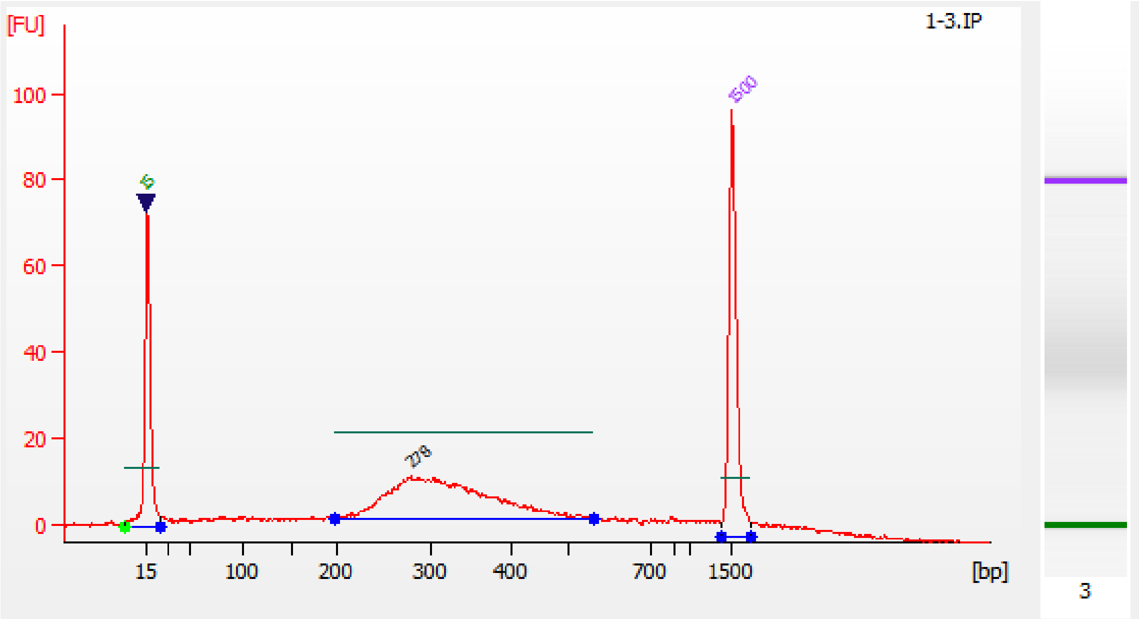

		Size [bp]	Conc. [ng/µl]	Molarity [nmol/l]	Observations	
▶	◀	15	4.20	424.2	Lower Marker	
2		278	4.25	23.2		
3	▷	1,500	2.10	2.1	Upper Marker	

Sample 2-1.IP

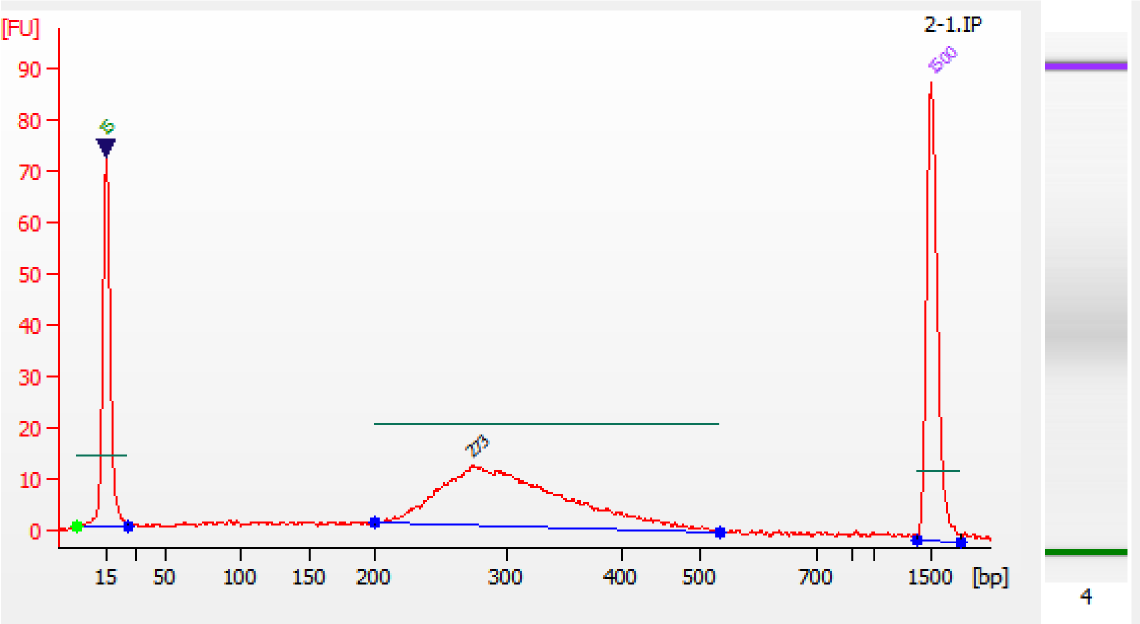

		Size [bp]	Conc. [ng/µl]	Molarity [nmol/l]	Observations	
▶	◀	15	4.20	424.2	Lower Marker	
2		273	5.05	28.0		
3	▷	1,500	2.10	2.1	Upper Marker	

Sample 2-2.IP

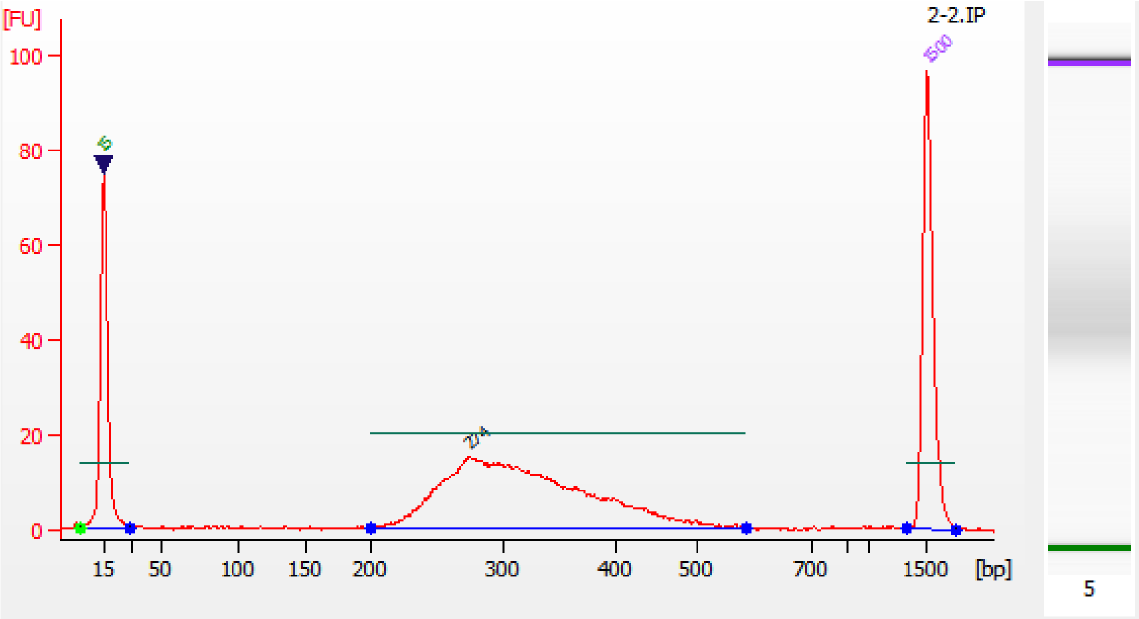

		Size [bp]	Conc. [ng/µl]	Molarity [nmol/l]	Observations
▶	◀	15	4.20	424.2	Lower Marker
2		274	7.01	38.8	
3	▶	1,500	2.10	2.1	Upper Marker

Sample 2-3.IP

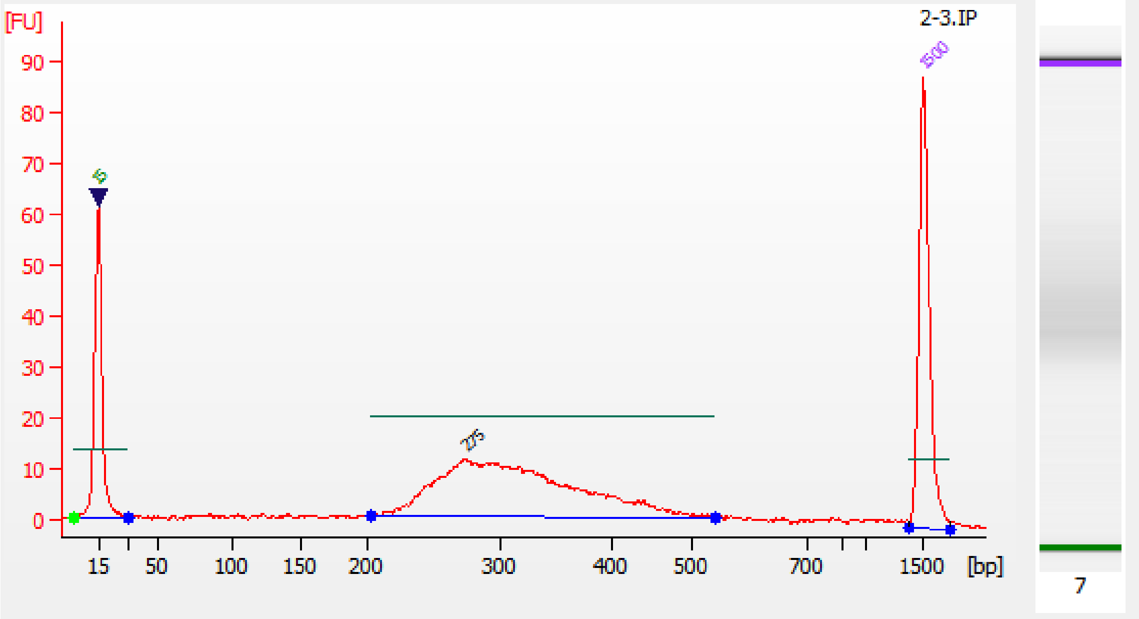

		Size [bp]	Conc. [ng/µl]	Molarity [nmol/l]	Observations
▶	◀	15	4.20	424.2	Lower Marker
2		275	5.61	30.9	
3	▶	1,500	2.10	2.1	Upper Marker

Sample 3-1.IP

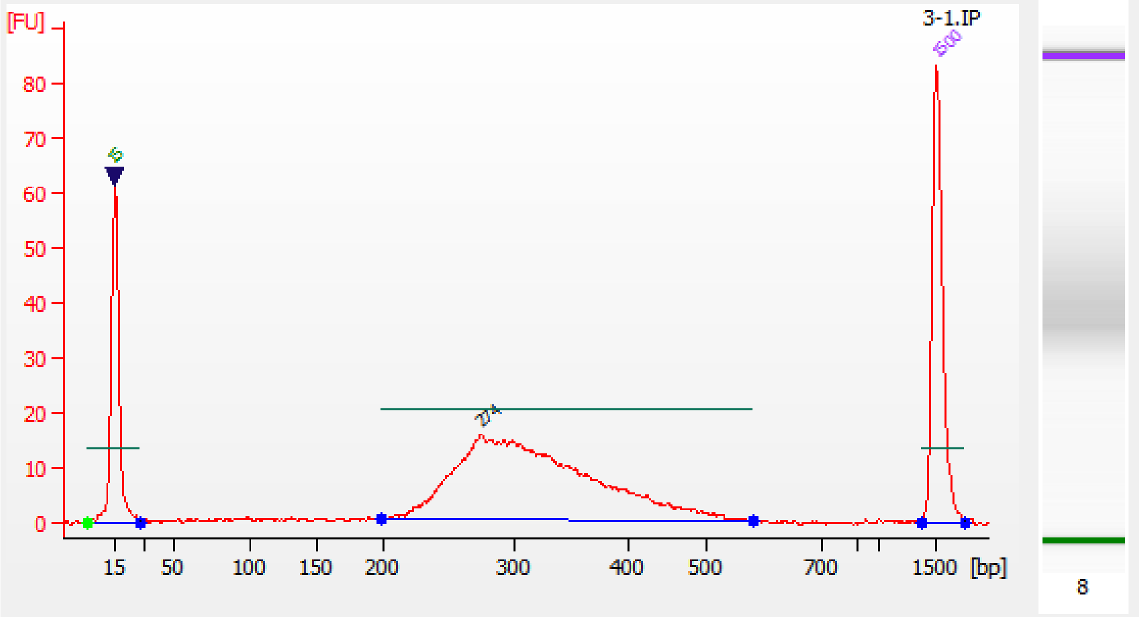

		Size [bp]	Conc. [ng/µl]	Molarity [nmol/l]	Observations	
▶	◀	15	4.20	424.2	Lower Marker	
2		274	7.70	42.6		
3	▶	1,500	2.10	2.1	Upper Marker	

Sample 3-2.IP

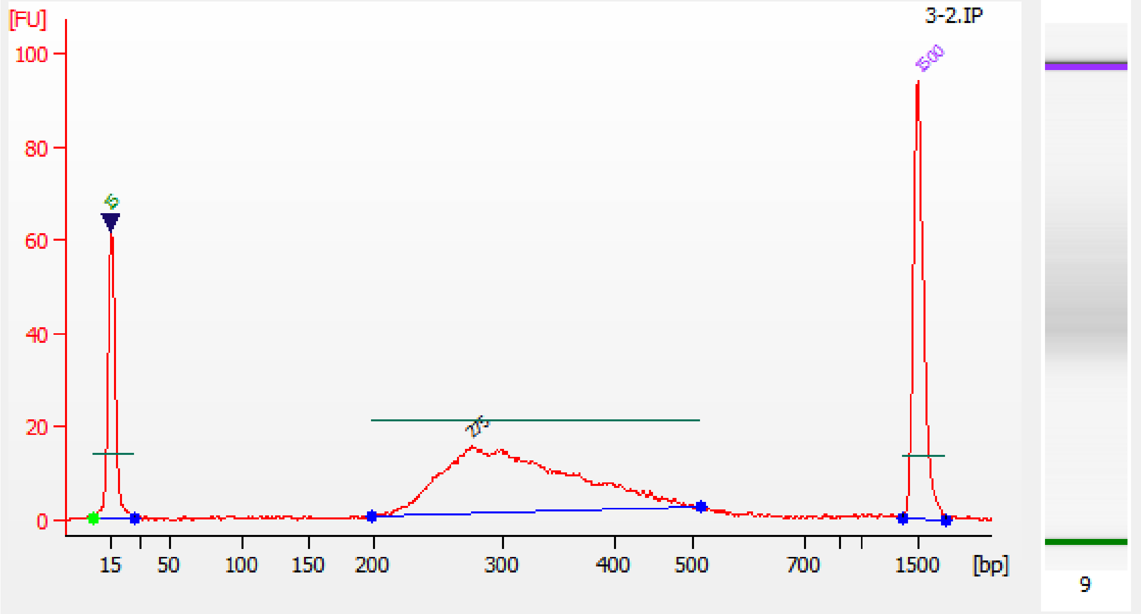

		Size [bp]	Conc. [ng/µl]	Molarity [nmol/l]	Observations	
▶	◀	15	4.20	424.2	Lower Marker	
2		275	6.53	36.0		
3	▶	1,500	2.10	2.1	Upper Marker	

Sample 3-3.IP

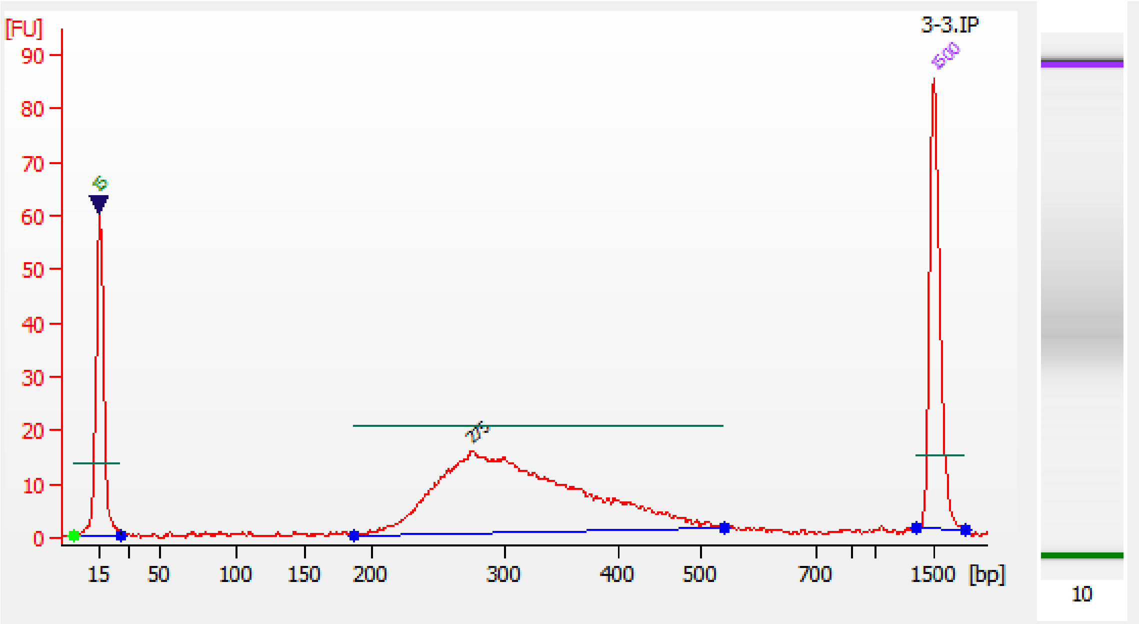

		Size [bp]	Conc. [ng/µl]	Molarity [nmol/l]	Observations
▶	◀	15	4.20	424.2	Lower Marker
2		275	7.88	43.5	
3	▶	1,500	2.10	2.1	Upper Marker

Sample 1-1.Input

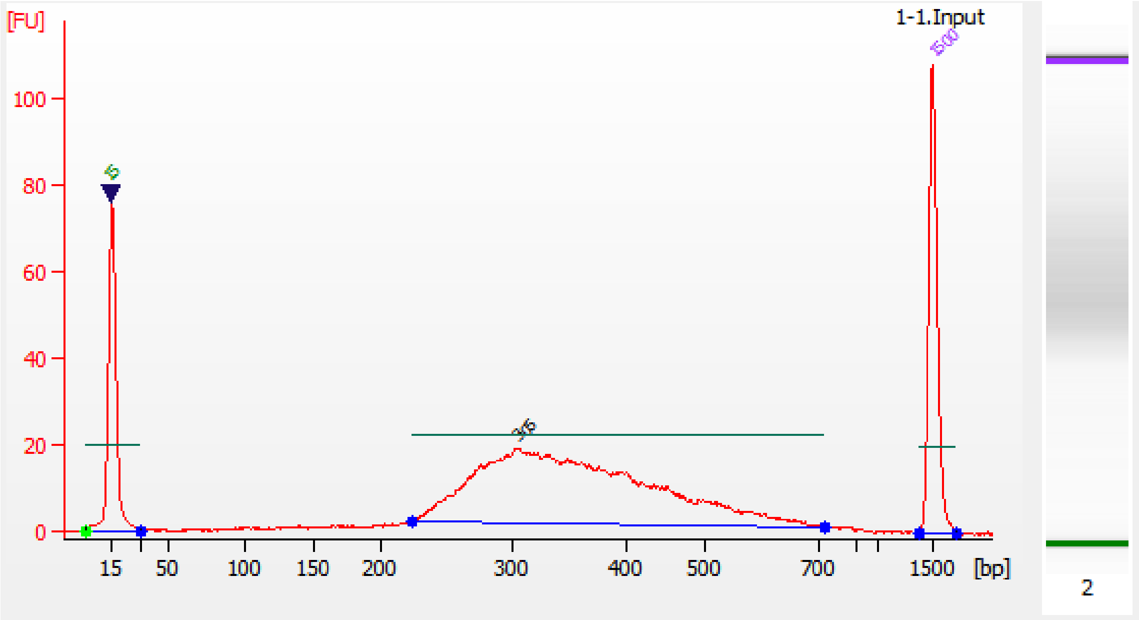

		Size [bp]	Conc. [ng/µl]	Molarity [nmol/l]	Observations
▶	◀	15	4.20	424.2	Lower Marker
2		305	9.57	47.5	
3	▶	1,500	2.10	2.1	Upper Marker

Add: Building 71, Number 1066, North Qinzhou Road, Shanghai
Tel: 021-64878766
Web: http://www.cloud-seq.com.cn
Email: support@cloud-seq.com.cn

Sample 1-2.Input

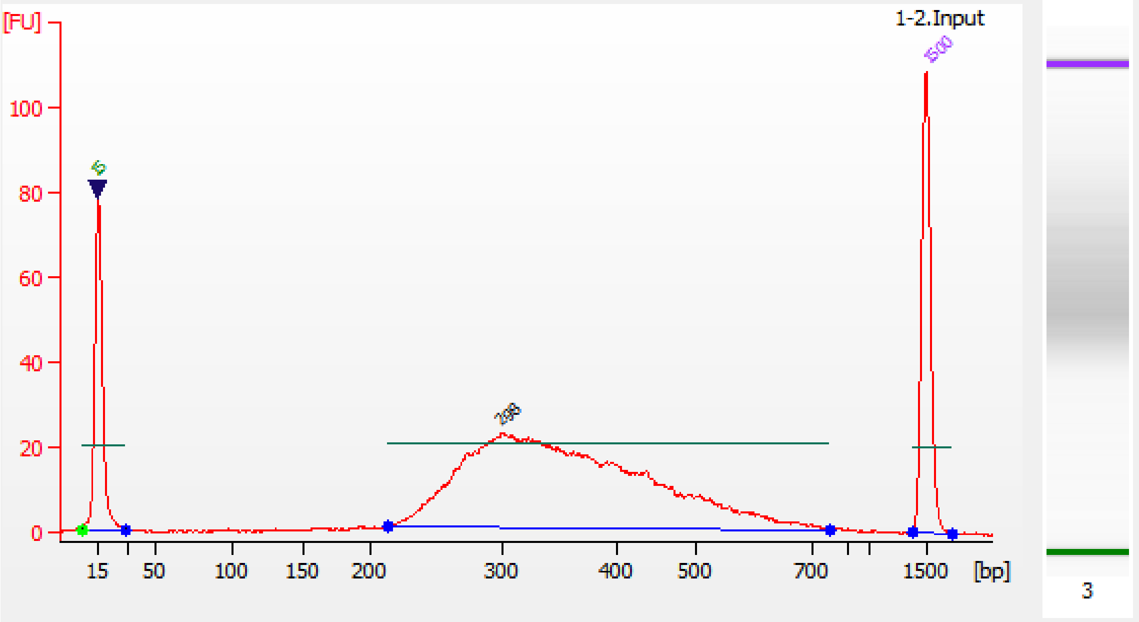

		Size [bp]	Conc. [ng/µl]	Molarity [nmol/l]	Observations
▶	◀	15	4.20	424.2	Lower Marker
2		298	12.99	66.0	
3	▷	1,500	2.10	2.1	Upper Marker

Sample 1-3.Input

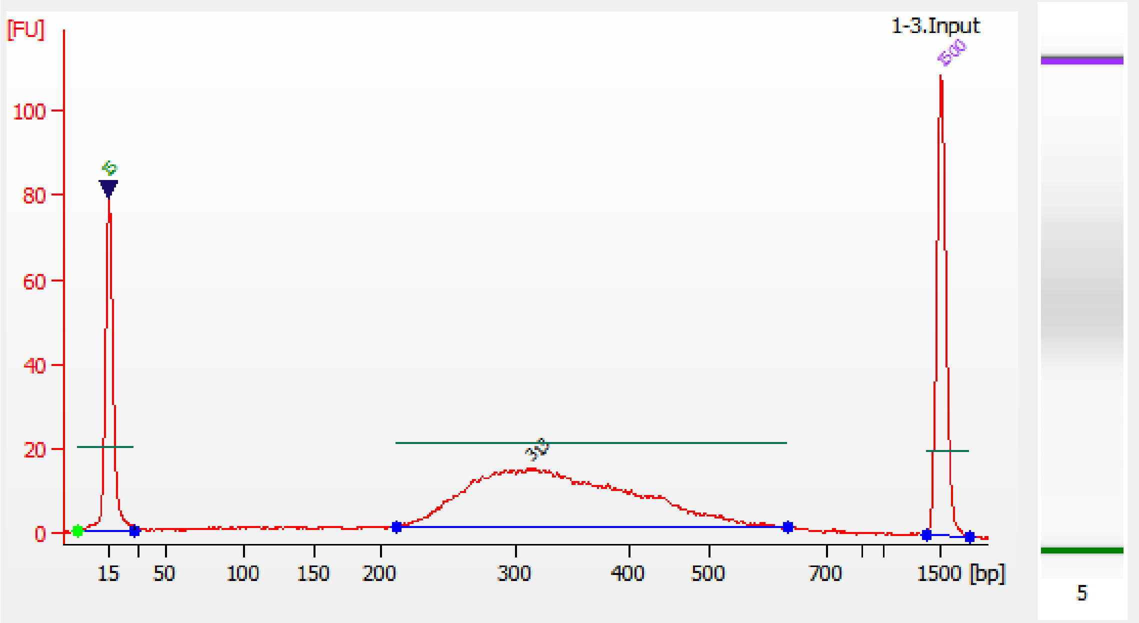

		Size [bp]	Conc. [ng/µl]	Molarity [nmol/l]	Observations
▶	◀	15	4.20	424.2	Lower Marker
2		313	7.28	35.2	
3	▷	1,500	2.10	2.1	Upper Marker

Sample 2-1.Input

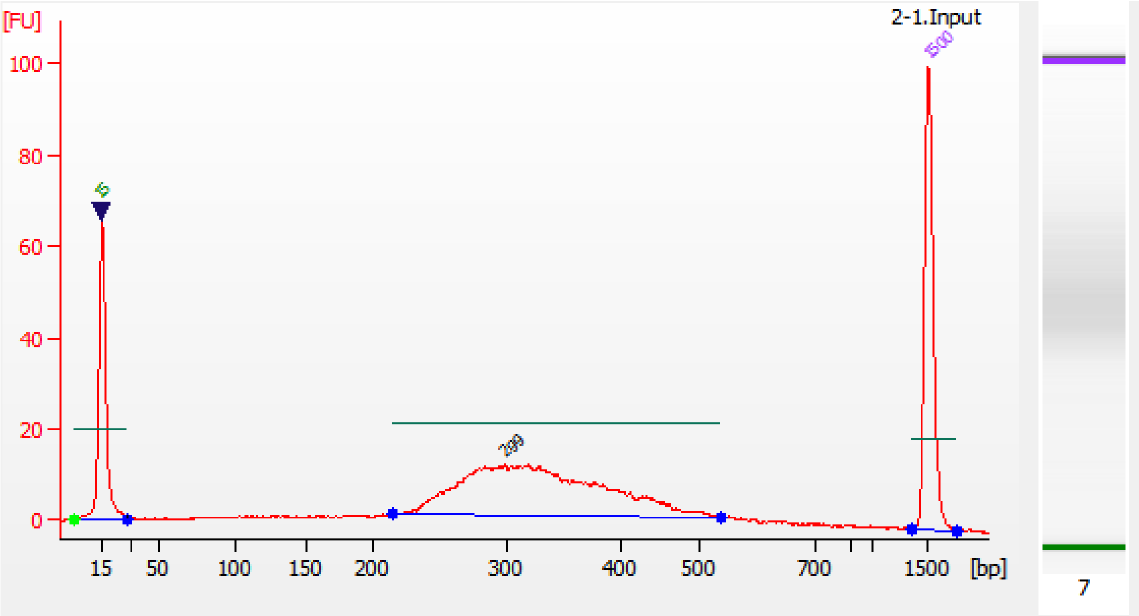

		Size [bp]	Conc. [ng/µl]	Molarity [nmol/l]	Observations
▶	◀	15	4.20	424.2	Lower Marker
2		299	5.63	28.6	
3	▶	1,500	2.10	2.1	Upper Marker

Sample 2-2.Input

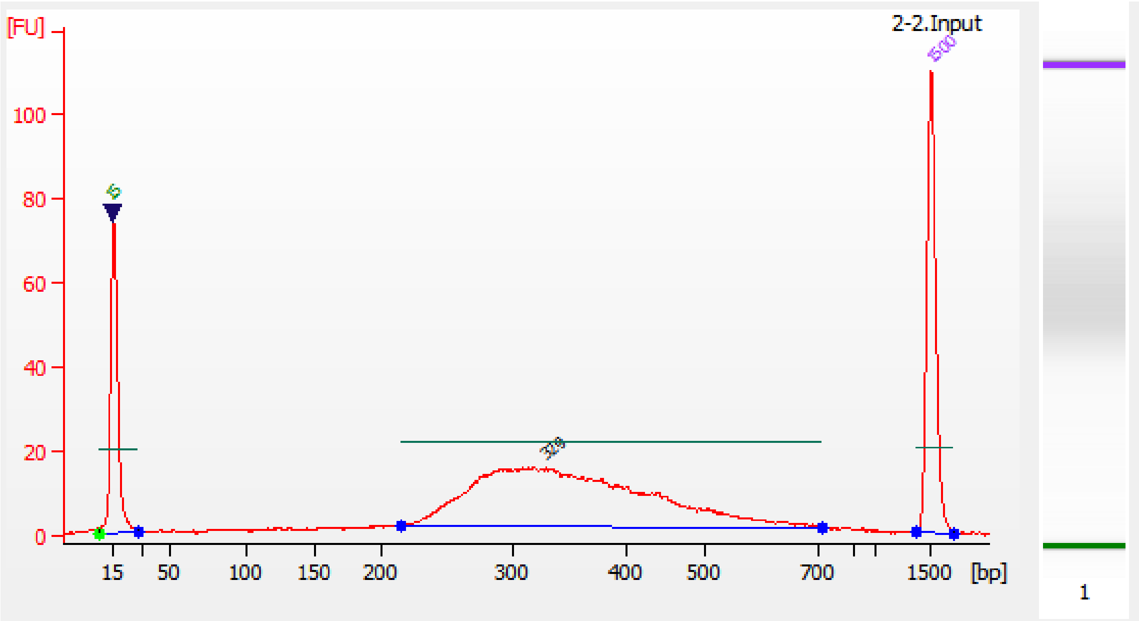

		Size [bp]	Conc. [ng/µl]	Molarity [nmol/l]	Observations
▶	◀	15	4.20	424.2	Lower Marker
2		329	7.73	35.6	
3	▶	1,500	2.10	2.1	Upper Marker

Sample 2-3.Input

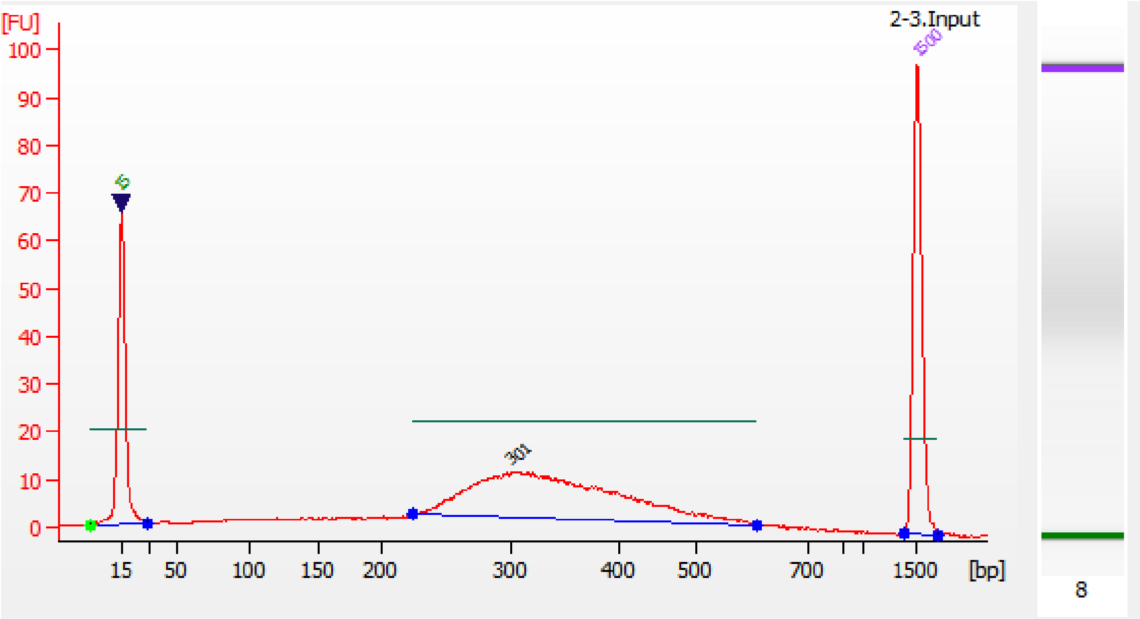

		Size [bp]	Conc. [ng/µl]	Molarity [nmol/l]	Observations	
▶	◀	15	4.20	424.2	Lower Marker	
2		301	5.31	26.7		
3	▶	1,500	2.10	2.1	Upper Marker	

Sample 3-1.Input

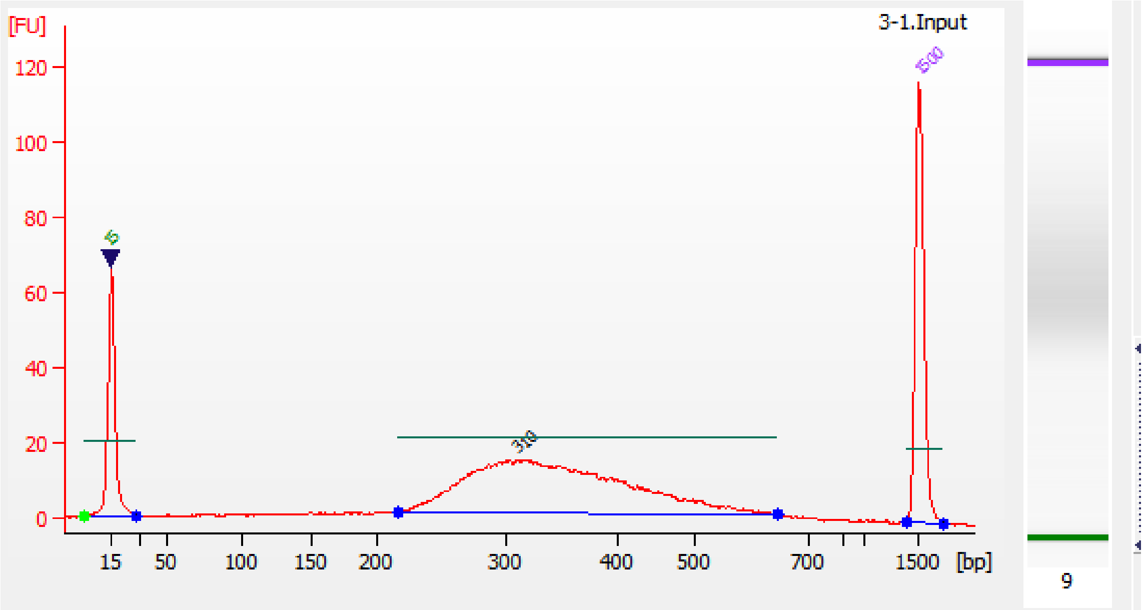

		Size [bp]	Conc. [ng/µl]	Molarity [nmol/l]	Observations	
▶	◀	15	4.20	424.2	Lower Marker	
2		310	7.08	34.6		
3	▶	1,500	2.10	2.1	Upper Marker	

Add: Building 71, Number 1066, North Qinzhou Road, Shanghai
Tel: 021-64878766
Web: http://www.cloud-seq.com.cn
Email: support@cloud-seq.com.cn

Sample 3-2.Input

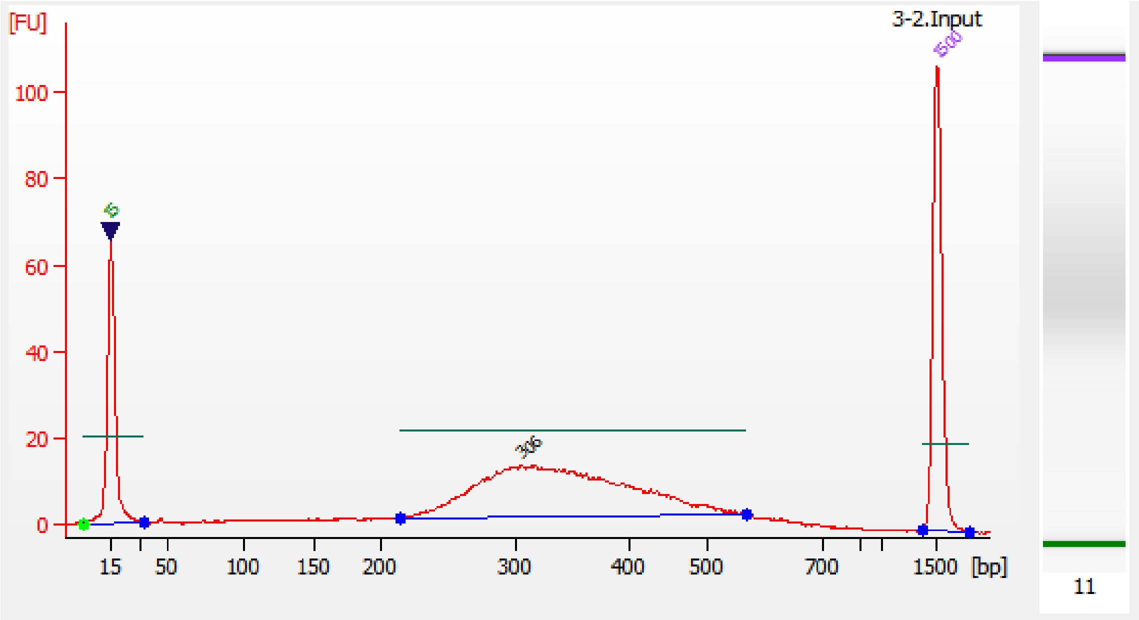

		Size [bp]	Conc. [ng/µl]	Molarity [nmol/l]	Observations	
		15	4.20	424.2	Lower Marker	
2		306	5.81	28.8		
3		1,500	2.10	2.1	Upper Marker	

Sample 3-3.Input

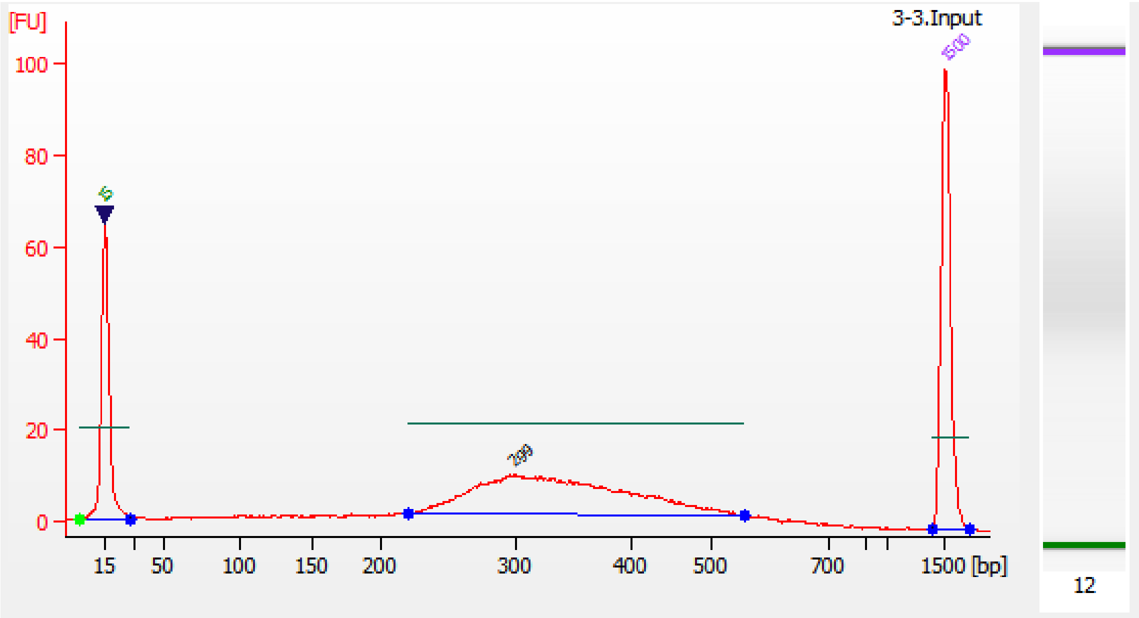

		Size [bp]	Conc. [ng/µl]	Molarity [nmol/l]	Observations	
		15	4.20	424.2	Lower Marker	
2		299	4.38	22.2		
3		1,500	2.10	2.1	Upper Marker	
