# Supplementary material for: Post-transcriptional modification of m6A methylase METTL3 regulates ERK-induced androgen-deprived treatment resistance prostate cancer
Source: Cell Death Dis. 2023 Apr 24;14(4):289. doi: 10.1038/s41419-023-05773-5 (PMC10126012; doi:10.1038/s41419-023-05773-5)
Supplement: Supplementary file 5 — addtional file 1 [file 41419_2023_5773_MOESM5_ESM.pdf]

分 类 号：R737.25　　　　　　　　　　　　　　　　学校代码：1 0 0 6 2
密　　级：　　　　　　　　　　　　　　　　　　　学　　号：20176020289

天津醫科大学
TIANJIN MEDICAL UNIVERSITY

硕 士 学 位 论 文

MASTER'S DISSERTATION

论文题目： METTL3 介导 RNA m6A 甲基化修饰影响激素敏感型前列腺癌的发生发展

TITLE　METTL3 promotes Growth of Hormone Sensitive Prostate Cancer by RNA N6-methyladenosine (m6A)

一级学科：临床医学

二级学科：外科学

泌尿外

论文作者：智超

导　　师：张传祥

天 津 医 科 大 学 研 究 生 院

二〇二〇年五月

分 类 号：R737.25 学校代码：1 0 0 6 2
密 级： 学 号：20176020289
学位类别： 科学学位☑ 专业学位☐ 学科门类：医学

天津醫科大学
TIANJIN MEDICAL UNIVERSITY

硕 士 学 位 论 文

MASTER'S DISSERTATION

论文题目：METTL3 介导 RNA m6A 甲基化修饰影响激素敏感型前列腺癌的发生发展

T I T L E METTL3 promotes Growth of Hormone Sensitive Prostate Cancer by RNA N6-methyladenosine (m6A)

一级学科：临床医学

二级学科：外科学

泌尿外

论文作者：智超

导 师：张传祥

导师组成员：牛远杰、尚芝群

天 津 医 科 大 学 研 究 生 院

二〇二〇年五月

学位论文原创性声明

本人郑重声明：所呈交的论文是我个人在导师指导下独立进行研究工作取得的研究成果。除了文中特别加以标注引用的内容和致谢的地方外，论文中不包含任何其他个人或集体已经发表或撰写过的研究成果，与我一同工作的同志对本研究所做的任何贡献均已在论文中作了明确的说明并表示了谢意。

学位论文作者签名：智超　　日期：2020 年 5 月 28 日

学位论文版权使用授权书

本学位论文作者完全了解天津医科大学有关保留、使用学位论文的规定，即：学校有权将学位论文的全部或部分内容编入有关数据库进行检索，并采用影印、缩印或扫描等复制手段保存、汇编以供查阅和借阅。同意学校向国家有关部门或机构送交论文，并编入有关数据库。

本论文属于

保密 □，在 ______ 年解密后适用本授权书。

不保密 ☑。

（请在相对应的方框内打"√"）

学位论文作者签名：智超　　日期：2020 年 5 月 28 日

导 师 签 名：张纯[illegible]　　日期：2020 年 5 月 28 日

中文摘要

目的: 应用体内体外实验检测前列腺癌中甲基转移酶 3（METTL3）表达水平。 探究 METTL3 在激素敏感型前列腺癌发生发展中的作用机制。

方法：应用免疫组织化学的方法分别检患者前列腺癌及良性前列腺组织 METTL3。利用 Image J 软件进行定量分析。通过体外细胞实验 Western Blot 及 qPCR 检测个前列腺癌细胞系及 BPH1 中 METTL3 含量对比。利用慢病毒转染 shRNA 构建稳定干扰 METTL3 的前列腺癌细胞系。利用 Western Blot 验证前列腺癌细胞敲低 METTL3 效率。通过体外细胞实验，MTT 以及细胞集落形成实验，验证 METTL3 对前列腺癌细胞的增殖能力的影响。利用高通量甲基化测序（m6A RNA-seq）分别对五名前列腺良性组织患者及前列腺癌患者组织进行测序。比较差异基因并通过信号通路富集分析，筛选通路。通过 Western Blot 及 qPCR 在细胞系中对富集差异基因表达进行验证。通过 RIP-qPCR 验证 METTL3 及 m6A 与目的基因 AKT,c-MYC ,YAP1 之间的关系及潜在机制。

结果：免疫组化证明与良性前列腺组织相比较，METTL3 在前列腺癌组织中主要呈高表达，差异具有统计学意义（$P<0.001$）。各前列腺癌细胞系中 METTL3 在 RNA 水平及蛋白水平均要高于 BPH1。2 种前列腺癌细胞系中下调 METTL3，可以显著降低前列腺癌细胞的增殖能力。下调 METTL3 前列腺癌细胞系，前列腺癌细胞中总体 RNA m6A 甲基化水平降低。高通量测序结果在激素敏感型前列腺癌组织中 m6A 的丰度改变。在 HSPC 中 Hippo 通路中的基因异常激活。METTL3 的敲低会导致 YAP1 及 c-MYC 的 mRNA m6a 水平的降低，并且致 YAP1，c-MYC 等相关基因表达下降。

结论：METTL3 在激素敏感型前列腺癌中异常表达，相比于前列腺良性病变组织有明显升高。METTL3 会影响 HSPC 细胞系的细胞增殖能力，敲低 METTL3 会减弱细胞增殖能力。在 HSPC 中 METTL3 介导了 m6A 的改变，进而影响了 Hippo-YAP 通路的异常激活，YAP1,c-MYC 等基因的异常表达。在前列腺癌细胞中 METTL3 的降低会使 YAP1、c-MYC、Cyclin D 等基因，m6A 水平明显的降低。从而导致其表达水平的下降。

关键词：激素敏感型前列腺癌（HSPC） METTL3 N6-甲基腺嘌呤甲基化修饰（m6A）c-MYC YAP1

Abstract

Objective: methyltransferase 3 (METTL3) was detected in prostate cancer in vitro and in vivo.The mechanism of METTL3 in the development of hormone-sensitive prostate cancer.

Methods: immunohistochemistry was used to detect prostate cancer and benign prostate tissue METTL3.Image J software was used for quantitative analysis.The contents of METTL3 in BPH1 and prostatic cancer cell lines were detected by Western Blot and qPCR in vitro.ShRNA transfected with lentivirus were used to construct stable prostate cancer cell lines that interfere with METTL3.Western Blot was used to verify the efficiency of knocking down METTL3 in prostate cancer cells.In vitro cell experiments, MTT and cell colony formation experiments were conducted to verify the effect of METTL3 on the proliferation of prostate cancer cells.High-throughput methylation sequencing (m6A RNA-seq) was used to sequence the tissues of five patients with benign prostate tissue and prostate cancer.The differentially expressed genes were compared and the signal pathways were enriched and screened.Western Blot and qPCR were used to verify the expression of enriched differential genes in cell lines.The relationship between METTL3 and m6A and target genes AKT and c-MYC YAP1 and its potential mechanism were verified by RIP-qPCR.

Results: compared with benign prostate tissue, immunohistochemistry showed that METTL3 was highly expressed in prostate cancer tissues, with statistically significant difference ($P<0.001$).The RNA level and protein level of METTL3 in each prostate cancer cell line were higher than that of BPH1.Down-regulation of METTL3 in two prostate cancer cell lines can significantly reduce the proliferation capacity of prostate cancer cells.METTL3 was down-regulated in prostate cancer cell lines, and the overall RNA m6A methylation level in prostate cancer cells was decreased.Changes in the abundance of m6A in hormone-sensitive prostate cancer tissues.Abnormal

activation of genes in the Hippo pathway in HSPC.The knockdown of METTL3 will lead to the decrease of m6a level of mRNA of YAP1 and c-MYC, and the decrease of expression of YAP1, c-MYC and other related genes.

Conclusion: METTL3 is abnormally expressed in hormone-sensitive prostate cancer and is significantly increased compared with benign prostate cancer.METTL3 will affect the cell proliferation ability of HSPC cell line, and knocking down METTL3 will weaken the cell proliferation ability.In HSPC, METTL3 mediates the change of m6A, which further affects the abnormal activation of Hippo-YAP pathway and the abnormal expression of YAP1, c-MYC and other genes.The reduction of METTL3 in prostate cancer cells will significantly reduce the level of YAP1, c-MYC, Cyclin D and other genes, and m6A.This leads to the decrease of its expression level.

Keywords: Hormone sensitive prostate cancer(HSPC) METTL3 N6-methyladenosine(m6A) c-MYC YAP1

目录

中文摘要…………………………………………………………………………Ⅰ
Abstract ……………………………………………………………………………Ⅱ
缩略语/符号说明 …………………………………………………………………Ⅵ
前言 ………………………………………………………………………………1
研究现状、成果……………………………………………………………………1
研究目的、方法……………………………………………………………………5
一、验证METTL3在良性前列腺组织和原发性激素敏感型前列腺癌组织及细胞中的表达异 ……………………………………………………………………6
1.1 对象和方法 ……………………………………………………………………6
1.1.1 研究对象 ……………………………………………………………………6
1.1.实验试剂及实验仪器 ………………………………………………………6
1.1.3 实验方法 ……………………………………………………………………12
1.2 结果 ……………………………………………………………………………20
1.2.1 前列腺癌组织与良性前列腺病变组织中METTL3蛋白表达有差异 ……………………………………………………………………………20
1.2.2 前列腺癌细胞系中METTL3在RNA水平表达高于良性前列腺病变细胞系 ……………………………………………………………………………22
1.2.3 前列腺癌细胞系中METTL3在蛋白水平水平表达高于良性前列腺病变细胞系 ……………………………………………………………………23
二、METTL3在前列腺癌细胞系中的增殖作用………………………………………26
2.1 对象和方法 ……………………………………………………………………26
2.1.1 研究对象 ……………………………………………………………………26
2.1.2 实验试剂及实验仪器 ………………………………………………………26
2.1.3 实验方法 ……………………………………………………………………30
1.2 结果 ……………………………………………………………………………33
1.2.1 慢病毒敲低前列腺癌中METTL3效率…………………………………………33
1.2.2 下调前列腺癌细胞METTL3表达，前列腺癌细胞的增殖能力受到影响。 ……………………………………………………………………………34
1.2.3 下调前列腺癌细胞METTL3表达，前列腺癌细胞的集落形成能力受到影响。 ……………………………………………………………………………35
2.3 讨论 ……………………………………………………………………………35
2.4 小结 ……………………………………………………………………………36
三、METTL3在前列腺癌中介导RNA m6A甲基化水平改变影响Hippo信号通路 ……………………………………………………………………………37
3.1 对象和方法 ……………………………………………………………………37
3.1.1 研究对象 ……………………………………………………………………37
3.1.2 实验试剂及仪器 ……………………………………………………………37
3.1.3 实验方法 ……………………………………………………………………41

3.2 结果 ……………………………………………………………………………45
3.2.1 敲低 METTL3 后细胞 RNA m6a 甲基化修饰水平改变 …………………45
3.2.2METTL3 的敲低会影响 c-MYC 的 mRNA 蛋白表达下降 …………………45
3.2.3METTL3 的敲低会影响 c-MYC 的 mRNA m6a 水平的降低导致其 mRNA 表达水平改变 ……………………………………………………………………………47
3.3 讨论 ……………………………………………………………………………48
3.4 小结 ……………………………………………………………………………50
结论………………………………………………………………………………………51
参考文献…………………………………………………………………………………52
附录………………………………………………………………………………………55
综述………………………………………………………………………………………56
RNA N6-甲基腺苷（m6A）修饰在生物体中作用以及其在肿瘤中的研究 ……56
综述参考文献 …………………………………………………………………………65
致谢………………………………………………………………………………………71
个人简历…………………………………………………………………………………72

缩略语/符号说明

英文缩写	英文全称	中文全称
PCa	Prostate Cancer	前列腺癌
HSPC	Hormone sensitive prostate cancer	激素敏感型前列腺癌
m6A	N6-methyladenosine	N6-甲基腺嘌呤
METTL3	methyltransferase like 3	甲基转移酶 3
YAP1	yes-associated protein 1	yes 相关蛋白 1
c-MYC	MYC proto-oncogene	MYC 原癌基因
DMSO	Dimethyl sulphoxide	二甲基亚砜
FBS	Fetal bovine serum	胎牛血清
PBS	Phosphate buffer saline	磷酸盐缓冲液
PCR	Polymerase chain reaction	聚合酶链反应
qPCR	Quantitative real-time PCR	实时定量聚合酶链反应
RT-PCR	Reverse transcription PCR	反转录聚合酶链反应
cDNA	complementary DNA	互补 DNA
TBS	triethanolamine buffered saline solution	三乙醇胺缓冲盐水溶液
MeRIP	RNA N6-methyladenosine Binding Protein immunoprecipitation	RNA 甲基化结合蛋白免疫沉淀）

前言

研究现状、成果

如今癌症已成为世界卫生最大问题之一。前列腺癌(Prostate Caner)在全世界范围内发病率已经跃居第二，在男性肿瘤中发病率是众多肿瘤之首[1-2]，是全球范围内发病率最高的泌尿系癌症，也已成为男性的第一大癌症。此外，欧美发达国家的前列腺癌发病率高于发展中国家，男性肿瘤发病率居首位，严重危害男性健康。中国是一个前列腺癌发病率较低的国家[3]。但近年来，随着社会发展医疗水平提高，平均寿命增加，以及一些环境污染、不良生活习惯、长期压力等问题，我国前列腺癌发病率呈现逐年上升，在新增癌症人数占比中逐渐增加[4]。与发达国家近年来前列腺癌检出率下降有所不同。

我国前列腺癌患者在发现确诊时多为原发性激素敏感型前列腺癌（HSPC）。对于导致前列腺癌的危险因素及其机制尚未非常明确。已经通过大范围动的研究被 确认的因素有年龄，种族和遗传性[4]。随着男性年龄的增加，患有前列腺癌的几率是逐年增加。在我国前列腺癌患者主要是老年男性，患者中位年龄为 72 岁，大多患者年龄集中在 75~79 岁[5]。而种族因素体现，亚洲男性患前列腺癌比例要小于西方国家白种人的患病率。我国于 2009 年前列腺癌患病率在 9.921/10 万[6]。前列腺癌具有遗传因素。如果一级亲属患有前列腺癌，其本人患前列腺癌的危险性会增加一倍以上。此外认为还有外源性因素可能引起前列腺癌发生发展，比如维生素 E 、异黄酮的摄入不足，亦或者阳光照射等。对于这些暂未定论。

随着人类科学的发展，医学已经进展到了基因组学以及精准医学。各种基因被研究发现其在恶性肿瘤的发生、发展中起到的作用以及角色。而除了基因本身的改变，基因因为外界或者修饰导致的表达水平以及作用改变逐渐被人类知晓。表观遗传学是一门新兴的学科，研究再不涉及细胞核 DNA 序列的同时影响基因的作用的学科[7]。在表观遗传学中，这种改变通常是可逆遗传表型。他的研究对象包括 DNA 和 RNA 甲基化、组蛋白修饰、非编码 RNA 修饰和染色质重排等。这些动态修饰影响着基因表达、蛋白功能等关键的生物学过程，在生物生长发育、衰老、疾病发生等方面起着重要的调控作用。在早期 DNA 甲基化和组蛋白修饰研究较多也比较深。比如 DNA 中的 5-甲基胞嘧啶甲基化已被证明

影响许多疾病的基因表达而导致癌症[8]。而在 RNA 水平上，有着类似 DNA 甲基化的表观遗传修饰。至今为止已经发现非常多的 RNA 修饰。

这些 RNA 修饰普遍存在于真核生物中，极大地丰富了 RNA 的功能，协助 RNA 遗传信息的转换行使其功能。RNA 有着重要的生物学功能，RNA 甲基化是 RNA 修饰的主要形式，广泛存在于各种 RNA 中，如核糖体 RNA (rRNA)、转移 RNA (tRNA)、小核 RNA (snRNA)、小核仁 RNA (snoRNA)、mRNA 等[9-13]。mRNA 是 DNA 与蛋白质之间的核心分子连接，是基因信息读取过程的一部分，其化学修饰是目前研究最多的领域。mRNA 的修饰有 n7 -甲基鸟嘌呤(m7G)、n6 -甲基腺嘌呤(m6A)、5-甲基胞嘧啶(m5C)、n1 -甲基腺嘌呤(m1A)等。其中 m6A 修饰约占 mRNA 修饰的 80%，虽然 m6a 在 20 世纪 70 年代就被发现[14]，但因为生物技术的进步，基于抗体的免疫沉淀和高通量测序，m6a 的转录组水平的分析才成为可能，直到近年得到了广泛关注和研究，对其功能的展开详细的研究。

METTL3 是哺乳动物细胞中 m6A 甲基化转移酶复合物。m6A mRNA 甲基化由多组分甲基催化转移酶复合物，从 HeLa 细胞的核中分离出来。METTL3（MT-A70）是一种 70 kDa 的蛋白质[15]。 METTL3 在人体组织中含量非常丰富，尤其是在睾丸中，并且在多个物种中都是保守的,从酵母到人的真核生物中具有高度保守性。在 HeLa 细胞中敲低 METTL3 的表达，总 m6A 水平下降了约 30%。在 HepG2 细胞中同样敲低 METTL3，发现诱导了细胞凋亡[16,17]。m6A 甲基转移酶复合物有着 s -腺苷蛋氨酸(SAM)这一亚基机构，作为甲基供体，使腺嘌呤 6 号位上的 N 甲基化。METTL3 对 SAM 有着结合位点，催化 m6A 合成。在之前报道中，通过免疫荧光实验定位了 METTL3 主要分布在核散斑上，是 RNA 加工的主要不为，说明 m6A 在 RNA 加工过程中具有显著的调控作用[15,19]。在另一实验发现带有重组标记的人 METTL3 蛋白自身的活性较低，在体外获得最佳活性需要其他成分。

之后通过生物信息学的分析以及基因组学的鉴定发现 METTL14 和 METTL3 接近,具有保守基序的 METTL3 的同源物含有 Asp-Pro-Pro-Trp 或 Glu-Pro-Pro-Leu41。科学家发现在 HeLa 和 293FT 细胞中敲低 METTL14，不敲低 METTL3，也会导致 RNA m6A 水平降低[18]。生化表征透露这两种蛋白质形成稳定的复合物化学计量比为 1：1。因此，MELLT14 被认为是 m6A 甲基转移酶复合物的另一个亚基。在 m6A 合成过程中，METTL14 和 METTL3 以 1:1 的比例形成稳定的异质二聚体[15]。

另外还有 WTAP，三者形成稳定的 m6A 甲基转移酶复合物具有酶活性高、底物选择性强等特点[20]。与 m6A 的保守序列一致，复合物主要倾向于甲基化[G/A][G/A]AC[A/C/U]序列位点，对 RNA 底物的二级结构没有严格要求。METTL14 的缺失可导致 HeLa 和 HEK93 细胞 mRNA 的 m6A 的减少。WTAP 的缺失影响复合物的稳定性。

以上 METTL3，METTL14 以及 WTAP 被在 m6A 修饰中被称之为书写蛋白（Writter）随着 FTO 的发现其在 m6A 修饰中具有去甲基化的作用，奠定了 m6A 修饰的可逆性。FTO 以及 ALKBH5 在 m6A 修饰中被称为擦除蛋白（Erasers）[21,22]。还有发挥 m6A 修饰作用的甲基化识别蛋白（Reader），主要包括 YTHDF、YTHDC 家族几种相关的蛋白质，他们能够识别 RNA 上的 m6A 修饰，通过结合招募相关蛋白使 m6A 修饰发挥其功能[23-27]。

随着 RNA 表观遗传学的发展，m6A 在肿瘤的作用逐渐成为研究热点。近年来，RNA 甲基化特异性免疫沉淀高通量测序技术（meRIP-seq）的发展，METTL3 和 METTL14 被报道在肺癌、脑瘤、白血病等癌症中发挥重要作用。2016 年的研究报道，LIN S 利用 meRIP-seq 技术检测到肺癌 A549 细胞系的靶基因 9298 个 m6A 峰。EGFR、TAZ、DNMT3A 的癌基因的 m6A 广泛分布在停止密码子周围。METTL3 基因敲低导致 EGFR、TAZ 和 DNMT3A 基因下调。METLL3 通过与细胞质中的翻译起始复合物 eIF3b 相互作用，促进 m6A 含量丰富的 mRNA 癌基因的翻译。增加 METTL3 的表达可促进肺癌细胞的增殖、生长和侵袭[28]。2017 年，DuM 实验报道报道 METTL3 在非小细胞肺癌（NSCLC）组织中的表达高于癌旁组织。METTL3 在 NSCLC 组织中的表达与 miR-33a 的表达呈正相关。MiR-33a 能够在 mRNA 和蛋白水平上降低 METTL3 的表达，揭示了 miRNA 调控 METTL3 的新机制[29]。

另外在胶质母细胞瘤中，METTL3 起到了抑癌作用，在体外实验中发现下调胶质母细胞瘤细胞中 METTL3 或 METTL14 的表达水平，会使其增殖和自我更新能力得到增强。而过表达 METTL3 则抑制肿瘤细胞的生长[15]。

m6A 在白血病的发生发展中也起到了的作用，Vu 研究团队通过三名急性髓系白血病(AML)患者发现，METTL3 在 AML 细胞和原发性白血病细胞中高度表达。之后运用体外实验，下调 AML 细胞中 METTL3 的水平后，m6A 水平随之降低，而细胞分化和凋亡的能力得到增强，集落形成能力下降。m6A 交联和免疫沉淀反应(miCLIP)和 RNA-seq 实验显示，METTL3 介导 m6A 水平能够改变了

目标基因的 mRNA 的表达水平。在 AML 细胞中，METTL3 高表达，使得原癌基因 BCL2 的 mRNA 中 m6A 水平增高，增强其翻译，从而导致 BCL2 的表达水平增加，并且使得 AML 细胞处于低分化。[30]。

m6A 在肝细胞癌（HCC）组织中的表达低于癌旁非肿瘤性组织和正常组织。在 HCC 患者中，METTL14 的下调预后较差。在转移性肿瘤或门静脉肿瘤血栓中发现 METTL14 mRNA 表达非常低，提示 METTL14 可抑制肝癌细胞转移。共免疫沉淀实验表明，干预增加 METTL14 表达导致成熟 miR126 和 pri-miR126 与 DGCR8 结合水平升高，显示 METTL14 通过影响 m6A 表达水平的方式升高 miR126 水平来抑制 HCC 转移[31]。

最近的研究在子宫颈癌细胞系 SiHa 中，利用通过 shRNA 敲除 METTL3，METTL14 或者过表达 FTO，ALKBH5，会影响细胞 m6A 水平的降低，从而使得肿瘤的增殖能力增强，有助于肿瘤的生长。相反，当 m6A 水平增高时，宫颈癌细胞的生长受到了抑制，提示 METTL3 和 METTL14 在宫颈癌中具有抑癌作用[32]。而在肾细胞癌中也有类似研究研究。研究发现在肾细胞癌患者中，高表达 METTL3 的患者相对于低表达患者预后良好，提示 METTL3 可能在肾细胞癌细胞增殖、侵袭性和转移过程中起抑癌基因的作用。METTL3 可能通过调节上皮-间质转化和 PI3K-Akt-mTOR 通路发挥作用[33]。但在前列腺癌中尚未有具体详尽的机制研究报道。

研究目的、方法

研究目的：基于学习了解关于表观遗传学 m6A 修饰以及 RNA 甲基化酶 METTL3 的文献的基础之上，本研究旨在通过实验验证人患者 HSPC 与良性前列腺组织病变组织中 METTL3 表达水平的差异。在此基础上，通过体外细胞实验探索其对前列腺癌细胞增殖等生物学功能的影响，并且同时通过 MeRIP-seq 实验技术对于 HSPC 与良性前列腺组织病变组织中 m6A 修饰水平差异，寻找其潜在机制。目的研究 HSPC 中 METTL3 及表观遗传学 m6A 起到的作用，期望能够提供一种新的诊疗方式。

研究方法：在我们研究中，通过我们收集了前列腺癌患者组织标本，及良性前列腺病变的组织标本。前列腺癌标本均来自于首次检测出前列腺癌患者，

在行前列腺癌根治性切除术前并未接受过手术或者药物趋势治疗、药物抗雄激素治疗、放射治疗、化学治疗等治疗，均为激素敏感性前列腺癌患者。而良性前列腺病变组织来自于癌旁组织及前列腺增生组织。通过免疫组织化学染色实验，检测组织中 METTL3 表达水平。利用 Image J 软件进行半定量的评分，通过统计学验证两组之间差异。同时我们利用前列腺癌细胞系以及良性前列腺增生细胞系通过体外实验印证组织免疫组化实验结果。利用蛋白电泳免疫印迹实验（Western Blot）以及实时荧光定量核酸扩增检测系统（Real-time Quantitative PCR Detecting System qPCR）检验细胞中 METTL3 蛋白水平表达量以及 RNA 水平表达量差异。之后我们选取细胞系通过慢病毒 shRNA 敲减前列腺癌细胞系中的 METTL3，建立了稳定敲低 METTL3 的前列腺癌细胞系，通过体外细胞实验检验细胞生物功能。选取收集患者的组织进行 RNA 水平高通量测序和 m6A RNA 测序，通过生物信息学分析寻找 METTL3 在前列腺癌中发挥的潜在机制。并利用建立的稳定敲低 METTL3 前列腺癌细胞通过 Western Blot 以及 MeRIP-qPCR 进行验证。

一、验证 METTL3 在良性前列腺组织和原发性激素敏感型前列腺癌组织及细胞中的表达差异

通过运用免疫组化实验检测 METTL3 在患者原发性激素敏感型前列腺癌组织中与良性前列腺病变组织中的表达量；通过运用 q-PCR 和 Western Blot 实验检测 METTL3 在良性前列腺细胞系与各前列腺癌细胞系中的表达量；

1.1 对象和方法

1.1.1 研究对象

1.1.1.1 人前列腺组织石蜡标本：收集我院在 2016 年 1 月到 2017 年 12 月期间收治前列腺疾病患者。随机选取样本保存较好的患者组织标本，其中良性前列腺病变标本 18 例，前列腺癌组织标本 21 例。其中 18 例良性前列腺病变标本依据天津医科大学第二医院病理科报告为评判标准，21 例前列腺癌组织标本来自患者均为原发性前列腺肿瘤，标本为首次前列腺癌根治性切除手术术后取得标本，手术前没有经过其他治疗，包括手术或者药物趋势治疗、药物抗雄激素治疗、放射治疗、化学治疗等，均为激素敏感性前列腺癌患者。手术标本制备由我院病理科包埋制作并保存。

1.1.1.2 良性前列腺增生细胞系及前列腺癌细胞

BPH1 人良性前列腺增生细胞系、LNCaP 人前列腺 癌细胞系、22-RV1 人前列腺癌细胞系、PC3 人前列腺癌细胞系、DU145 人前列腺癌细胞系、C4-2 人前列腺癌细胞系，来自于美国组织培养中心（ATCC），及 LNCaP-AI 前列腺癌细胞系，由天津市泌尿外科研究所去雄培养建立的前列腺癌细胞模型。

1.1.2 实验试剂及实验仪器

1.1.2.1 实验试剂

名称	来源
二甲苯	苏州海百化工有限责任公司
无水乙醇	苏州海百化工有限责任公司
磷酸盐缓冲液	北京索莱宝科技有限公司

枸橼酸盐缓冲液	北京索莱宝科技有限公司
去离子水	天津市泌尿外科研究所
3%过氧化氢溶液	北京中杉金桥生物科技技术有限公司
免疫组化即用型 SP 通用试剂盒	北京中杉金桥生物科技技术有限公司
兔抗人 METTL3 单克隆抗体(10μL)	abcam corporation
山羊抗兔二抗（100μL）	北京中杉金桥生物科技技术有限公司
DAB dying kit	北京索莱宝科技有限公司
苏木素	天津市化学试剂一厂
中性树胶	天津市化学试剂一厂
RPMI Media 1640 细胞培养基	美国 Gibco 公司
Gibco 热灭活胎牛血清（FBS）	美国 Gibco 公司
去除雄激素热灭活胎牛血清（CD-FBS）	美国 Gibco 公司
Penicillin-Streptomycin 双抗	美国 Gibco 公司
Trypsin-EDTA (0.05%)（胰酶）	美国 Gibco 公司
二甲基亚砜（DMSO）	北京索莱宝科技有限公司
磷酸盐缓冲液（PBS）	北京索莱宝科技有限公司
Trizol	美国 Invitrogen 公司
三氯甲烷（氯仿）	天津市光复科技发展有限公司
异丙醇	天津市光复科技发展有限公司
无水乙醇	苏州海百化工有限责任公司

RIPA 裂解液	美国 Thermo Fisher Scientific 公司
PMSF 蛋白酶抑制剂	美国 Thermo Fisher Scientific 公司
Bradford(考马斯亮蓝)缓冲液	美国伯乐公司
Loading buffer 缓冲液	北京索来宝试剂有限公司
Western-blot 蛋白 marker	北京索来宝试剂有限公司
SDS-PAGE 凝胶制备试剂盒	北京索来宝试剂有限公司
30%Acr/Bis(29:1)	北京索来宝试剂有限公司
1M Tris-HCl(PH6.8)	北京索来宝试剂有限公司
1.5 Tris-HCl(PH8.8)	北京索来宝试剂有限公司
10%SDS	北京索来宝试剂有限公司
10%PAGE 胶凝固剂	北京索来宝试剂有限公司
PAGE 胶促凝剂	北京索来宝试剂有限公司
ddH_2O	天津市泌尿外科研究所
甘氨酸	美国 Sigma 公司
三羟甲基氨基甲烷（Tris）	美国 Sigma 公司
Tween-20	美国 Sigma 公司
十二烷基磺酸钠	美国 Sigma 公司
脱脂奶粉	美国 Sigma 公司
RevertAid First Strand cDNA Synthesis Kit	美国 Thermo Fisher Scientific 公司

Taq MasterMix	美国 Thermo Fisher Scientific 公司
兔抗人 METTL3 单克隆抗体	abcam corporation
兔抗人 m6A 单克隆抗体	德国默克集团
鼠抗人 GAPDH 单克隆抗体	北京中杉金桥生物科技技术有限公司
山羊抗鼠二抗	北京中杉金桥生物科技技术有限公司
山羊抗兔二抗	北京中杉金桥生物科技技术有限公司
硝酸纤维素膜（PVDF 膜）	美国 Amresco 公司
ECL 化学发光超敏显示试剂盒	美国 Thermo Fisher Scientific 公司

1.1.2.2 实验仪器

名称	来源
BCD268K 型普通冰箱	青岛海尔集团
Leica CM1950 石蜡切片机	Germany Leica Corporation
DK600 型电热恒温水浴箱	上海实验器械总公司
IOIA-3 型电热鼓风干燥箱	上海实验器械总公司
干燥箱	三木科学仪器厂
通风操作台	拉贝尔仪表器厂
微型漩涡混合仪	上海沪西精密分析仪器厂
AB204E 型电子天平	Switzerland Mettler Toledo Corporation
BP-II 型微量药物天平	上海第二医用激光仪器厂

BP-II 型架盘药物天平	上海第二医用激光仪器厂
902N0210 型磁力搅拌仪	Fisher Scientific，U．S．A．
LABCoNCO 纯水制水机	BIORAD，U．S．A．
普通微波炉	中国格兰仕公司
JK-6 型生物组织烤片机	武汉俊杰电子有限公司
生物病理组织烘片仪	武汉俊杰电子有限公司
生物病理组织漂片仪	武汉俊杰电子有限公司
090-135.001 型倒置相差显微镜	Germany Leica Corporation
光学显微镜	Japan Olympus Corporation
光学显微照相系统	Japan Nikon Corporation
载玻片	江苏世泰实验器材有限公司
盖玻片	江苏世泰实验器材有限公司
10cm 细胞培养皿	美国 Thermo Fisher Scientific 公司
15ml 离心管	美国 Thermo Fisher Scientific 公司
50ml 离心管	美国 Thermo Fisher Scientific 公司
细胞冻存管	美国 Thermo Fisher Scientific 公司
细胞计数板	美国 Thermo Fisher Scientific 公司
盖玻片	江苏世泰实验器材有限公司
090-135.001 型倒置相差显微镜	Germany Leica Corporation
YG-857 型超净工作台	江苏苏州长桥净化设备厂

L530 型台式高速离心机	湖南长沙湘仪离心机仪器有限公司
HW0301T 型 CO_2 细胞培养箱	美国 Thermo Fisher Scientific 公司
DK600 型电热恒温水浴箱	上海实验器械总公司
BCD268K 型普通冰箱	青岛海尔集团
UTL 型超低温冰箱	美国 Thermo Fisher Scientific 公司
SS325 型高压蒸汽灭菌锅	日本 tomy 公司
FM1200E 制冰机	苏州星琦电机有限公司
10μL 微量移液器	德国 Eppendorf 公司
100μL 微量移液器	德国 Eppendorf 公司
1000μL 微量移液器	德国 Eppendorf 公司
通风操作台	拉贝尔仪表器厂
5810R 型低温超速离心机	德国 Eppendorf 公司
干式金属浴恒温仪	杭州佑宁科技有限公司
7900HT 荧光定量 PCR 仪	美国应用系统生物科技有限公司
WD9405B 型水平摇床	北京市六一仪器厂
全自动酶标仪	美国 Thermo Fisher Scientific 公司
分光光度计	日本 Takara 生物科技公司
稳压 SDS-聚丙烯酰胺凝胶电泳仪	美国伯乐公司
Western Blot 转膜仪	美国伯乐公司
Tanon 全自动化学发光图像处理系统	上海市天能科技有限责任公司

BP-II 型微量药物天平	上海第二医用激光仪器厂
WD9403C 紫外分析仪	北京市六一仪器厂
液氮罐	美国 Thermo Fisher Scientific 公司

2.1.2.3 引物

引物名称	引物序列(5’—3’)
METTL3- Forward Primer	CGCGCCTTATTCGAGT
METTL3- Reverse Primer	TAGATCCAAGTGCCCCGAGT

1. 1. 3 实验方法

1.1.3.1 组织蜡块的制作及切片

（1）首先收集新鲜的组织，通过肉眼及触感大致分辨肿瘤及正常组织。选取合适的组织，切取约 0.8cm*0.8cm*0.3cm 大小，完全浸泡在 10%福尔马林溶液之中固定。

（2）随后将固定后的组织水洗 10 分钟，放入塑料包埋框里，之后放入分别放入浓度梯度乙醇中去。分别为 75%乙醇 10 分钟，85%乙醇 10 分钟，95%乙醇 10 分钟，100%无水乙醇 10 分钟，100%无水乙醇 10 分钟。

（3）后将脱水后的组织浸入二甲苯透明剂中。

（4）将石蜡放置在 58℃的恒温烘箱中加热融化。将经过透明处理的组织放入烘箱内的液体石蜡容器内。后将融化的石蜡导入金属包埋框，将浸蜡的组织平整放入金属包埋框中。待石蜡凝固后，将其放入冰箱过夜第二日取出。

（5）检测石蜡切片机，并安装刀片。将修整的石蜡组织块从冰箱取出，安放在切片机上，切片厚度调整至 4μm，匀速转动摇杆切片。用毛笔轻柔的摘下切片，用镊子小心捏起蜡带，轻轻展平在 45℃的恒温水浴锅中，待蜡带自然展平，用载玻片将水中的蜡带捞到载玻片中部，做上标记放在置架上，后放入 65℃的恒温烘箱内烤片 30 分钟。

1.1.3.2 组织切片免疫组织化学染色

（1）将组织切片放入 65℃的恒温烘箱内烤片 40 分钟，然后依次放入二甲苯缸一 20 分钟 ，二甲苯缸二 20 分钟，无水乙醇 10 分钟，无水乙醇 10 分钟，95%乙醇 5 分钟，85%乙醇 5 分钟，75%乙醇 5 分钟。之后浸泡在去离子水中 5 分钟，倒掉并换新的去离子水浸泡 5 分钟，在浸泡在磷酸盐缓冲溶液（PBS）5 分钟。

（2）然后将脱蜡处理后的组织切片置于湿盒中，在组织处滴上 3%的过氧化氢溶液，溶液覆盖满组织，在湿盒中室温孵育 10 分钟，将内源性氧化物酶清除。孵育结束，用 PBS 浸润清洗 3 次，每次 5 分钟，尽量将过氧化氢洗净。后将切片浸入 0.01M 枸橼酸缓冲液中，放入微波炉，以中大火力加热煮沸，保持沸腾状态约 10 分钟。后放置室温冷却，再用 PBS 浸润反复冲洗 3 次，每次 5 分钟。

（3）配置一抗工作液，使用 Anti-METTL3 抗体(ab195352)按照 1:500 使用 PBS 稀释。将组织切片去除多余水分，平放在湿盒中，将一抗工作液滴在组织上，均匀覆盖整个组织。盖上湿盒放入 4℃冰箱过夜。

（4）第二天从冰箱拿出，放置室温复温，除去一抗，用 PBS 浸洗 3 次，每一次 10 分钟。后擦去多余液体，水平放于湿盒中，滴加二抗，保证完全均匀覆盖组织，在室温中孵育 1 小时。

移去二抗，放入 PBS 中漂洗 3 次，每次 10 分钟。

（5）配置新鲜 DAB 工作液，滴在移除多余液体的组织切片上。可在显微镜下观察染色效果，达到理想效果终止染色反应。后用 PBS 反复冲洗 3 次，每次 5 分钟。然后滴加苏木素溶液复染大约 3 分钟。之后放入自来水冲洗。

（6）之后放入去离子水中 5 分钟，依次放入 75%乙醇 5 分钟，85%乙醇 5 分钟，95 乙醇 5 分钟，无水乙醇 5 分钟，无水乙醇 5 分钟，二甲苯 10 分钟，二甲苯 10 分钟。然后滴上中性树胶，用盖玻片覆上，用镊子小心挤出气泡，完成封片，放在通风橱内晾干。之后可在显微镜底下观察结果。

1.1.3.3 细胞培养技术

细胞复苏

准备细胞培养材料，DMEM 高糖培养基，1640 培养基,胎牛血清，25ml 培养瓶，10ml 离心管，移液管等置于清洁超净台中，紫外灯下照射 30 分钟。打开 37℃恒温水浴锅。配置 10%胎牛血清培养基（使用 1640 培养基或者 DMEM 高糖培养基依据细胞需要配置）。

在本研究中一句相关文献及 ATCC 建议 BPH1 细胞系使用 10%胎牛血清 DMEM

高糖培养基，LNCaP 细胞系、22-RV1 细胞系、PC3 细胞系、DU145 细胞系、C4-2 细胞系均使用 10%胎牛血清 1640 培养基，而 LNCaP-AI 细胞系使用 10%去雄激素胎牛血清 1640 培养基。穿戴防护用具，自液氮中取出复苏细胞，放在 37℃水浴锅中，快速解冻，可以轻轻摇晃，勿将盖口接触水，使其于 1 分钟内完全解冻。然后 75%酒精消毒后放入无菌超净台中。打开冻存管，用新鲜配置 10%胎牛血培养基按照 1：10 比例将复苏细胞稀释，轻轻吹打均匀后放入室温离心机中，以 1000rpm，离心 3 分钟。后再次 75%酒精消毒，放入无菌超净台操作。倒去离心管中培养基，换以新的新鲜培养重悬。后移入 25ml 培养瓶中，加入约 8ml 培养基。轻轻摇晃培养瓶使细胞均匀平铺于培养瓶底部。后移至 5% CO_2 37℃恒温培养箱中。

细胞培养及传代

准备细胞培养材料，配置的新鲜培养基，胰酶（0.25%Trypsin-EDTA），PBS 缓冲液，25ml 培养瓶，10ml 离心管，移液管等置于清洁超净台中，紫外灯下照射 30 分钟。依据细胞生长情况及营养消耗情况及细胞类型，选择更换培养基时间，或者进行传代。本实验所用细胞均为贴壁细胞，一下均为贴壁细胞换液传代操作。

1. 对于新复苏的贴壁细胞，于第一个 24 小时换液，首先废弃旧培养基，使用 1-2ml 经灭菌的 PBS 轻柔的冲洗 1-2 遍，注意不要将细胞吹起。然后移除 PBS 缓冲液，换上 8ml 新鲜培养基。后将细胞放入 5% CO_2 37℃恒温培养箱中培养。

2. 对于生长情况良好，铺满培养瓶底部（90%）细胞呈单层生长，可以按 1:3-1：4 比例传代。首先废弃原有培养基，用 1ml 经灭菌的 PBS 轻轻冲洗 3 次，不要直接冲洗细胞，小心不要冲刷下细胞。后吸除 PBS，加入 1ml 0.25%Trypsin-EDTA，放入 5% CO_2 37℃恒温培养箱中消化 3 分钟。后可在相差显微镜底下观察细胞消化情况。待消化满意再加入按照 1:1 比例加入含 10%胎牛血清的培养基，轻柔的吹打下贴壁细胞，收集细胞悬液到 10ml 离心管中放入离心机。以 1000rpm 低速离心 3 分钟。弃掉上层培养基，加入 3ml 新鲜培养基（10%胎牛血清 1640 或 DMEM 培养基），用移液管轻轻吹打使细胞重悬。后平均分配到准备好的 3 个 25ml 培养瓶中，每个培养瓶中加入 1ml 细胞悬液，再加入 9ml 新鲜培养基。轻轻摇晃使细胞均匀铺满整个培养瓶底部。放入 5% CO_2 37℃恒温培养箱中。

细胞冻存

选择处于对数期的细胞经处理放于液氮中长期储存。准备细胞培养材料，完全

培养基，胎牛血清，二甲基亚砜（DMSO），0.25%Trypsin-EDTA，PBS 缓冲液，细胞冻存管，10ml 离心管，移液管等置于清洁超净台中，紫外灯下照射 30 分钟。选择生长良好，处对数期的细胞，弃掉原有培养基，用 1ml 经灭菌的 PBS 轻轻冲洗 3 次。后吸除 PBS，加入 1ml 0.25%Trypsin-EDTA，放入 5% CO_2 37℃恒温培养箱中消化 3 分钟。后可在相差显微镜底下观察细胞消化情况。待消化满意再加入按照 1:1 比例加入含 10%胎牛血清的培养基，轻柔的吹打下贴壁细胞，收集细胞悬液到 10ml 离心管中放入离心机。以 1000rpm 低速离心 3 分钟。用 90%胎牛血清+10%DMSO 配置冻存液。细胞离心后，弃掉上层培养基，加入 2ml 冻存液，轻柔吹打，重悬细胞，后平均分装在两支冻存管中，冻存管标记上细胞名及时间。将冻存管放置 4℃冰箱 30 分钟，-20℃冰箱 30 分钟，-80℃冰箱过夜，最后存放至液氮中长期保存。

1.1.3.4 细胞 RNA 提取

（1）准备氯仿，置于-20℃异丙醇，新配 75%乙醇，无酶水（RNAse free），PBS 缓冲液，TRIZOL，及移液管，1.5ml EP 管（RNAse-free）等。

（2）选择生长良好，90%丰度的细胞，移除原有培养基，使用 PBS 轻柔冲洗 3 次，吸尽 PBS，加入 1ml TRIZOL，在室温中放于摇床 5-10 分钟，使其充分裂解。

（3）将裂解了细胞的 TRIZOL 试剂转移至 1.5ml EP 管中，加入 0.2ml 氯仿，后盖上盖子，剧烈摇晃，充分混合，放于 4℃冰箱静置 15 分钟。后放入 4℃离心机中，以 12000g 的条件，离心 15 分钟。

（4）小心取出 EP 管，勿倾倒混合，吸取上层透明水相上清 0.5ml 移至新的 1.5ml EP 管中。

（5）加入 0.5ml 预冷异丙醇，并于 4℃冰箱静置 10 分钟。后放入 4℃离心机中，以 12000g 的条件，离心 10 分钟。

（6）后去除 EP 管中上清液，尽量吸尽。加入 1ml 新配 75%乙醇（RNAse free），简单地震荡或者吹打。后放入 4℃离心机中，以 7500g 的条件，离心 5 分钟。此步骤可进行两次。

（7）后吸尽上清，在空气中干燥 5-10 分钟。之后用 20ul 无酶水溶解。再置于 60 摄氏度金属浴加热 15 分钟。取 2ul 用于分光光度计检测其吸光度。测算 RNA 浓度，并记录。

1.1.3.5 RNA 反转录 DNA

准备制备好的 RNA，反转录试剂盒（Thermo K1621 RevertAid First Strand cDNA Synthesis Kit），包括，恒温金属浴，无酶水，1.5ml EP 管等。在一支干净的 1.5ml EP 管中依次加入 5ul RNA 溶液，1ul Oligo (dT)18 primer 试剂，6ul 无酶水。盖上管盖震荡混匀，经过简单地短暂离心，放入 65℃恒温金属浴中加热 5 分钟。加热后再次简单短暂离心后放冰上冷却。后依次加入 4ul 5X Reaction Buffer 试剂，1ul RiboLock RNAse Inhibitor (20 U/μL)试剂，2ul 10mM dNTP Mix 试剂和 1ul RevertAid M-MuLV RT (200 U/μL)试剂。盖上盖子后进行充分的混匀，通过震荡混匀，简单离心放入 42 摄氏度恒温金属浴中孵育 1 小时，然后 70℃加热 5 分钟。

1.1.3.6 实时荧光定量核酸扩增检测系统（Real-time Quantitative PCR Detecting System qPCR）

（1）首先根据样本数量以及目标基因，预先排好样本顺序。每一个样本及目的基因需要三个复孔，尽量将一个样本排于同一行或者同一列，同理目标基因也尽量在同一列或者同一行。并且需要不同批次实验。

（2)依据之前反转录得到的 cDNA 按照 1：10 稀释。随后准备无酶水，SYBR Green PCR Master Mix，目标基因上游引物，目标基因下游引物，依据说明稀释成浓度 10M 工作液。依据 H2O 3ul，SYBR Green PCR Master Mix 5ul，上游引物 0.5ul (10uM),下游引物 0.5ul (10uM)以及 cDNA 1ul 配置成总体积为 10ul 的反应体系。计算好加样份数，按照上述比例配置，先预混 H2O+SYBR Green PCR Master Mix+上游引物+下游引物。一般多配一份。随后按照排列的计划，使用微量加样器加入 PCR 96 孔板。

（3）随后将稀释后的 cDNA 依据排列加样。需要确保 cDNA 量足够，不可中途重新稀释。随后使用配套透明塑料膜封好 PCR 96 孔反应板，用卡片或者压板块将每个孔都封死。操作过程中尽量做到避光操作，手不要触碰板底。

（4）将板边缘靠着涡旋震荡机，小心混匀，随后放入离心机中以 1000rpm 离心 5 分钟。准备上机。

（5)使用美国应用系统生物科技有限公司的 7900HT 荧光定量 PCR 仪完成 qPCR 实验，设定 95℃预变性 2 分钟，然后 95℃变性 30 秒，58℃退火 30 秒，72℃延伸 30 秒进行扩增 45 个循环，最后 72℃2 分钟终止延长。

（6）第一次实验需要进行 qPCR 测试其特异性和扩增效率。根据熔解曲线判断

引物特异性，选择标准为：单峰且峰形偏窄。

（7）完成后保存数据，拿出样本关闭 7900HT 荧光定量 PCR 仪及电脑。

1.1.3.7 细胞蛋白质提取

（1）准备一次性细胞刮刷，冷 PBS，RIPA 裂解液，1.5ml EP 管等物品。

（2）首先选取长满的一培养皿贴壁细胞，大约在 10^6 个细胞。小心移除原有培养基，用准备好的冰 PBS 沿皿壁加入培养皿，用来冲洗细胞，除去培养基及代谢产物，冰 PBS 冲洗两次。

（3）按照 100:1 在 RIPA 裂解液中加入 PMSF，因为 PMSF 在水溶液中易降解，所以须现用现配。一般我们在 200ul RIPA 裂解液中加入 2ul PMSF。后将配置好的 200ml RIPA 工作液加入吸尽冰 PBS 的培养皿中，放入 4 摄氏度冰箱内的摇床上，摇晃半小时。期间每十分钟敲打摇晃培养皿确保裂解液能均匀充分接触整个皿底，使细胞得到充分裂解。

（4）后使用一次性细胞刮刷顺着皿底完全收集细胞裂解液，使用移液枪将裂解液收集至 EP 管中。放入 4 摄氏度的高速离心机中，按照 14000g 运行半小时。然后收集上清液。利用分光光度计检测蛋白浓度。然后在管壁上标上蛋白来源，时间以及浓度，将其分装冻存，避免反复冻融。

1.1.3.8 蛋白质印迹实验（Western Blot）

（1）蛋白样本的制备

1.准备标准蛋白 2mg/ml。用去离子水稀释准备好的标准蛋白。倍比稀释得到终浓度为 2mg/ml、1mg/ml、0.5mg/ml、0.25mg/ml、0.125mg/ml。

2.使用成品考马斯亮蓝染液（Bradford），使用 96 孔板作为检测容器，以每个样品设置三个重复孔，在加样孔中没孔加入 200ul 成品 Bradford 染液。然后依次加入终浓度为 2mg/ml、1mg/ml、0.5mg/ml、0.25mg/ml、0.125mg/ml 的标准蛋白样品各 2ul 以及空白对照。同样 3 复孔加入 200ul 成品 Bradford 染液，再加入 2ul 实验样品蛋白（之前提取的前列腺癌细胞蛋白质）。通过轻微的震荡或者用微量加样器进行吹打，保证蛋白样本与 Bradford 染液充分接触混匀，一般肉眼能够看到 Bradford 染液明显的颜色改变，同时要避免气泡的产生，可以利用针头挑破气泡。

3.使用酶标仪或者分光光度计测定 A595nm 的吸光度。然后记录数值，利用

EXCEL 分析数据，绘制出标准蛋白浓度曲线。然后计算出实验蛋白样品的浓度。
4.计算实验蛋白量，制备蛋白样本。我们选用 30ug 蛋白为最终上样量。取 30ug 实验蛋白样品以浓度换算体积，按照 4:1 加入 5x 蛋白上样缓冲液。将 30ug 实验蛋白样品与 5x 蛋白上样缓冲液加入 0.5ml EP 管中，利用涡旋器震荡混匀，再进行简单短暂离心。放入 95℃恒温金属浴中，加热 5 分钟。制备完成样品可保存在-80℃冰箱中保存。
（2）制备 SDS 聚丙烯酰胺凝胶
首先选取配套电泳长短灌胶玻璃板，检查有无裂痕破损及缺口。对齐玻璃板后放入制胶夹中卡紧，放置在垂直的制胶架上，加入去离子水，检查灌胶玻璃板之间与胶条之间有无缝隙是否漏水。确认无漏水后，倒掉去离子水，滴尽，然后配置分离胶。
依据实验目标蛋白大小选择分离胶浓度。依据下表配置分离胶。本研究中我们选用 10%分离胶。以制备一块 SDS 聚丙烯酰胺凝胶为例，依次在试管中加入 1.9ml 去离子水（H_2O），1.7ml 30%丙烯酰胺，1.3ml 1.5M Tris/HCl（PH 8.8），50ul 10%十二烷基硫酸钠（SDS），50ul 10%过硫酸铵（APS），最后加入 2ul 四甲基乙二胺（TEMED）。将其混匀加入灌胶玻璃板中，要在上方留出约 2cm，用水或者无水乙醇封口，隔绝空气接触。室温静置 30 分钟，直到能看到上层液体与下层的凝胶出现清晰地分界线，表明分离胶凝固了。
然后配置 5%浓缩胶，依据下表浓缩胶比例，依次加入 1.46ml H2O，330ul 30%丙烯酰胺，170ul 1.5M Tris/HCl（pH 6.8），20ul 10% SDS，20ul 10%APS，最后加入 2ul TEMED。将其混匀，然后倒去灌胶板中的用于液封的水，倒尽并用吸水纸吸干，再倒入配置好的 5%浓缩胶，水平插入干净的 10 孔加样梳。注意要缓慢插入，排进中间的空气，不留空气。待浓缩胶凝固，可将制备好的凝胶浸在水中放于 4 摄氏度冰箱保存。
蛋白质凝胶电泳
1. 配制电泳缓冲液，取一干净 2L 量筒，置于电子天平上，调零。依次加入 15g Tris，72g 甘氨酸，5g SDS。然后加入去离子水定容至 1L，配置了 5x 电泳缓冲液。使用时将其稀释成为 1x 电泳缓冲液。
2. 准备电泳槽，电泳架，将灌胶玻璃板短板朝里固定于电泳架上。若只有一块电泳凝胶，则对侧需要放上替代的塑料的替代凝胶板，防止短路。然后倒入稀释后的 1x 电泳缓冲液。从两块凝胶中间加缓冲液，直到两板之间加满，电泳槽

中加至电泳槽壁指示刻度。双手缓慢平稳抓住加样梳向上提起，轻轻拔出，小心的保证浓缩胶上的上样孔完整。可以用移液管吸少许 1x 电泳缓冲液冲洗上样孔检查上样孔完整性。后装上上样梳上样。使用微量加样器吸取 5ul 蛋白 marker 加入第一道上样孔。随后加入制备好的实验蛋白样本。上样需要轻柔缓慢，防止配平好的蛋白样本因为上样不规范而损失。随后移去上样架，盖上电泳槽的盖子，注意电极方向，正极对正极，负极对负极。打开电源，先以 60V 电压，运行 30 分钟，由样品电泳完全超过浓缩胶为准。后调整电压至 90V，运行 90 分钟左右。以蓝色溴酚染料电泳跑到接近凝胶底部为准。此时停止电泳。

蛋白质电转

1. 配置 10x 电转缓冲液，取 2L 干净量桶，放于电子天平上，调零后依次加入 30.3g Tris 和 144g 甘氨酸，随后加入去离子水定容至 1L。使用时量取 100ml 10x 转膜缓冲液，加入 100ml 甲醇，再加入 800ml 去离子水，制成 1x 转膜缓冲液。裁剪大约 6*8cm 大小 PDVF 膜，浸泡于甲醛中激活。随后准备电泳电转槽，以及电转夹，海绵垫两块，以及裁剪同等大小的厚滤纸两张。将电转膜夹子打开，黑面水平放于桌上，依次放上浸湿后的海绵垫，再放上一张浸湿后的滤纸。打开电泳槽盖子，取出电泳凝胶玻璃板，放在操作的搪瓷盆中，玻璃板短板朝上，使用撬板沿着上样孔这一边小心撬开灌胶玻璃板，取下玻璃板短板。然后用撬板薄边沿着浓缩胶分离胶分界线谨慎切割凝胶。去掉浓缩胶，小心剥下分离胶，将他平稳移到电转夹黑色面的滤纸上，不要留有气泡，下层分别是滤纸，海绵垫，以及电转夹黑面。随后将经过甲醛激活过的 PDVF 膜附在 SDS 聚丙烯酰胺凝胶上，保证覆盖整个凝胶，并用翘板将凝胶与膜之间的气泡去除干净。然后依次附上厚滤纸，海绵垫，夹上电转夹，安装固定在电转架上，电转夹子的黑面要对着电转架子的黑色部分，电转夹子的透明面对着电转架子的红色部分，放入电泳电转槽中。转移凝胶过程中保持凝胶在电转缓冲液中进行。随后加入稀释成 1x 的转膜缓冲液，盖上电泳电转槽的盖子。准备大量冰块及水，将整个电泳电转槽放置在冰水混合物中，为了防止电转时产热导致蛋白降解。再接通电源，注意电极正负，红对红，黑对黑。以限定电流 250mA 模式转膜 150 分钟。

蛋白印迹显影

配置 Tris-HCl 缓冲液,取 2L 干净量桶，放于电子天平上，调零后依次加入 12.15g Tris-HCl 和 146.4g NaCl，然后加入去离子水定容至 1L，得到 5xTBS 缓冲液。随后用去离子水稀释到 1xTBS 缓冲液，按照 1000:1 每 1L TBS 缓冲液加入 1ml

Tween-20，配置成 1x TBST 缓冲液。

将转有蛋白质的 PDVF 膜从电转夹上取下，以与 SDS 聚丙烯酰胺凝胶直接接触的一面为正面，保证正面朝上并剪角来做标识。将膜放在洗脱容器中，浸入 TBST 缓冲液中，放置于室温摇床上，洗脱 3 次，每次 5 分钟。

用电子天平称量脱脂奶粉 2.5g，用 TBST 缓冲液溶解，配置成 5%脱脂奶粉封闭液。将经过 TBST 洗三次的膜放入 5%脱脂奶粉封闭液中，在室温的摇床上，封闭 1 小时。随后回收 5%脱脂奶粉封闭液，并用 TBST 再次漂洗三次，每一次 10 分钟。同时按照抗体说明书按比例稀释一抗。本实验中使用 METTL3 兔源一抗，按照 1:1000 使用 TBST 缓冲液稀释。内参选用 GAPDH 为鼠源一抗，按照 1:10000 使用 TBST 缓冲液稀释。将洗脱过的膜按照蛋白 Marker 大约范围进行裁剪，然后将膜放入相应的目标一抗中，放置在 4 摄氏度冰箱的摇床上孵育过夜。

第二日回收一抗，用 TBST 缓冲液洗膜，三次每次 10 分钟。随后将条带放入相应二抗孵育（依据一抗来源选择羊抗兔二抗或者羊抗鼠二抗），室温摇床上孵育 1 小时。随后回收二抗，TBST 缓冲液洗膜三次，每一次 10 分钟。

打开曝光机，预冷至-40 摄氏度。新配置化学发光显影液（ECL），A 液 B 液按照 1:1 避光混合，滴在膜上，放入曝光机中显影曝光。

1.1.3.9 统计学方法

采用 Image J 对实验结果进行半定量分析。

采用 Graphpad prism 5 对实验结果进行统计学分析，χ^2 检验分析计数资料的组间差异，t 检验分析计量资料的组件差异。均以双侧 $p < 0.05$ 认为差异有统计学意义。

1.2 结果

1.2.1 前列腺癌组织与良性前列腺病变组织中 METTL3 蛋白表达有差异

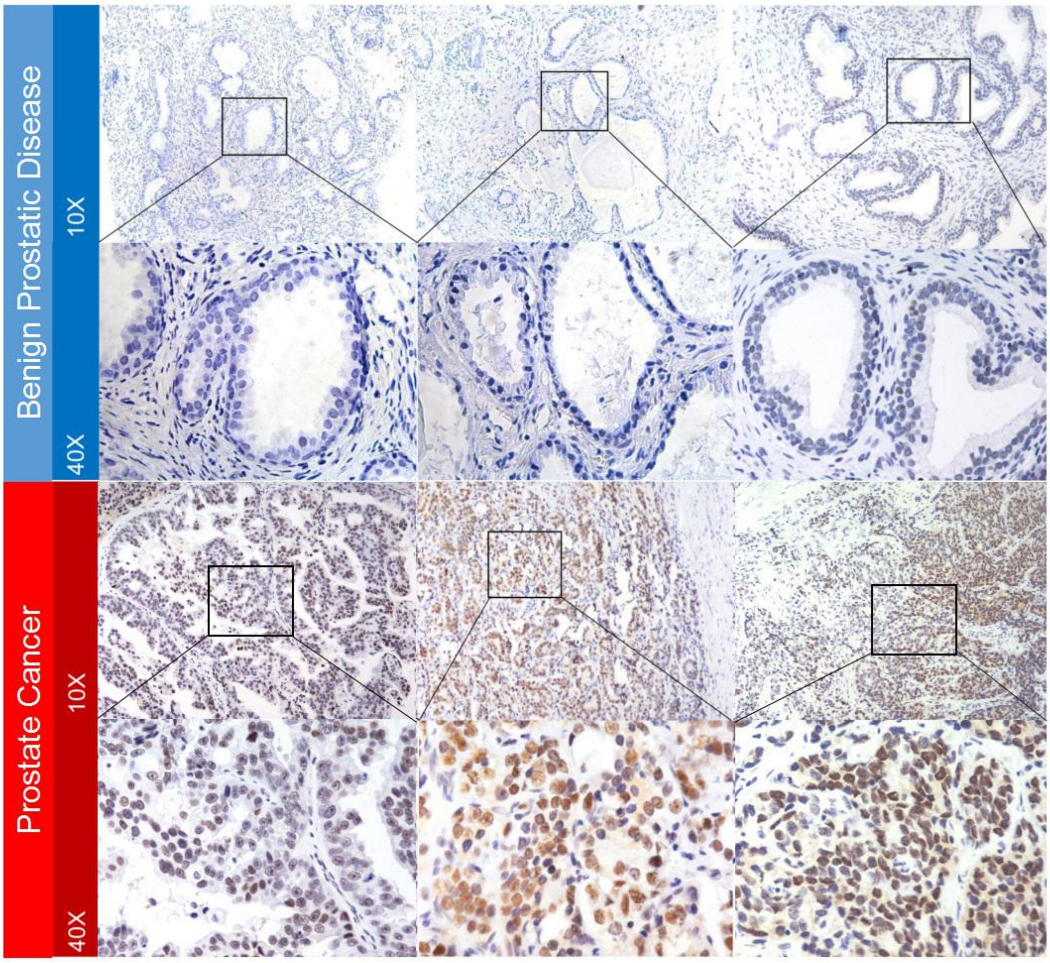
Benign Prostatic Disease
10X
40X
Prostate Cancer
10X
40X

图 1.1

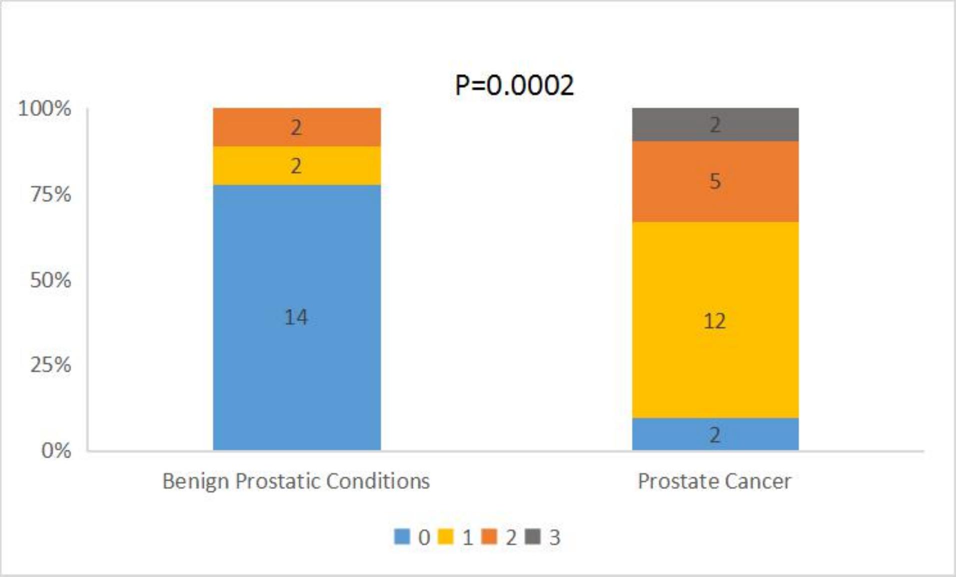
P=0.0002
100%
75%
50%
25%
0%
2
2
14
2
5
12
2
Benign Prostatic Conditions
Prostate Cancer
0
1
2
3

图 1.2

图 1.1 是前列腺良性组织与激素敏感型前列腺癌组织的免疫组化结果显示 METTL3 水平表达。

图 1.2 显示通过 Image J 分析免疫组结果，分为良性前列腺病变及前列腺癌组，18 例良性前列腺病变及 21 例前列腺癌组织。通过 Image J 于前列腺良性病变组中 12 例 METTL3 蛋白表达阴性，2 例弱阳，两列阳性；于前列腺癌组 METTL3 蛋白表达 2 例阴性 12 例弱阳 5 例阳性 2 例强阳性。经过 Chi 检验 p=0.00002，两组间有显著差异。METTL3 蛋白在人前列腺组织中，前列腺癌组中比良性前列腺病变组表达更高。与良性前列腺组织相比较，METTL3 在前列腺癌组织中主要呈高表达，差异具有统计学意义（P<0.001）

1.2.2 前列腺癌细胞系中 METTL3 在 RNA 水平表达高于良性前列腺病变细胞系

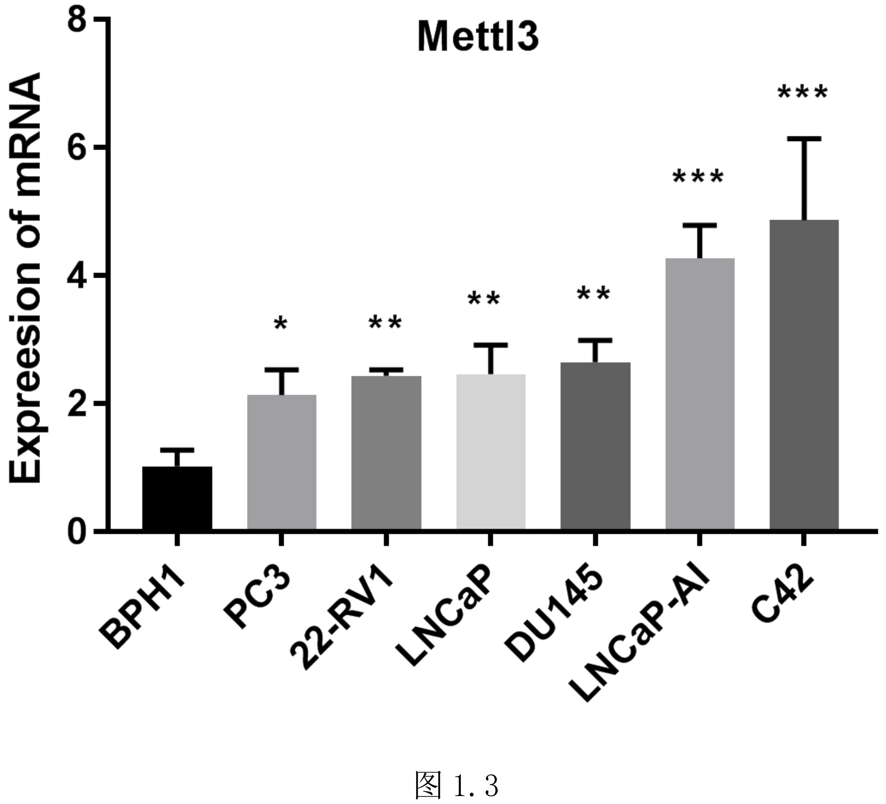

图 1.3

图 1.3 是各个细胞系中 METTL3 mRNA 的表达水平。通过 qPCR 检测 METTL3 在各前列腺组织细胞系中 RNA 的水平，各前列腺癌细胞系均显著高于良性前列腺病变细胞系 BPH1。

1.2.3 前列腺癌细胞系中 METTL3 在蛋白水平水平表达高于良性前列腺病变细胞系

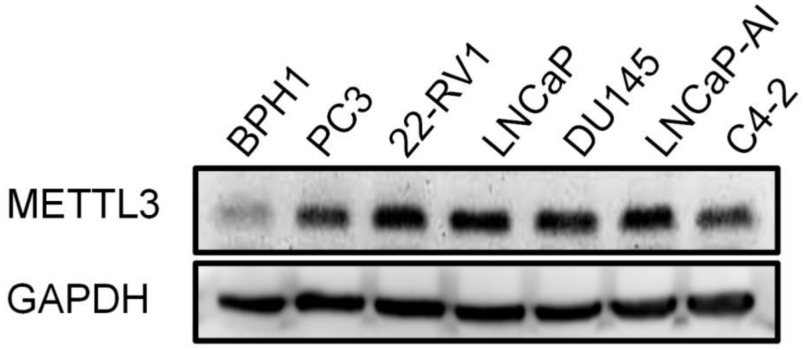

图 1.4

图 1.4 是各个细胞系中 METTL3 蛋白表达水平。通过 Western Blot 检测 METTL3 在各前列腺组织细胞系中蛋白水平，各前列腺癌细胞系均显著高于良性前列腺病变细胞系 BPH1。

1.3 讨论

前列腺癌发病率在世界上居高不下，是全球范围内发病率最高的泌尿系肿瘤，在西方国家成为男性的第一大肿瘤。虽然欧美发达国家的前列腺癌发病率高于发展中国家，中国是一个前列腺癌发病率较低的国家，但近年来，我国一些主要城市的前列腺癌发病率迅速上升，前列腺癌发病率在男性肿瘤中已经跃居第六。前列腺癌已经严重危害男性的健康。其相关确定的危险因素有年龄，种族以及遗传。但其外界危险因素还没有明确的定论。环境污染、不良生活习惯和长期压力负荷的加重是否相关[1-6]。

而近年来表观遗传学的兴起，他是门不涉及遗传信息改变，研究遗传信息可逆性的表型改变的学科[7]。包括 DNA 和 RNA 甲基化、组蛋白修饰、非编码 RNA 修饰和染色质重排等。其中 DNA 甲基化和组蛋白修饰研究较多。DNA 中的 5mC 已被证明广泛影响许多癌症基因表达[2]。而在 RNA 层面也有类似于 DNA 5mC 的表观修饰。这些 RNA 修饰普遍存在于真核生物中，极大地丰富了 RNA

的功能和遗传信息的多样性。RNA 甲基化是 RNA 修饰的主要形式，其中 m6A 修饰约占 mRNA 修饰的 80%，值得近年来的广泛关注和研究。

METTL3（MT-A70）是一种 70 kDa 的蛋白，METTL3 是哺乳动物细胞中 m6A 甲基化转移酶复合物。METTL3 从酵母到人的真核生物中具有高度保守性。在 HeLa 细胞中敲低 METTL3 的表达，总 m6A 水平下降了约 30%。如今已经有越来越多的报道表明 m6A 修饰在各类肿瘤中起到重要的作用，而 METTL3 作为甲基转移酶复合物的关键蛋白在其中起到了关键作用。2017 年，Du 等报道 METTL3 在非小细胞肺癌组织中的表达高于癌旁组织。METTL3 在 NSCLC 组织中的表达与 miR-33a 的表达呈正相关。MiR-33a 能够在 mRNA 和蛋白水平上降低 METTL3 的表达，揭示了 miRNA 调控 METTL3 的新机制[29]。Cui 研究团队报道，METTL3 或 METTL14 的下调明显增强了胶质母细胞瘤细胞的干细胞生长和自我更新能力，促进肿瘤的发展，而 METTL3 的过表达则相反[15]。

因为 METTL3 存在着甲基基团，并且前列腺癌中有着较高的表达，通过利用美国发起的癌症基因图谱（TCGA）数据进行简单分析，我们发现在前列腺癌与正常癌旁组织中 METTL3 表达含量有着较明显的差异。我们考虑 METTL3 是否可能是前列腺癌除了前列腺特异抗原（PSA）以外另一有效的生物标记，或者有着潜在可能促进前列腺癌的发生发展。在我们研究中心，我们首先收集了前列腺癌患者组织标本，及良性前列腺病变的组织标本。前列腺癌标本均来自于首次检测出前列腺癌患者，在行前列腺癌根治性切除术前并未接受过手术或者药物趋势治疗、药物抗雄激素治疗、放射治疗、化学治疗等治疗，均为激素敏感性前列腺癌患者。而良性前列腺病变组织来自于癌旁组织及前列腺增生组织。这些组织样本经过免疫组织化学染色，检测组织中 METTL3 表达水平。实验结果显示，在良性前列腺病变组中绝大多数为 METTL3 表达水平为阴性，18 例中仅有两例为弱阳性。而在激素敏感型前列腺癌中，仅有两例为阴性。大多都有不同程度的 METTL3 表达。与 Gleason 评分并线性关系。主要原因认为收集前列腺癌组织标本均来自激素依赖性前列腺癌患者，评分都为 7-8 分。没有其他低评分前列腺癌组织的 METTL3 表达水平验证。因此我们的结果表明，在激素敏感型前列腺癌组织中 METTL3 表达水平升高。

那么 METTL3 水平的升高是否影响前列腺癌的发生发展。我们通过 qPCR 及 western 实验检测了 METTL3 在良性前列腺病变细胞系 BPH1 和各类前列腺癌细胞。结果显示各类前列腺癌细胞系中 METTL3 表达含量均与 BPH1 有明显

的差别，与我们人组织标本结果一致。

1.4 小结

激素敏感型前列腺癌中 METTL3 异常表达，相比于前列腺良性病变组织有明显升高。这一结论在人前列腺组织中，以及人前列腺癌细胞系中均得到证实。通过免疫组化实验，METTL3 在前列腺癌组织中主要表达于细胞核，是 RNA 调控的场所。METTL3 在前列腺癌的发生发展过程种起到重要的机制

二、METTL3 在前列腺癌细胞系中的增殖作用

通过慢病毒转染前列腺癌细胞建立稳敲低 METTL3 的细胞系。利用稳定敲低 METTL3 前列腺癌细胞系验证 METTL3 影响细胞增值能力。

2.1 对象和方法

2.1.1 研究对象

LNCaP 人前列腺 癌细胞系、C4-2 人前列腺癌细胞系，来自于美国组织培养中心（ATCC）

2.1.2 实验试剂及仪器

2.1.2.1 实验试剂

名称	来源
RPMI Media 1640 细胞培养基	美国 Gibco 公司
Gibco 热灭活胎牛血清（FBS）	美国 Gibco 公司
Penicillin-Streptomycin 双抗	美国 Gibco 公司
Trypsin-EDTA (0.05%)（胰酶）	美国 Gibco 公司
二甲基亚砜（DMSO）	北京索莱宝科技有限公司
磷酸盐缓冲液（PBS）	北京索莱宝科技有限公司
Trizol	美国 Invitrogen 公司
三氯甲烷（氯仿）	天津市光复科技发展有限公司
异丙醇	天津市光复科技发展有限公司
无水乙醇	苏州海百化工有限责任公司
RIPA 裂解液	美国 Thermo Fisher Scientific 公司

PMSF 蛋白酶抑制剂	美国 Thermo Fisher Scientific 公司
Bradford(考马斯亮蓝)缓冲液	美国伯乐公司
Loading buffer 缓冲液	北京索来宝试剂有限公司
Western-blot 蛋白 marker	北京索来宝试剂有限公司
SDS-PAGE 凝胶制备试剂盒	北京索来宝试剂有限公司
30%Acr/Bis(29:1)	北京索来宝试剂有限公司
1M Tris-HCl(PH6.8)	北京索来宝试剂有限公司
1.5 Tris-HCl(PH8.8)	北京索来宝试剂有限公司
10%SDS	北京索来宝试剂有限公司
10%PAGE 胶凝固剂	北京索来宝试剂有限公司
PAGE 胶促凝剂	北京索来宝试剂有限公司
ddH_2O	天津市泌尿外科研究所
甘氨酸	美国 Sigma 公司
三羟甲基氨基甲烷（Tris）	美国 Sigma 公司
Tween-20	美国 Sigma 公司
十二烷基磺酸钠	美国 Sigma 公司
脱脂奶粉	美国 Sigma 公司
四甲基偶氮唑（MTT）	北京索来宝试剂有限公司
RevertAid First Strand cDNA Synthesis Kit	美国 Thermo Fisher Scientific 公司

Taq MasterMix	美国 Thermo Fisher Scientific 公司
兔抗人 METTL3 单克隆抗体	abcam corporation
兔抗人 m6A 单克隆抗体	德国默克集团
鼠抗人 GAPDH 单克隆抗体	北京中杉金桥生物科技技术有限公司
山羊抗兔二抗	北京中杉金桥生物科技技术有限公司
山羊抗鼠二抗	北京中杉金桥生物科技技术有限公司
硝酸纤维素膜（PVDF 膜）	美国 Amresco 公司
ECL 化学发光超敏显示试剂盒	美国 Thermo Fisher Scientific 公司
METTL3 基因敲低慢病毒	北京合生基因生物科技有限公司

1.1.2 实验仪器

名称	来源
10cm 细胞培养皿	美国 Thermo Fisher Scientific 公司
T25 细胞培养瓶	美国 Thermo Fisher Scientific 公司
15ml 离心管	美国 Thermo Fisher Scientific 公司
50ml 离心管	美国 Thermo Fisher Scientific 公司
细胞冻存管	美国 Thermo Fisher Scientific 公司
6 孔细胞培养板	美国 Thermo Fisher Scientific 公司
24 孔细胞培养板	美国 Thermo Fisher Scientific 公司
96 孔细胞培养板	美国 Thermo Fisher Scientific 公司

细胞计数板	美国 Thermo Fisher Scientific 公司
盖玻片	江苏世泰实验器材有限公司
090-135.001 型倒置相差显微镜	Germany Leica Corporation
YG-857 型超净工作台	江苏苏州长桥净化设备厂
L530 型台式高速离心机	湖南长沙湘仪离心机仪器有限公司
HW0301T 型 CO_2 细胞培养箱	美国 Thermo Fisher Scientific 公司
DK600 型电热恒温水浴箱	上海实验器械总公司
BCD268K 型普通冰箱	青岛海尔集团
UTL 型超低温冰箱	美国 Thermo Fisher Scientific 公司
SS325 型高压蒸汽灭菌锅	日本 tomy 公司
FM1200E 制冰机	苏州星琦电机有限公司
10μL 微量移液器	德国 Eppendorf 公司
100μL 微量移液器	德国 Eppendorf 公司
1000μL 微量移液器	德国 Eppendorf 公司
通风操作台	拉贝尔仪表器厂
5810R 型低温超速离心机	德国 Eppendorf 公司
干式金属浴恒温仪	杭州佑宁科技有限公司
7900HT 荧光定量 PCR 仪	美国应用系统生物科技有限公司
WD9405B 型水平摇床	北京市六一仪器厂
全自动酶标仪	美国 Thermo Fisher Scientific 公司

分光光度计	日本 Takara 生物科技公司
稳压 SDS-聚丙烯酰胺凝胶电泳仪	美国伯乐公司
Western Blot 转膜仪	美国伯乐公司
Tanon 全自动化学发光图像处理系统	上海市天能科技有限责任公司
BP-II 型微量药物天平	上海第二医用激光仪器厂
WD9403C 紫外分析仪	北京市六一仪器厂
液氮罐	美国 Thermo Fisher Scientific 公司

2.1.3 实验方法

2.1.3.1 病毒转染细胞

（1）准备细胞培养所需物品，以及微量加样器，于生物安全柜中紫外线照射 30 分钟；

选择合适细胞，25cm 细胞培养瓶中细胞长至大约占培养瓶底约 50%-60%。穿戴隔离衣，戴好帽子口罩及手套，佩戴护目镜。

（2）取两瓶细胞，一瓶为空白对照，一瓶为实验组。移去原有旧培养基，使用无菌 PBS 溶液清洗两次，去除残留培养基。后各加 6ml 无双抗 10%FBS 培养基。

（3）在对照组细胞培养瓶中加入空病毒 20ul，在实验组细胞瓶中加入 20ul METTL3 干扰病毒。标记后放入 37℃ 5% CO2 培养箱中。

（4）待培养 48 小时候，取出细胞培养瓶，在荧光显微镜下观察有荧光细胞数目。当带有荧光细胞达到 90%我们认为是满意的转染效率。

（5）随后使用嘌呤霉素筛选稳定细胞系。

首先试验选择嘌呤霉素最佳浓度。选择处于对数生长期的细胞，依照细胞传代方式，去除原有旧培养基，使用无菌 PBS 溶液清洗三次。使用胰酶消化细胞，待贴壁细胞消化为细胞悬液，利用含有胎牛血清的完全培养基终止胰酶消化。将细胞悬液移至一次性无菌离心管中，以室温，1000rpm，3 分钟离心细胞。离心之后去掉上层液体，加入 3ml 完全培养基，重悬细胞，使其成文均匀的细胞悬液。

用一新的无菌离心管取 1ml 细胞悬液稀释 5-10 倍。将盖玻片盖在细胞计数板上，随后利用微量加样器，取 10ul 稀释细胞悬液，加入细胞计数板，于倒置显微镜下计数。将四大象限中的细胞数相加，除以 4，数值意味每毫升细胞数大约在 10^4 个。根据计数结果稀释细胞悬液，再计数确认，调整至终浓度为 1-2*10^5 个/ml。

准备 96 孔板，将浓度一致的细胞悬液以每孔 100ul 的量加入 96 孔板中。每一种细胞需要 6 个复孔。混匀之后加样，需要在短时间内一次性加完，因为细胞会随着时间持续沉降，因此需要反复多次混匀细胞悬液，确保每孔细胞密度及数量保持一致。本实验选取约 2*10^4 个细胞每孔。放于 37℃ 5% CO2 培养箱中。次日拿出，依照 1μg/ml、5μg/ml、10μg/ml、15μg/ml、20μg/ml 五种浓度梯度，在 96 孔板中各组细胞中依据浓度梯度加入嘌呤霉素，需要有空白。放于 37℃ 5% CO2 培养箱中培养 48 小时。后于每一孔中加入 10ul MTT 溶液，再次放于 37℃，5%CO2 培养箱中孵育 2 小时。

两小时后取出 96 孔板，我们选择多层滤纸平放于水平桌面上，将 96 孔板打开，轻轻倒扣在滤纸上，是上层溶液缓缓被滤纸吸附走，以防 MTT 溶液与细胞形成的甲臜络合物丢失。随后每一个孔中加入 150ul DMSO 溶液，放在室温摇床上低速摇晃 30 分钟使甲臜结晶充分溶解，注意需要避光溶解。之后使用酶标仪在 OD 值 490nm 检测各孔吸光度。选取吸光度最低的实验组中嘌呤霉素最低浓度为最佳浓度。本实验中最佳浓度为 10μg/ml。

（6）配置 10μg/ml 嘌呤霉素的培养基

穿戴防护，将细胞培养瓶中细胞原有培养基去除，使用 PBS 溶液冲洗两遍，加入配置 10μg/ml 嘌呤霉素的培养基；将细胞放于 37℃，5%CO2 培养箱中培养；每 48 小时换一次培养基，培养基使用 10μg/ml 嘌呤霉素的培养基。2-3 次换液以后剩下的为稳定细胞系；

之后通过 Western Blot 或 qPCR 来检测细胞系敲低 METTL3 效果。

2.1.3.2 细胞四甲基偶氮唑 MTT 比色法

（1）准备细胞培养耗材，96 孔板，微量加样器，细胞计数板等于放入无菌操作台中紫外线照射消毒杀菌。配置四甲基偶氮唑（MTT）溶液，使用 PBS 溶液或是生理盐水做溶剂，配置成为终浓度 5mg/ml。

（2）选择处于对数生长期的细胞，依照细胞传代方式，去除原有旧培养基，使

用无菌 PBS 溶液清洗三次。使用胰酶消化细胞，待贴壁细胞消化为细胞悬液，利用含有胎牛血清的完全培养基终止胰酶消化。将细胞悬液移至一次性无菌离心管中，以室温，1000rpm，3 分钟离心细胞。离心之后去掉上层液体，加入 3ml 完全培养基，重悬细胞，使其成文均匀的细胞悬液。

（3）用一新的无菌离心管取 1ml 细胞悬液稀释 5-10 倍。将盖玻片盖在细胞计数板上，随后利用微量加样器，取 10ul 稀释细胞悬液，加入细胞计数板，于倒置显微镜下计数。将四大象限中的细胞数相加，除以 4，数值意味每毫升细胞数大约在 10^4 个。根据计数结果稀释细胞悬液，再计数确认，调整至终浓度为 4-5*10^4 个/ml。要确保各个实验用细胞系稀释的细胞悬液终浓度统一。

（4）准备 96 孔板，将浓度一致的细胞悬液以每孔 100ul 的量加入 96 孔板中。每一种细胞需要 6 个复孔。混匀之后加样，需要在短时间内一次性加完，因为细胞会随着时间持续沉降，因此需要反复多次混匀细胞悬液，确保每孔细胞密度及数量保持一致。本实验选取约 2000 个细胞每孔。之后需要在周围边缘等空白孔加入等体积无菌 PBS 溶液。以同样方式加样大约 5-6 块 96 孔板。每次加样需要重新混匀细胞悬液。

（5）随后 96 孔板放入 37℃，5%CO2 培养箱中孵育。每隔 24 小时取出一块 96 孔板。于每一孔中加入 10ul MTT 溶液，再次放于 37℃，5%CO2 培养箱中孵育 2 小时。

（6）两小时后取出 96 孔板，我们选择多层滤纸平放于水平桌面上，将 96 孔板打开，轻轻倒扣在滤纸上，是上层溶液缓缓被滤纸吸附走，以防 MTT 溶液与细胞形成的甲臜络合物丢失。随后每一个孔中加入 150ul DMSO 溶液，放在室温摇床上低速摇晃 30 分钟使甲臜结晶充分溶解，注意需要避光溶解。之后使用酶标仪在 OD 值 490nm 检测各孔吸光度，其中需要有加入 DMSO 的空白孔作为调零孔。记录数值。

（7）之后每 24 小时取出一个 96 孔板进行上述操作，记录各板 OD 值，最终统一计算。

2.1.3.3 细胞集落形成实验

（1）按照细胞培养方式准备试剂及超净台。需要准备细胞计数板，微量加样器，六孔板等。

选取生长良好的对数生长期的细胞，依照细胞传代方式，去除原有旧培养基，

使用无菌 PBS 溶液清洗三次。使用胰酶消化细胞，待贴壁细胞消化为细胞悬液，用胰蛋白酶吹打成单个细胞，利用含有胎牛血清的完全培养基终止胰酶消化。将细胞悬液移至一次性无菌离心管中，以室温，1000rpm，3 分钟离心细胞。

（2）离心之后去掉上层液体，加入 3ml 完全培养基，重悬细胞，使其成文均匀的细胞悬液。

（3）用一新的无菌离心管取 0.5ml 细胞悬液稀释 20 倍。将盖玻片盖在细胞计数板上，随后利用微量加样器，取 10ul 稀释细胞悬液，加入细胞计数板，于倒置显微镜下计数。将四大象限中的细胞数相加，除以 4，数值意味每毫升细胞数大约在 10^4 个。根据计数结果稀释细胞悬液，再计数确认，大约调整至终浓度为 1*10^4 个/ml。

（4）准备六孔板，每一孔选择加入 500 个细胞。依据细胞终浓度加入具体体积的细胞悬液。后在每一孔中加入 2ml 10%FBS 细胞培养基，放于 37℃，5%CO2 培养箱中培养。

（5）大约每 2-3 天进行一次细胞换液，第一次可以 4-5 天换液。具体可以观察培养基情况做决定。可在倒置显微镜下观察细胞集落形成情况。一般培养 14 天。取出培养满意的六孔板，移除原有旧培养基，使用 PBS 溶液小心清去培养基，每次三分钟，洗三次。每孔弃去 PBS 溶液，加入 4℃预冷甲醛溶液 1ml，放置在 4℃冰箱中 15 分钟。

（6）每孔弃去甲醛溶液，使用 PBS 溶液小心清去培养基，每次三分钟，洗三次。弃去 PBS 溶液，每孔加入 1 ml 0.1%的结晶紫溶液进行染色，于常温下静置染色 15 分钟。随后丢弃结晶紫溶液，静置于自来水中洗去结晶紫溶液。放于通风橱中晾干。

2.2 结果

2.2.1 慢病毒敲低前列腺癌中 METTL3 效率

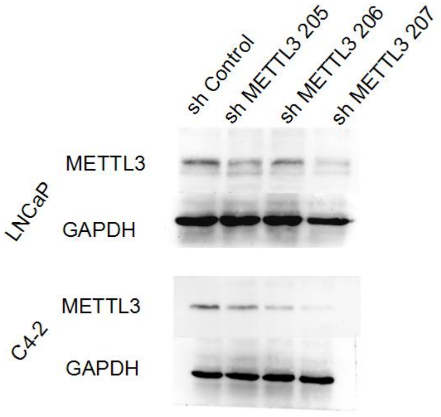

图 2.1

图 2.1 通过慢病毒转染敲低 LNCaP 和 C4-2 细胞系中的 METTL3 水平。建立稳定细胞系后通过 Western Blot 验证细胞 METTL3 敲低效果。LNCaP 和 C4-2 的 Sh METTL3 205 及 Sh METTL3 207 敲低效率高。

2.2.2 下调前列腺癌细胞 METTL3 表达，前列腺癌细胞的增殖能力受到影响。

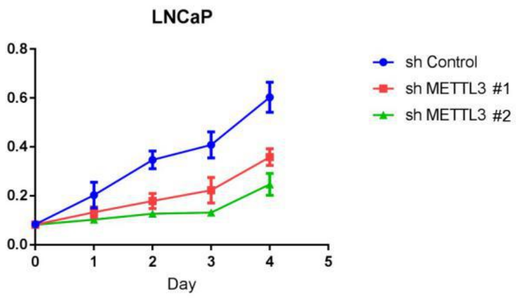

图 2.2

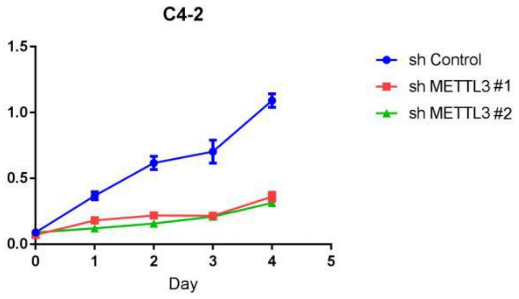

图 2.3

图 2.2 以及图 2.3 分别是稳定敲低 METTL3 的 LNCaP 和 C4-2 细胞利用 MTT 实验验证敲低 METTL3 对细胞增殖能力的影响。用经过 shRNA 敲减 METTL3 的 LNCaP 细胞，与未敲减 METTL3 的 LNCaP 细胞行 MTT 细胞增殖实验。图 2.2 蓝色表示未敲减 METTL3 的 LNCaP 细胞生长情况，红色及绿色线分别表示两个经过 shRNA 敲减 METTL3 的 LNCaP 细胞生长情况。在四天的生长情况下，未敲减 METTL3 的 LNCaP 细胞生长情况与经过 shRNA 敲减 METTL3 的 LNCaP 细胞生长情况有着明显的差异（$p<0.05$）。且未敲减 METTL3 的 LNCaP 细胞比经过 shRNA 敲减 METTL3 的 LNCaP 细胞增值能力更好。同样在图 2.3 中蓝色

表示未敲减 METTL3 的 C4-2 细胞生长情况，红色及绿色线分别表示两个经过 shRNA 敲减 METTL3 的 C4-2 细胞生长情况。未敲减 METTL3 的 C4-2 细胞增殖比经过 shRNA 敲减 METTL3 的 C4-2 细胞增殖情况有着明显的差异（$p<0.05$）。在前列腺癌细胞中敲低 METTL3 会导致细胞增殖能力减弱。

2.2.3 下调前列腺癌细胞 METTL3 表达,前列腺癌细胞的集落形成能力受到影响。

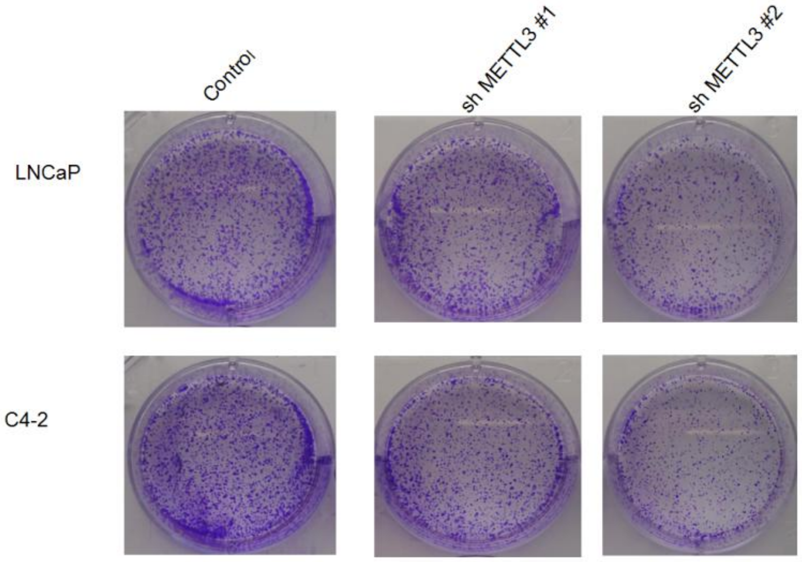

图 2.4

图2.4是用经过shRNA敲减METTL3 的LNCaP细胞，与未敲减METTL3的lncap细胞行细胞克隆形成实验。第一排依次是未敲减 METTL3 的 LNCaP，经过敲减 METTL3 的 LNCaP shMETTL3 #1 和#2。第二排依次是未敲减 METTL3 的 C4-2，经过敲减 METTL3 的 C4-2shMETTL3 #1 和#2，在培养两周后的生长情况，与未敲减 METTL3 的细胞相比，经过 shRNA 敲减 METTL3 的细胞增殖能力明显下降。

2.3 讨论

通过前期实验，我们验证了 METTL3 在 HSPC 中与良性前列腺病变组织比较有着更高的表达。而后通过 q-PCR 实验以及 Western Blot 实验验证了各前列腺癌细胞中 METTL3 的 RNA 水平表达情况以及蛋白水平表达情况。我们验证的所

有前列腺癌细胞系均要明显高出 BPH1 细胞系。在之前已经发表的结论中我们发现在许多肿瘤中，METTL3 起到促进肿瘤增殖的功能，例如 METTL3 下调明显增强了胶质母细胞瘤细胞的干细胞生长和自我更新能力，促进肿瘤的发展，而 METTL3 的过表达则相反。METTL3 在急性髓系白血病(AML)细胞和原发性白血病细胞中高度表达。后 shRNA 下调 METTL3 后，AML 细胞中 m6A 水平随细胞分化和凋亡的增加而下降[15]。

我们通过沉默目的基因 METTL3 验证 METTL3 在前列腺癌发生发展中的作用。基因沉默方式主要有小干扰 RNA(siRNA)，慢病毒载体转运发卡 RNA（sh RNA）以及新兴的 CRISPR/Cas9 技术。其中 siRNA，通过特定转染试剂利用电化学原理细胞膜穿孔，让其进入细胞，这样的方式直接将 siRNA 序列转染至细胞，通常作用代数短，细胞在数代内，其沉默作用会逐步消失，不能获得稳定表达敲低的细胞系。而通过慢病毒转染细胞，干扰目的基因其转染效率较高，对细胞的毒性更低，能够获得稳定的敲减细胞系。因此我们选择构建慢病毒携带 shRNA 进行干扰沉默目的基因。

在本实验，由北京合生基因生物科技有限公司的协助合成 3 条 sh RNA。然后我们选取 LNCaP 细胞系及 C4-2 细胞系通过慢病毒 shRNA 敲减前列腺癌细胞系中的 METTL3。通过转染后筛选，利用 Western Blot 验证建立了 LNCaP sh-METTL3#1 和#2，以及 C4-2 sh-METTL3#1 和#2。通过 MTT 细胞增殖实验，及细胞克隆形成实验验证细胞增殖能力。实验结果显示敲低 METTL3 前列腺癌细胞与未敲低前列腺癌细胞增殖能力有显著差异，敲低 METTL3 前列腺癌细胞增值能力弱于未敲低前列腺癌细胞。表明 METTL3 在前列腺癌中会减弱前列腺癌细胞的增殖。METTL3 在前列腺癌的发生发展中的潜在机制。下一步是研究 METTL3 对于前列腺癌发生发展的机制，及能否通过抑制他的表达或者抑制他的作用来到达检测预防前列腺癌的发生，或抑制其进一步发展。

2.4 小结

通过慢病毒转染 shRNA 可建立稳定敲低 METTL3 的前列腺癌细胞系。METTL3 的降低会影响 HSPC 细胞系的细胞增殖能力，敲低 METTL3 会减弱细胞增殖能力。METTL3 在前列腺癌中起到了增强细胞增殖的作用。

三、METTL3 在前列腺癌中介导 RNA m6A 甲基化水平改变影响 Hippo 信号通路

应用 MeRIP-seq 高通量测序检测前列腺癌组织基因 m6A 丰度以及 mRNA。后通过 MeRIP-qPCR 验证相应基因改变;并且利用 Western Blot 验证 YAP1,c-MYC 等相应基因。

3.1 对象和方法

3.1.1 研究对象

3.1.1.1 前列腺组织

上文中收集患者组织标本中取 5 例良性前列腺病变组织，5 例前列腺癌患者肿瘤组织样本。患者对肿瘤组织样本收集处理均表示知情理解，且通过医院伦理委员会批准。

3.1.1.2 前列腺癌细胞

选用 LNCaP 人前列腺 癌细胞系、C4-2 人前列腺癌细胞系，来自于美国组织培养中心（ATCC）以及利用慢病毒敲减 METTL3 建立的 LNCaP 及 C4-2 细胞系。

3.1.2 研究方法

3.1.2.1 实验试剂

名称	来源
RPMI Media 1640 细胞培养基	美国 Gibco 公司
Gibco 热灭活胎牛血清（FBS）	美国 Gibco 公司
Penicillin-Streptomycin 双抗	美国 Gibco 公司
Trypsin-EDTA (0.05%)（胰酶）	美国 Gibco 公司
二甲基亚砜（DMSO）	北京索莱宝科技有限公司

磷酸盐缓冲液（PBS）	北京索莱宝科技有限公司
Trizol	美国 Invitrogen 公司
三氯甲烷（氯仿）	天津市光复科技发展有限公司
异丙醇	天津市光复科技发展有限公司
无水乙醇	苏州海百化工有限责任公司
RIPA 裂解液	美国 Thermo Fisher Scientific 公司
PMSF 蛋白酶抑制剂	美国 Thermo Fisher Scientific 公司
Bradford(考马斯亮蓝)缓冲液	美国伯乐公司
Loading buffer 缓冲液	北京索来宝试剂有限公司
Western-blot 蛋白 marker	北京索来宝试剂有限公司
SDS-PAGE 凝胶制备试剂盒	北京索来宝试剂有限公司
30%Acr/Bis(29:1)	北京索来宝试剂有限公司
1M Tris-HCl(PH6.8)	北京索来宝试剂有限公司
1.5 Tris-HCl(PH8.8)	北京索来宝试剂有限公司
10%SDS	北京索来宝试剂有限公司
10%PAGE 胶凝固剂	北京索来宝试剂有限公司
PAGE 胶促凝剂	北京索来宝试剂有限公司
ddH_2O	天津市泌尿外科研究所
甘氨酸	美国 Sigma 公司
三羟甲基氨基甲烷（Tris）	美国 Sigma 公司

Tween-20	美国 Sigma 公司
十二烷基磺酸钠	美国 Sigma 公司
脱脂奶粉	美国 Sigma 公司
RevertAid First Strand cDNA Synthesis Kit	美国 Thermo Fisher Scientific 公司
Taq MasterMix	美国 Thermo Fisher Scientific 公司
兔抗人 METTL3 单克隆抗体	abcam corporation
兔抗人 m6A 单克隆抗体	德国默克集团
鼠抗人 GAPDH 单克隆抗体	北京中杉金桥生物科技技术有限公司
山羊抗兔二抗	北京中杉金桥生物科技技术有限公司
山羊抗鼠二抗	北京中杉金桥生物科技技术有限公司
硝酸纤维素膜（PVDF 膜）	美国 Amresco 公司
ECL 化学发光超敏显示试剂盒	美国 Thermo Fisher Scientific 公司
Magna RIP RNA-Binding Protein Immunoprecipitation Kit	上海浩然生物技术有限公司

3.1.2.2 实验仪器

名称	来源
10cm 细胞培养皿	美国 Thermo Fisher Scientific 公司
15cm 细胞培养皿	美国 Thermo Fisher Scientific 公司

15ml 离心管	美国 Thermo Fisher Scientific 公司
50ml 离心管	美国 Thermo Fisher Scientific 公司
细胞冻存管	美国 Thermo Fisher Scientific 公司
090-135.001 型倒置相差显微镜	Germany Leica Corporation
YG-857 型超净工作台	江苏苏州长桥净化设备厂
L530 型台式高速离心机	湖南长沙湘仪离心机仪器有限公司
HW0301T 型 CO_2 细胞培养箱	美国 Thermo Fisher Scientific 公司
DK600 型电热恒温水浴箱	上海实验器械总公司
BCD268K 型普通冰箱	青岛海尔集团
UTL 型超低温冰箱	美国 Thermo Fisher Scientific 公司
SS325 型高压蒸汽灭菌锅	日本 tomy 公司
FM1200E 制冰机	苏州星琦电机有限公司
10μL 微量移液器	德国 Eppendorf 公司
100μL 微量移液器	德国 Eppendorf 公司
1000μL 微量移液器	德国 Eppendorf 公司
通风操作台	拉贝尔仪表器厂
5810R 型低温超速离心机	德国 Eppendorf 公司
干式金属浴恒温仪	杭州佑宁科技有限公司
7900HT 荧光定量 PCR 仪	美国应用系统生物科技有限公司
WD9405B 型水平摇床	北京市六一仪器厂

全自动酶标仪	美国 Thermo Fisher Scientific 公司
分光光度计	日本 Takara 生物科技公司
稳压 SDS-聚丙烯酰胺凝胶电泳仪	美国伯乐公司
Western Blot 转膜仪	美国伯乐公司
Tanon 全自动化学发光图像处理系统	上海市天能科技有限责任公司
BP-II 型微量药物天平	上海第二医用激光仪器厂
旋转培养器	海门市其林贝儿仪器制造有限公司
液氮罐	美国 Thermo Fisher Scientific 公司

3.1.2.3 斑点印迹杂交实验（Dot Blot）

（1）首先使用 TRIZOL 试剂提取细胞 RNA 选择生长良好，90%丰度的细胞，移除原有培养基，使用 PBS 轻柔冲洗 3 次，吸尽 PBS，加入 1ml TRIZOL，在室温中放于摇床 5-10 分钟，使其充分裂解。

（2）将裂解了细胞的 TRIZOL 试剂转移至 1.5ml EP 管中，加入 0.2ml 氯仿，后盖上盖子，剧烈摇晃，充分混合，放于 4℃冰箱静置 15 分钟。后放入 4℃离心机中，以 12000g 的条件，离心 15 分钟。

（3）小心取出 EP 管，勿倾倒混合，吸取上层透明水相上清 0.5ml 移至新的 1.5ml EP 管中。加入 0.5ml 预冷异丙醇，并于 4℃冰箱静置 10 分钟。后放入 4℃离心机中，以 12000g 的条件，离心 10 分钟。

（4）后去除 EP 管中上清液，尽量吸尽。加入 1ml 新配 75%乙醇（RNAse free），简单地震荡或者吹打。后放入 4℃离心机中，以 7500g 的条件，离心 5 分钟。此步骤可进行两次。后吸尽上清，在空气中干燥 5-10 分钟。

（5）之后用 20ul 无酶水溶解。再置于 60 摄氏度金属浴加热 15 分钟。取 2ul 用于分光光度计检测其吸光度。测算 RNA 浓度，并记录。

（6）然后准备硝酸纤维素膜 NC 膜，EP 管，及紫外交联仪。使用无酶水（RNAse free）将样本 RNA 稀释至 100ng/ul 并放入 95 摄氏度恒温金属浴中加入 5 分钟，

随后立即放置在冰上。将 RNA 滴在 NC 膜上，并且做上标记以示正反。放进紫外交联仪中使用 254nm 交联 5 分钟。

（7）配置 PBS-T，在 1L 0.01M PBS 缓冲液中加入 Tween-20 1ml。用电子天平称量脱脂奶粉 2.5g，用 PBST 缓冲液溶解，配置成 5%脱脂奶粉封闭液。将经过紫外交联的 nc 膜放入盛有 PBST 溶液的干净托盘中洗涤。在室温下，使用摇床轻轻摇晃洗涤三次，每次 5 分钟。将未结合的 RNA 洗掉。随后丢弃洗涤 PBST 缓冲液，加入使用 PBST 为溶液的 5%脱脂奶封闭液，在室温中孵育，封闭一小时。

（8）封闭完成后，再次使用 PBST 缓冲液洗涤 NC 膜三次，每次 5 分钟。将抗 m6a 抗体按照说明使用 PBST 缓冲液按照 1:500 稀释，配置成终浓度为 2ug/ml 的工作液。将洗涤过的 NC 膜浸一抗工作液中，在 4℃冰箱中摇床上摇晃孵育过夜。

（9）次日，回收一抗工作液，并将 NC 膜浸入 PBST 缓冲液洗涤，每一次 5 分钟洗涤三次，除去未结合的一抗。随后使用二抗孵育，在室温中，摇床轻轻摇晃 1 小时。

（10）随后回收二抗，PBST 缓冲液洗膜三次，每一次 10 分钟。打开曝光机，预冷至-40 摄氏度。新配置化学发光显影液（ECL），A 液 B 液按照 1:1 避光混合，滴在膜上，放入曝光机中显影曝光。

3.1.2.4 m6A RNA 甲基化测序

测序实验由上海云序生物科技有限公司协助完成

（1）样本收集制备

（2）mRNA 样品提取；由云序科技公司提取组织总 RNA，然后用 Oligo-dT 磁珠将总 RNA 中对带有 polyA 尾的 mRNA 富集出来；

（3）将提取的 RNA 样品进行片段化；利用超声将 mRNA 打碎成 100kb-200kb 大小的核酸片段；

（4)将得到的RNA片段利用特异性识别mRNA上m6A甲基化(m⁶A)的抗体进行免疫富集

（5）对富集后含有 m6A 的 mRNA 片段洗脱后进行沉淀纯化；

（6）将所得纯化后的 RNA 反转录成 cDNA，并进行 DNA 末端修复、接头连接

和 PCR 扩增，完成 mRNA 甲基化文库的构建；将完成的文库利用分析测序文库的高通量测序平台进行测序。

3.1.2.5 筛选差异 m6A 方法

利用生物信息学方法筛选差异 m6A 表达，以 P 值小于 0．05，改变倍数大于 2 倍为筛选条件，筛选出原发性激素敏感性前列腺癌与良性前列腺病变组织中发生 m6A 丰度上调的 RNA，同样条件筛选 mRNA 水平改变基因。再进行京都基因与基因组百科全书通路富集分析(Kyoto Encyclopedia of Genes and Genomes，KEGG)，以了解差异基因可能功能。

3.1.2.6 RNA m6A 结合蛋白免疫沉淀（MeRIP）

使用 Magna RIP Kit 试剂盒

（1） 制备细胞裂解液

裂解液制备，准备冰板，在冰上制备新鲜裂解液，按照说明，每一个样本需要混合 100ul RIP Lysis Buffer、0.5ul Protense inhibiter、0.25ul RNAse inhibitor，混合成使用的细胞裂解液。

选择生长良好，90%丰度的细胞，移除原有培养基，使用冰 PBS 缓冲液轻柔冲洗 2 次，除尽原有培养基，加入冷 PBS 缓冲液 1-2ml，使用一次性塑料细胞刮刷将细胞刮下来，全部收集至干净 EP 管中。放入预冷的 4 摄氏度高速离心机中，设定 1500rpm，离心 5 分钟。随后丢弃上清，收集下层细胞。将之前配置的 RIP 细胞裂解液，按照与 EP 管中下层细胞同体积比例加入 EP 管，使用移液枪吹打重悬细胞。吹打至均匀后放在冰上静置 5 分钟。随后分装细胞裂解液至新 EP 管中，每一管 200ul，可储存于-80℃冰箱。

（2）磁珠的准备

使用 RIP Wash Buffer 重悬磁珠，第一次按照说明要求加入 RIP Wash Buffer 重悬磁珠。随后按照需求，使用微量加样器吸取 50ul 重悬后的均匀磁珠悬液到新的 EP 管中。然后加入 500ul RIP Wash Buffer，利用涡旋器涡旋震荡，充分混匀并清洗磁珠。之后将 EP 管置于磁力将上，可左右旋转，使磁珠完全被磁力架捕获，随后去除上清。按照上述步骤加入 500ulRIP Wash Buffer 再次清洗磁珠一遍。然后去除 RIP Wash Buffer，重新加入 100ul RIP Wash Buffer 并利用涡旋震荡重悬磁珠。在 EP 管中加入抗 m6A 抗体 1ul。将 EP 管放置在旋转培养器中放于室温孵育 1 小时。孵育完成后将 EP 管放置在磁力架上，去除上清。随后加入

500ul RIP Wash Buffer，利用涡旋器涡旋震荡，充分混匀并清洗磁珠。之后将EP管置于磁力将上，去除上清。按照上述步骤加入500ulRIP Wash Buffer再次清洗磁珠一遍。然后去除RIP Wash Buffer，重新加入500ul RIP Wash Buffer并利用涡旋震荡重悬磁珠，之后放在冰上。

（3）RNA结合蛋白免疫沉淀

准备RIP Immunoprecipitation Buffer，然后将上一步中孵育过m6A抗体的磁珠EP管放磁力架上，清除上清，加入900ul RIP Immunoprecipitation Buffer。将之前存放于-80℃的制备好的细胞裂解液迅速解冻，然后放入4℃高速离心机，按照14,000rpm，10min离心。随后使用微量加样器吸100μl上清液加入磁珠-抗体复合物中，得到总体积1ml的反应混合物，随后放置在4℃冰箱，使用旋转培养器孵育过夜。次日取出先简单离心，使管盖上不留液体，然后放置在磁力架上，丢弃液体。使用微量加样器吸取50ul重悬后的均匀磁珠悬液到新的EP管中。然后加入500ul RIP Wash Buffer，利用涡旋器涡旋震荡，充分混匀，后将EP管置于磁力将上，使磁珠完全被磁力架捕获，随后去除上清。按照上述步骤加入500ulRIP Wash Buffer重复清洗6次。

（4）RNA纯化

准备Proteinase K Buffer，溶解，然后使用微量加样器抽取150ul Proteinase K Buffer加入上述磁珠-抗体混合物EP管中。利用涡旋器震荡混匀重悬磁珠，随后放入55℃恒温金属浴孵育30分钟。孵育完之后，将EP管置于磁力架上，用微量加样器吸取上清，加入一新的EP管中。然后再加入250ul RIP Wash Buffer和400ul苯酚：氯仿：异戊醇试剂，涡旋震荡15s，在高速离心机中按照14000rpm离心10分钟。完成后用微量加样器，小心吸取350ul上层水相液体，移至一个新的EP管中。然后在管中加入400μl氯仿，涡旋震荡15s，在高速离心机中室温下14,000rpm离心10分钟。完成后用微量加样器，小心吸取300ul上层水相液体，移至一个新的EP管中。再用微量加样器依次加入50ul Salt SolutionⅠ，15ul Salt SolutionⅡ，5ul Precipitate Enhancer，850μl无水乙醇（无RNAse），混匀放置在-80℃冰箱中保持1h至过夜。取出后于4℃高速离心机以14,000rpm离心30min，然后小心弃掉上清。加入80%乙醇1ml，简单地震荡或者吹打。后放入4℃高速离心机中，以14,000rpm，离心15分钟。小心去除上清，在空气中晾干。随后使用20ul无酶水溶解，样本可置于-80℃保存。

3.1.2.7 MeRIP-pcr

将上述 MeRIP 实验取得的 RNA 进行 RT-qPCR。具体步骤见上述相应实验方法。

3.2 结果

3.2.1 敲低 METTL3 后细胞 RNA m6a 甲基化修饰水平改变

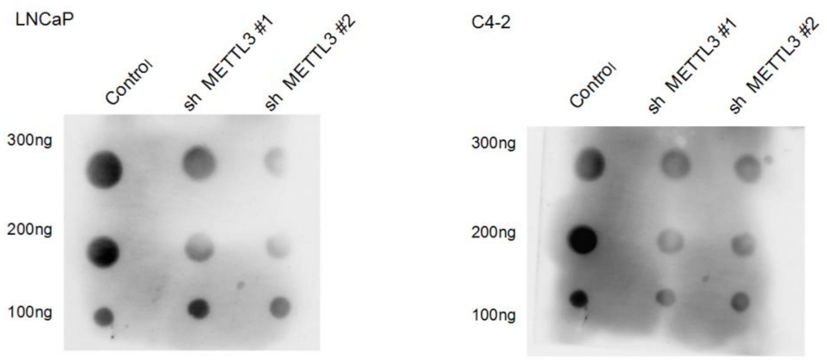

图 3.1

图 3.1 利用 LNCaP 和 C4-2 细胞系通过敲减 METTL3 表达，比较 METTL3 含量降低对细胞 RNA m6A 甲基化修饰水平的影响。通过 Dot Blotting 实验来验证细胞 RNA m6A 甲基化修饰水平改变情况。实验结果如图 3.1 显示由上自下每一行分别表示样本 RNA 含量，分别为 300ng，200ng，100ng。三列分别是未敲低 METTL3 的细胞系，以及敲减 METTL3 的 sh METTL3 #1 和#2。实验结果表明，改变 METTL3 表达后，细胞 RNA m6A 甲基化修饰水平会随之改变，敲低 METTL3 表达水平后，细胞 RNA m6A 甲基化修饰水平明显降低。

3.2.2METTL3 的敲低会影响 c-MYC 的 mRNA 蛋白表达下降

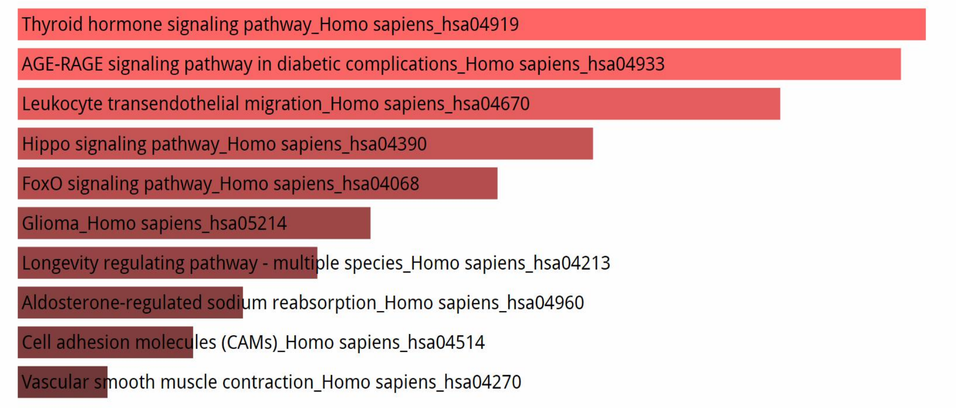

图 3.2

通过分析前列腺癌组织及良性前列腺病变组织 mRNA 测序数据，以前列腺癌组比良性前列腺病变组，筛选出 mRNA 差异表达基因。通过分析前列腺癌组织及良性前列腺病变组织 mRNA m6A 甲基化修饰 测序数据，以前列腺癌组比良性前列腺病变组，筛选 mRNA m6A 甲基化修饰差异表达基因。选取 mRNA m6A 甲基化修饰水平下调的差异基因与 mRNA 水平改变的差异基因取交集。将交集基因利用生物信息学方法进行 KEGG 富集。图 3.2 示 KEGG 富集结果。

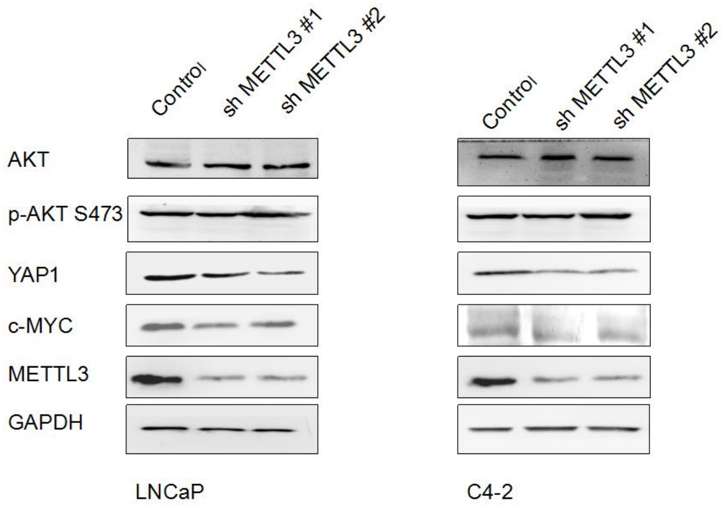

图 3.3

在 Hippo 通路中改变蛋白中，我们利用 WESTERN 实验，检验在未敲低

METTL3 的前列腺癌细胞系中与经过 sh RNA 敲减 METTL3 的前列腺癌细胞系，AKT,p-AKT,YAP,c-MYC 等蛋白的表达含量。实验结果如图 3.3 示 ATK，p-AKT,蛋白在敲低 METTL3 蛋白表达及未敲低 METTL3 蛋白组无明显改变，YAP1，及 c-MYC 蛋白在敲低 METTL3 后蛋白表达水平改变。在敲低 METTL3 组比较未敲低 METTL3 组中 YAP1 及 c-MYC 蛋白表达有明显减少。

3.2.3METTL3 的敲低会影响 c-MYC 的 mRNA m6a 水平的降低导致其 mRNA 表达水平改变

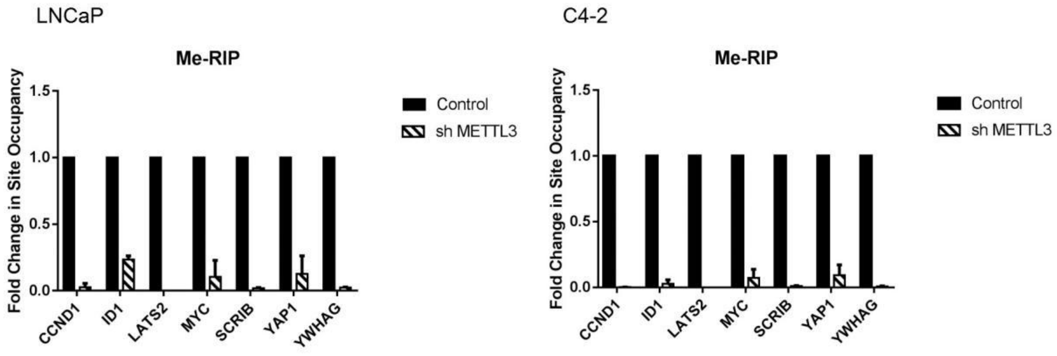

图 3.4

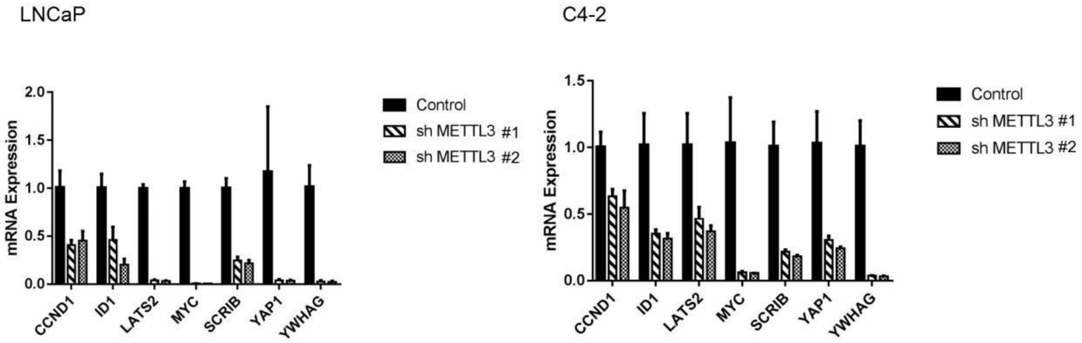

图 3.5

为了验证 c-MYC 蛋白降低的机制，我们利用实验 RIP-qPCR，首先选取经过 KEGG 富集的基因，设计引物。利用 m6A 一抗抗体进行 RIP 实验，得到存在 mRNA m6A 甲基化修饰的基因。通过 qPCR 实验验证比较敲减 METTL3 与未敲减 METTL3 富集的 mRNA m6A 甲基化基因水平改变。图 3.4 所示：敲减

METTL3 后的细胞富集基因 mRNA m6A 甲基化水平明显下调。而后通过 qPCR 实验验证敲减 METTL3 前后细胞中富集基因 mRNA 水平变化。图 3.5 显示富集基因在敲减 METTL3 细胞中 mRNA 水平均有程度的改变，敲减 METTL3 细胞比较未敲减 METTL3 细胞富集基因 mRNA 表达水平均明显下调。

3.3 讨论

之前的报道已经验证了 METTL3 是哺乳动物细胞中 m6A 甲基化转移酶复合物的重要组成部分。实验证明在 HeLa 细胞中敲低 METTL3 的表达，会使细胞总 m6A 水平明显下降。并且已经证实 METTL3 在多种肿瘤中发挥了作用，如非小细胞肺癌（NSCLC），肝细胞癌（HCC），急性髓系白血病(AML)等肿瘤中 METTL3 的表达水平增高介导了细胞中 m6A 水平的增加，而在宫颈癌中 m6A 水平则是随着 METTL3 的表达水平降低而降低。而在激素敏感型前列腺癌中，并没有相关机制研究。本研究中我们通过建立的稳定敲低 METTL3 的前列腺癌细胞系，利用 m6A 特异性抗体，通过 Dot Blot 实验验证了敲低 METTL3 后前列腺癌细胞 m6A 水平的改变。我们证实了在前列腺癌细胞中敲低 METTL3 表达后，细胞中 m6A 水平会有明显下降。

运用近年兴起的高通量 m6A-seq 这一测序技术，结合临床，通过检测前列腺癌症患者肿瘤组织，与良性前列腺病变组织。通过 m6A 测序结合 mRNA 测序我们找出 mRNA 水平有差异化同时 mRNA m6A 甲基化差异的基因。由于经过前期文献及实验，METTL3 是一种甲基转移酶，介导 RNA 上 m6A 甲基化修饰。METTL3 敲低的情况下，前列腺癌细胞中 RNA m6A 水平是明显减低的。另外在人前列腺癌组织，及人前列腺癌细胞系中 METTL3 在蛋白及 RNA 水平上与良性前列腺病变组织或者细胞中 METTL3 相比有着明显升高。前期实验证明，在结合试验及文献，我们认为在前列腺癌组织中 METTL3 的增高引起 RNA m6A 水平增高。因此我们选取 RNA m6A 水平增高基因，与 mRNA 水平改变基因取交集。因为我们并不清楚基因的 RNA m6A 水平的增高在前列腺癌中会引起基因表达水平怎样的改变。之后我么将这个基因交集运用 Irich 分析工具进行 KEGG 通路的富集，得到结构图 3.2。我们选取 Hippo 通路中相关蛋白进行验证。

研究表明 Hippo-YAP 信号通路的异常活化可导致肿瘤发生发展，YAP 在许

多实体肿瘤如肝癌、肺癌、卵巢癌中均表达升高[35,36]。该通路中 YAP 主要受到上游憐酸化调节[34]，促使 YAP 从胞核移至胞质，从而使 YAP 活性丧失；YAP 磯酸化后相关基因表达受到抑制，包括增殖相关基因 Ki67、c-MYC、Cyclin D 和 SOX4 等。Hippo-YAP 通路的失调主要包括基因突变、杂合性缺失、基因易位和表观遗传学改变；其中一些生长调节通路，如 RAS 信号通路，可抑制该信号通路上游因子，间接激活 YAP 功能，参与 YAP 通路调控。

根据我们前期的文献查找以及实验验证，我们猜想 METTL3 在前列腺癌中水平的升高，改变了 Hippo-YAP 信号通路中基因的表观遗传学修饰导致了其异常的激活。从而影响下游基因的异常表达，导致前列腺癌细胞的增殖能力的改变。

通过 western 我们验证发现敲低 METTL3 前列腺癌细胞对比野生型前列腺癌细胞，AKT，及磷酸化 AKT 并无明显改变，而 YAP1 以及 c-MYC 表达水平降低。为此我们考虑 METLL3 是通过了调节癌基因 c-MYC 而使得前列腺癌发生发展，在敲低 METTL3 后能够抑制前列腺癌细胞的增殖能力。

癌基因 c-MYC 在细胞增殖与分化、细胞周期以及细胞凋亡等过程中发挥这重要作用[37]。正常情况下，c-MYC 的表达受到严格调控，当生长因子等刺激时表达会升高。当染色体易位或信号通路基因异常激活等情况发 生的时后，c-MYC 会在不依赖于生长因子刺激的时候扩增， 导致异常细胞增殖和肿瘤的发生 Zeng 研究团队 通过免疫组化实验，检测了 81 例前列 腺癌患者的组织 c-MYC 表达水平，证实在前列腺癌中 c-MYC 表达水平与肿瘤进展有正相关性［39］。c-MYC 也是 Hippo-YAP 信号通路的下游基因。

为了进一步探究其之间的关系及机制，我们通过 IGV 软件 分析了富集基因 RNA m6A 甲基化出现的位置及水平。c-MYC 在前列腺癌组织中 RNA m6A 甲基化有着明显的增高，而 YAP1 基因 RNA m6A 甲基化水平并无明显差异。我们观察到 c-MYC 的 mRNA m6A 甲基化修饰在三个外显子上都有存在并且在前列腺癌组织中明显升高，另外在 mRNA 3’端也有明显升高。这可能与他的翻译效率以及 mRNA 的稳定性相关。随后利用 MeRIP 实验富集得到 RNA m6A 甲基化基因。在通过 qPCR 实验来定量比较野生型前列腺癌细胞系 LNCaP 及 C4-2 与敲低 METTL3 后的前列腺癌细胞系中基因表达水平改变。敲低 METTL3 后的前列腺癌细胞系 LNCaP 及 C4-2 与野生型的相比较，c-MYC，YWHAG,CCND1,SCRIB 等基因 RNA m6A 甲基化水平均有明显下降。同时通过 qPCR 比较敲低 METTL3

后的前列腺癌细胞系 LNCaP 及 C4-2 与野生型细胞中，c-MYC，YWHAG,CCND1,SCRIB 等基因 mRNA 水平表达的差异，这些基因在敲低 METTL3 后其 mRNA 水平有不同程度的降低。

3.4 小结

在 HSPC 中 METTL3 介导了 m6A 水平的改变。在高表达 METTL3 的 HSPC 组织中，基因 RNA m6A 水平丰度增加，从而导致了 Hippo-YAP 通路的异常激活，YAP1,c-MYC 等基因的异常表达。在前列腺癌细胞中 METTL3 的降低会使 YAP1、c-MYC、Cyclin D 等基因 m6A 水平明显的降低，从而导致其表达水平的下降。

结论

METTL3 在激素敏感型前列腺癌中异常表达，相比于前列腺良性病变组织有明显升高。通过临床标本以及细胞同时验证了 METTL3 在 HSPC 中的异常表达。通过体外实验，利用慢病毒转染 shRNA 做到干扰前列腺癌细胞中 METTL3，建立稳定敲减 METTL3 的前列腺癌细胞系。验证了 METTL3 在 HSPC 细胞系中影响细胞增殖能力，敲低 METTL3 会减弱细胞增殖能力。

于文献报道 METTL3 是作用在 RNA m6A 的甲基转移酶，我们利用 m6A 特异性抗体，通过 Dot Blot 实验证明，在 HSPC 中,敲减 METTL3 会使细胞 RNA m6A 水平降低。通过高通量 m6A-seq 这一测序技术，对收集的临床标本行高通量测序，来检测 HSPC 和前列腺良性病变组织的差异基因，通过组织测序发现其中 m6A 的丰度显著增加。为此我们利用了 KEGG 信号通路富集分析，发现 METTL3 介导了 HSPC 中 m6A 的改变，进而影响了 Hippo-YAP 通路。通过 Western Blot 实验，我们验证了在前列腺癌细胞中，改变 METTL3 的表达水平，会影响 YAP1 及 c-MYC 的水平改变。通过 MeRIP-qPCR，在前列腺癌细胞中 METTL3 的降低会使 YAP1、c-MYC、Cyclin D 等基因，m6A 水平明显的降低。从而导致其表达水平的下降。通过测序以及体外实验，表明 METTL3 在 HSPC 中表达的升高，导致 Hippo 通路中数个基因的 RNA m6A 水平的升高，使其异常激活。导致下游 c-MYC 基因异常表达，从而增强了前列腺癌细胞的增殖能力，促进了 HSPC 的发生发展。

本研究 METTL3 介导的 m6A 修饰通过调节 Hippo 信号通路是促进 HSPC 的进展。从表观遗传学的角度揭示了前列腺癌的发生发展，为前列腺癌患者的治疗提供一个新的方向。但对于 m6A 在信号通路激活的具体机制未做将进一步研究，有待进一步阐明。

参考文献

[1]SIEGEL RL,MILLER KD,JEMAL A.Cancer statistics, 2019[J].CA Cancer J Clin, 2019,69(1):7-34.

[2]CENTER MM , J EMAL A ,LORTET-TIEULENT J, et al. RNA Intetional variation in prostate cancer incidence and mortality rates[J].European urology, 2012 ,61:1079-1092

[3]CHEN W, ZHENG R, BAADE PD, et al.Cancer statistics in China, 2015[J].CA Cancer J Clin,2016,66(2):115-32.

[4]李鸣，张思维，马建辉等. 中国部分市县前列腺癌发病趋势比较研究[J]. 中华泌尿外科杂志，2009，30: 568- 570

[5]韩苏军，张思维，陈万青等. 中国前列腺癌发病现状和流行趋势分析[J]. 临床肿瘤杂志，2013，18: 330-334

[6]赫捷，陈万青. 2012 中国肿瘤登记年报[J]. 北京:军事医学科学出版社,2012

[7]NG RK, GURDON JB. Epigenetic inheritance of cell differentiation status[J].Cell Cycle,2008, 7: 1173-7

[8]ZHANG LL, WU JX.DNA methylation:an epigenetic mechanism for tumorigenesis[J]. Yi Chuan,2006,28: 880-5

[9]DESROSIERS R, FRIDERICI K, ROTTMAN F. Identification of methylated nucleosides in messenger RNA from Novikoff hepatoma cells[J].Proc Natl Acad Sci U S A. 1974,71(10):3971–5.

[10]ALARCÓN CR, LEE H, GOODARZI H, HALBERG N, et al. N6-methyladenosine marks primary microRNAs for processing[J]. Nature, 2015,519(7544):482–5.

[11]PATIL DP, CHEN CK, PICKERING BF, et al.m6A RNA methylation promotes XIST-mediated transcriptional repression[J].Nature,2016,537(7620):369–73.

[12]CANTARA WA, CRAIN PF, ROZENSKI J, et al. The RNA modifification database, RNAMDB: 2011 update[J].Nucleic Acids Res,2011,39:195–201.

[13]CZERWONIEC A, DUNIN-HORKAWICZ S, PURTA E, et al. MODOMICS: a database of RNA modifification pathways 2008 update[J].Nucleic Acids Res, 2009,37:118–21.

[14]DESROSIERS R, FRIDERICI K, ROTTMAN F. Identification of methylated nucleosides in messenger RNA from Novikoff hepatoma cells[J].Proc Natl Acad Sci USA 1974; 71: 3971-5.

[15]Xia Wu, Lina Sang, Yuping Gong. N6-methyladenine RNA modification and cancers[J]. Am J Cancer Res,2018,8(10):1957-1966.

[16]ARPITA MAITY, BISWADIP DAS. N6-methyladenosine modifification in mRNA: machinery,function and implications for health and diseases[J].FEBS JouRNAl,2016,283: 1607–1630

[17]DOMINISSINI D, MOSHITCH-MOSHKOVITZ S, SCHWARTZ S.Topology of the human and mouse m(6)A RNA methylomes revealed by m(6)A-seq[J]. Nature 2012,485: 201-8

[18]LIU J,et al.A METTL3-METTL14 complex mediates mammalian nuclear RNA N6-adenosine methylation[J].Nature Chem Biol,2014,10:93-95.

[19]LEACH RA, TUCK MT. Expression of the mRNA (N6-adenosine)-methyltransferase S-adenosylL-methionine binding subunit mRNA in cultured cells[J]. Int J Biochem Cell Biol,2001,33:984-999.

[20]PING XL, SUN BF, WANG L.Mammalian WTAP is a regulatory subunit of the RNA N6-methyladenosine methyltransferase[J]. Cell Res,2014,24: 177-189.

[21]JIA G, FU Y, ZHAO X, DAI Q,et al. N6-methyladenosine in nuclear RNA is a major substrate of the obesity-associated FTO[J] .Nat Chem Biol,2012,7: 885-7.

[22]ZHENG G, DAHL JA, NIU Y, et al. ALKBH5 is a mammalian RNA demethylase that impacts RNA metabolism and mouse fertility[J]. Mol Cell,2013,49: 18-29.

[23]WANG X, LU Z, GOMEZ A, et al. N6-methyladenosine-dependent regulation of messenger RNA stability[J]. Nature,2014, 505: 117-20

[24]SHI H, WANG X, LU Z, et al.YTHDF3 facilitates translation and decay of N6-methyladenosine-modified RNA[J]. Cell Res,2017,27: 315.

[25]LI A, CHEN YS, PING XL, YANG X,et al. Cytoplasmic m6A reader YTHDF3 promotes mRNA translation[J]. Cell Res,2017, 27: 444-7.

[26]PATIL DP, CHEN CK, PICKERING BF, et al. M6A RNA methylation promotes XIST-mediated transcriptional repression[J]. Nature,2016,537: 369-73.

[27]XIAO W, ADHIKARI S, DAHAL U,et al. Nuclear m6A reader YTHDC1

regulates mRNA splicing[J]. Mol Cell,2016,61: 507-19.
[28]LIN S, CHOE J, DU P, TRIBOULET R, GREGORY RI. The m(6)A methyltransferase METTL3 promotes translation in human cancer cells[J].Mol Cell,2017,62: 335-45.
[29]DU M, ZHANG Y, MAO Y, MOU J, et al. MiR-33a suppresses proliferation of NSCLC cells via targeting METTL3 mRNA[J]. Biochem Biophys Res Commun,2017,482: 582-9.
[30]VU LP, PICKERING BF, CHENG Y, et al. The N6-methyladenosine (m6A)-forming enzyme METTL3 controls myeloid differentiation of normal hematopoietic and leukemia cells[J]. Nat Med,2017,23: 1369-76.
[31]MA J Z, YANG F, ZHOU C C, et al. METTL14 suppresses the metastatic potential of hepatocellular carcinoma by modulating N6 ‐ methyladenosine ‐ dependent primary MicroRNA processing[J]. Hepatology, 2017, 65(2):529.
[32]WANG X, LI Z, KONG B, SONG C, et al. Reduced m6A mRNA methylation is correlated with theprogression of human cervical cancer[J].Oncotarget,2017,8: 98918-30.
[33]LI X, TANG J, HUANG W, et al. The M6A methyltransferase METTL3: acting as a tumor suppressor in renal cell carcinoma[J]. Oncotarget,2017,8: 96103-16.
[34]AZUCENA RAMOS, FERNANDO D. CAMARGO. The Hippo signaling pathway and stem cell biology[J]. Trends in Cell Biology, 2012, 22(7):339-346.
[35]PAN D. The hippo signaling pathway in development and cancer[J]. Dev Cell. 2010,19(4):491-505
[36]DUOJIA PAN. The Hippo Signaling Pathway in Development and Cancer[J]. Developmental Cell. 2010, 10(19):401-505.
[37]HSIEH AL,WALTON ZE, ALTMAN BJ,et al.MYC and metabolismon the path to cancer[J].Semin Cell Dev Biol,2015,43:11-21.
[38]DANG CV.MYC the path to cancer[J].Cell,2012,149(1):22-35
[39]ZENG W,SUN H,MENG F,et al.Nuclear c-MYC expression level is associated with disease progression and potentially predictiv of two year overall survival in prostate cancer [J].Int J Clin Exp Pathol,2015,8(2):1878-1888.

附录

引物名称	引物序列(5’—3’)
METTL3- Forward Primer	CGCGCCTTATTCGAGT
METTL3- Reverse Primer	TAGATCCAAGTGCCCCGAGT
CCND1- Forward Primer	AGCTGTGCATCTACACCGAC
CCND1- Reverse Primer	GAAATCGTGCGGGGTCATTG
ID1- Forward Primer	AATCCGAAGTTGGAACCCCC
ID1- Reverse Primer	GGAACGCATGCCGCCT
LATS2 - Forward Primer	CGCCCCTGGAGAGAGTGA
LATS2 - Reverse Primer	TCTTTCCTTCCATTTTTGTAGTTCC
c-MYC - Forward Primer	TTCATAACGCGCTCTCCAAG
c-MYC - Reverse Primer	CAGAGCGTGGGATGTTAGTGT
SCRIB - Forward Primer	CGCCTGTCACCGGACTTT
SCRIB - Reverse Primer	AAGCACCAGAGCCACTTCTC
YAP1- Forward Primer	TGAGGAGCTTTTAGCATTGGTGCAGT
YAP1- Reverse Primer	AGCAGTGTGGTTACTTTTCCAGGTT
YWHAG- Forward Primer	AAGAATTGCAGCGAGACCCA
YWHAG- Reverse Primer	TTTGCTGATCTCGTGGGCTT

综述

RNA N6-甲基腺苷（m6A）修饰在生物体中作用以及其在肿瘤中的研究

在生物体内，遗传信息由 DNA 转录成为 RNA 在翻译成蛋白质行驶生物过程。而近年表观遗传学的兴起。在基因组上一系列可逆的修饰能调控基因到蛋白的过程。DNA[1-5]和组蛋白[6-9]的修饰基本上调节了基因表达，定义细胞状态和影响细胞分化和发育。虽然 DNA 和蛋白质均受可逆化学反应影响。但在 2010 芝加哥大学提出，这一过程，不仅仅由 DNA 及组蛋白上的修饰调控，RNA 也有类似的可逆性修饰参与基因的最终表达[10]。 RNA 在生物系统中有着关键的作用，不仅仅是通过遗传信息从 DNA 到蛋白质，而且参与调节各种生物过程。早已知道各种功能的 RNA 有着 100 多种化学修饰[11-14]，但这些 RNA 修饰具体起到什么样的功能，大多数仍是未知。

RNA N6-甲基腺苷（m6A）修饰是内源性丰度最高的 RNA 修饰方式，广泛存在于真核生物的 RNA 中。在 20 世纪 70 年代，m6A 被发现是最普遍的内部修改在多腺苷酸化的 mRNA 和长的非编码 RNA 中高等真核生物中的（lncRNA）[15]核糖体 RNA（rRNA）很小核 RNA（snRNA）和 tRNA 也含有 m6A。m6A 是广泛的服务于酵母，真核生物植物，苍蝇到哺乳动物，以及病毒 RNA 核[16-20]。包括 mRNA,lncRNA，microRNA 等。在近期的研究发现 m6A 为可逆 RNA 甲基化[21,22]。通过突变研究和底物获得甲基转移酶在体外的偏好确定的序列内容 m6A 是[G / A / U] [G> A] m6AC [U> A> C],具有高度的生物保守性[23-25]。

探测 RNA 中 m6A 的总量通过几种方法，包括二维薄层析[30]，斑点印迹[15]和高性能液相色谱与三重四极杆结合串联质谱（HPLC-QqQ-MS / MS）[21,22]。在 2012 年之后，两个独立的研究开发了一种 m6A RNA 免疫沉淀方法，然后通过高通量测序(MeRIP-seq)绘制出分辨率为 17,18 ~100 核苷酸的 m6A RNA 甲基化基因图谱[23]。简单地说就是把 mRNA 片段化，使用免疫沉淀进行免疫沉淀 m6A 靶向抗体，连接到测序衔接子，逆转录成 cDNA，用扩增 PCR 并进行高通量测序。得到的基因图谱显示 m6A 是广泛分布于 7,000 多个 mRNA 和 300 个人类细胞中的非编码 RNA（ncRNA）转录物，在 3'未翻译的密码子周围富集区域（3'UTR）和内部长外显子。许多 m6A 峰值都是在人类和老鼠之间保守。在不同的条件下能观察到某些峰的变化。并且从酵母到老鼠到人，在个物种之间检

测到的高度类似的 m6A 序列共有序列 ANRG m6ACNNU（其中 R 表示 A 或 G，和 N.代表任何核苷酸）表明这在生物体内有着高度的保守性。

基于抗体的分析 m6A 无法提供单核苷酸信息分辨率：虽然这种方法确定了含有 m6A 的 RNA 片段，但难以区分来自未修饰腺嘌呤的 m6A 通过测序阻碍精确定位这些片段中的 m6A 位点。多个 RRACH（其中 H 表示 U，A 或 C）图案可以彼此相邻地存在，并且修饰也可以在非共识位点处发生。此外，基于抗体的分析不能揭示该分数在每个特定位点修饰的细胞 RNA。一个最新方法被称为特异性位点切割和放射性标记结扎辅助提取和薄层色谱（SCARLET），用于确定 m6A 在腺苷酸的百分比具有单核苷酸分辨率的特定位点[33]。该方法使用 RNase H 引导通过序列特异性 2'-OMe / 2'-H 嵌合寡核苷酸切割候选位点的 5'末端随后的标记和检测。应用这个方法揭示了两种 lncRNA 和三种 mRNA 的真正的 m6A 位点和量化甲基化组分（11-77％）。这些结果连同 20％之前报道过的 m6A 修饰牛催乳素 mRNA[34]，表明许多 m6A 位点在 mRNA 和 lncRNA 中不完全甲基化。在事实上，大多数 m6A 共有序列位点都是在哺乳动物 mRNA 中没有甲基化[26,27]。在研究 m6A 中鉴定了调控这种动态 RNA 甲基化的蛋白质，习惯分别成甲基转移酶，去甲基化酶和识别特定甲基化的蛋白为“writer”，“eraser”和“reader”，以及 m6A 在几种基因表达转录后调控机制中起到的作用。

M5A 修饰相关蛋白

METTL3 是哺乳动物细胞中 m6A 甲基化转移酶复合物。m6A mRNA 甲基化由多组分甲基催化转移酶复合物，从 HeLa 细胞的核中分离出来[25,38]。METTL3（MT-A70）是一种 70 kDa 的蛋白质[28]。 METTL3 从酵母到人的真核生物中具有高度保守性。在 HeLa 细胞中敲低 METTL3 的表达，总 m6A 水平下降了约 30％。在 HepG2 细胞中同样敲低 METTL3，发现诱导了细胞凋亡，可能通过细胞凋亡激活 p53 介导的途径[26,31]。在另一实验发现带有重组标记的人 METTL3 蛋白自身的活性较低，在体外获得最佳活性需要其他成分。

系统发育分析 METTL3 家族的甲基转移酶在人体内基因组鉴定 METTL14 和 METTL3 接近，具有保守基序的 METTL3 的同源物含有 Asp-Pro-Pro-Trp 或 Glu-Pro-Pro-Leu41。我们发现在 HeLa 和 293FT 细胞中敲低 METTL14，不敲低 METTL3，也会导致 RNA m6A 水平降低[32]。生化表征透露这两种蛋白质形成稳定的复合物化学计量比为 1:1。在体外甲基 METTL14 的活性略高于 METTL3，甲基转移酶复合物导致甲基化显着增强活动。这种异二聚体也表现出强烈的同

源性。m6A 共有序列和 a 对体外结构较少的 RNA 的适度偏好。METTL3 和 METTL14 共定位于核斑点，并且异二聚体形成甲基转移酶复合物。METTL14 可能参与蛋白质核斑点中的蛋白质相互作用。绑定基质 RNA 中 METTL3 和 METTL14 的位点，甲基转移酶复合物的沉默导致其靶 RNA 的丰度和半衰期增加。小鼠胚胎干细胞（mESCs）的相关研究也表明 METTL3 和 METTL14 作为复合物起作用[33]。

METTL14 包括富含甘氨酸的序列和氨基中的潜在卷曲螺旋末端，可能参与蛋白质核斑点中的蛋白质相互作用。绑定基质 RNA 中 METTL3 和 METTL14 的位点，可用光活化的核糖核苷增强的交联和免疫沉淀（PAR-CLIP）测定，得到结果含有与已知的相似的 m6A 共有序列。有趣的是，沉默甲基转移酶复合物导致丰度增加和靶 RNA 的半衰期一致随着 m6A 作为负面监管的新兴角色基因表达的基因。一项相关的研究小鼠胚胎干细胞（mESCs）也表明 METTL3 和 METTL14 作为复合物起作用[33]。发现第二种活性甲基转移酶核心综合体中的组件后为什么 m6A 甲基转移酶复合物由两个活性成分组成，两者都结合在一起甲基供体 S-腺苷-1-甲硫氨酸（SAM）。

杂复合物可以允许选择性调整通过翻译后的甲基化活性修改每个组件以影响差异不同的底物转录物，因此具有影响在不同的生物途径上。两个异二聚体最佳需要甲基转移酶组分几种其他已知 RNA 甲基转移酶的活性[33-36]。通常，一个子单元具有 SAM 结合口袋，和另一个非催化亚基要么稳定催化亚基，要么增强催化亚基通过形成连续的基质结合的活性面对。但是，METTL3 和 METTL14 都是活跃的。这种复合物的晶体结构将有所帮助揭示两种酶之间的协同作用与每个活动组件关联的属性。于是发现 WTAP 是 m6A 甲基转移酶复合体在体内的第三个关键组分。酵母双杂交筛选有鉴定出 37kDa 的 FKBP12 相互作用蛋白。酵母中的 Mum2 作为 METTL3 的伴侣蛋白这些生物体中的同源物[35]。这两种蛋白质在人类中是 Wilms'肿瘤 1 关联的同源物(WTAP)。WTAP 最初被确定作为与 Wilms 肿瘤结合的剪接因子 1 蛋白（WT1）[36]，它对细胞周期进程和早期哺乳动物胚胎发育至关重要。我们发现 WTAP 的敲低会导致 WTAP 降低 HeLa 和 293FT 细胞中的总 m6A 水平[32]。 WTAP 与 METTL3 和 METTL14 相互作用，并与核斑中的 METTL3-METTL14 异二聚体共定位，参与 m6A RNA 甲基化。事实上，WTAP 的敲低导致最大的这些细胞系中 m6A 水平降低，这表明 WTAP 在细胞中调控 m6A 水平具有重要作用。并与 METTL3-METTL14 异二聚体进行校

准核斑点参与 m6A RNA 甲基化。事实上，WTAP 的击倒导致最大的这些细胞系中 m6A 水平降低，这表明 WTAP 在决定细胞 m6A 中具有重要作用。 PAR-CLIP 测定显示 WTAP 共享 GACU 的类似结合序列;也就是说，序列由 WTAP 约束与 GGAC 适度重叠由 METTL3 和 METTL14 结合的序列。正如 PAR-CLIP 所确定的那样，这些目标具有与含有 m6A 的转录物重叠约 50％，其中进一步表明 METTL3，METTL14 和 WTAP 形成 m6A 的甲基转移酶复合体的核心。这些的大部分结合位点在内含子中发现了三种蛋白质（29-34％），这意味着甲基化是在共转录的拼接之前或同时拼接。作为 WTAP 最近的一项研究被认为是一个影响因素，表示 WTAP 或 METTL3 的敲低产生不同的含有 m6A 的转录物的同种型，这表明甲基化可能影响剪接。WTAP 如何增强甲基化活性 METTL3 和 METTL14 在体内的作用。可能是 WTAP 可能有助于招募 METTL3 和 METTL14 他们的靶 mRNA。 WTAP 也被证明是对 METTL3 核斑点定位至关重要，和 METTL14 一起可能会影响甲基化这些蛋白质的效率。已知有许多研究表明 WTAP 与许多蛋白质和 lncRNA 相互作用[44]，可以也招募其他蛋白质或酶到甲基转移酶复合物，这些额外因素可能会影响通过直接的甲基化活性和选择性相互作用或翻译后修饰。未来研究以确定与之相互作用的其他因素或修改两种甲基转移酶对于站立 m6A 沉积的选择性。我们可能会能够回答诸如细胞如何选择等问题甲基化某些 RNA 位点，以及 m6A 如何适用于 3'UTR 和长外显子的潜在的相互作用 RNA 甲基化，转录调控之间还可以进一步研究和拼接。酵母中的 Mum2-Ime4-Slz1（MIS）复合物介导减数分裂期间的 mRNA 甲基化。METTL3 在酵母中的对数并且对诱导至关重要酵母孢子形成甲基化的另外两个组成部分 Mum2 和特异性亮氨酸拉链基序蛋白 1（Slz1）已被识别通过酵母双杂交实验。Mum2 与人 WTAP 同源，而 Slz1 缺乏哺乳动物同源物。有趣的是，增加了减数分裂期间的 m6A 水平主要由 Ime1 引发（减数分裂的主要调节因子），转录诱导 SLZ1。 Ime4 和 Mum2 在之前表达诱导减数分裂，然后 Slz1 从中招募它们细胞质到细胞核[32]。这种核仁局部化 MIS 复合体对于积累 MIS 非常重要满级 m6A。与酵母，哺乳动物细胞相反缺乏 Slz1，哺乳动物和植物的同源物甲基转移酶复合物主要位于核中斑点而不是斑点

哺乳动物中的 m6A“eraser”FTO 对 RNA 中 m6A 的去甲基化。 在 2011 年，α-酮戊二酸依赖性双加氧酶 FTO 的发现因为第一个 RNA 去甲基化酶是一个重要的突破[21]。2007 年，三项独立研究显示第一个内含子的单核苷酸多态性 FTO

与体重指数和多个人群中肥胖的风险强烈关联[37-39]。在成年小鼠中，Fto在大脑中具有最高的表达水平，特别是在下丘脑内。删除或过度表达小鼠模型中的Fto会导致小鼠体重或食物摄入量相关改变[40,41]。Fto也会影响小鼠发育：Fto敲除小鼠显示后期增加产后致死率和生长迟缓[40]，但是仍未知FTO有效地使RNA和DNA中的m6A去甲基化。后续实验表明，在HeLa和293FT细胞中沉默FTO，增加了总RNA m6A水平。过度表达FTO降低RNA上的m6A水平[21]。基于细胞实验的结果，连同大多数哺乳动物细胞的观察结果和组织含有非常低的水平（百万分之几）m6A对基因组DNA的影响，使我们得出结论m6A关于核RNA（包括mRNA，lncRNA和其他类型的RNA）是FTO的主要底物。最近研究表明，m6A对三种mRNA种类有影响可以通过FTO在体内去甲基化，并且这种功能似乎影响神经元活动[42]。

ALKBH5对RNA中m6A的去甲基化。ALKBH5是AlkB家族的另一种蛋白质在mRNA中对m6A的去甲基化活性和其他类型的核RNA[22,43]。在人细胞系敲低ALKBH5不仅导致RNA总m6A水平增加还加速这些的RNA从细胞核到细胞质[22]，但是具体机制尚不清楚。Alkbh5有小鼠睾丸中的最高表达水平。Alkbh5敲除雄性小鼠表现出异常的精子起源，这可能是表达改变的结果精子发生相关基因[22]。ALKBH5及其去甲基化活性影响新生儿mRNA合成和剪接率[22]。与FTO不同，已确定ALKBH5的直接免疫沉淀已显示结合的RNA底物和ALKBH5成为mRNA结合的蛋白质组的一部分，与mRNA和其他RNA紧密的相互作用[44]。

对于m6A组来说有一个生物学功能，需要通过特定蛋白质“reader”来识别通过。这个过程可能类似于读取5-甲基胞嘧啶的蛋白质的作用（m5C;DNA中也称为5mC），或甲基化或乙酰化为了表现出组蛋白的氨基酸残基与修饰相关的生物学功能并实现可逆调整。YTHDF2优先识别含m6A的mRNA和调节mRNA稳定性。使用pulldown实验，我们已经确定YTH结构域的三种细胞质蛋白，YTHDF1-3，作为选择性m6A结合蛋白哺乳动物细胞提取物[26,45]。YTH域家族由丰富的RNA结合蛋白组成以前没有分配明确的功能。先前的研究确定哺乳动物YTHDF蛋白结合在G [G> A] m6ACU上含有m6A的RNA;然而，YTHDF2与mRNA结合较短的poly（A）尾巴似乎并没有影响[45]。几种细胞质mRNA衰变途径是[46,47]。YTHDF2介导的mRNA降解影响成千上万个mRNA分子的是一种独特的过程，通过mRNA m6A甲基化和去甲基化调整。

相对于未甲基化 RNA 的共有序列相同的序列。另外，RNA 探针即可保守的 GAC [U> A]基序;因此，占用 YTHDF2 类似于 m6A 的分布模式关于 mRNA。值得注意的是，YTHDF2 的敲低导致了这些 RNA 靶标的半衰期缩短，但也有正面影响，在积极翻译中对 mRNA 水平的影响。核糖体分析进一步表明 YTHDF2 改变其 mRNA 靶标的核糖体占据。这些结果表明 YTHDF2 在 RNA 衰变中起作用。YTHDF2 和荧光素的荧光免疫染色其同源 mRNA 的原位杂交显示 YTHDF2 通过 C 末端与 m6A 结合 YTH 结构域并将同源 mRNA 定位于加工体（P 体）加速降解通过其 N 末端 Pro / Gln / Asn 富集结构域。确切的 RNA 降解机制需要进一步阐明;然而，YTHDF2 与 mRNA 结合较短的 poly（A）尾巴似乎并没有影响去腺苷酸化过程[46]。几种细胞质 mRNA 衰变途径是一致的。YTHDF2 介导的 mRNA 降解影响成千上万个 mRNA 分子的是一种独特的过程，取决于甲基化目标 mRNA 的结果可以是可逆的通过 m6A 甲基化和去甲基化调整。

这一发现与负相关一起 m6A 的 mRNA 稳定性一般表现为甲基转移酶的敲除[40]，主要表明 m6A 对 RNA 的作用：调节的降解甲基化 RNA。这个过程是通过选择性 m6A 识别和随后的重新定位 Reader 或效应蛋白质。控制权非翻译 mRNA（或其他）的稳定性 RNA 种类）通过依赖 YTHDF2 的机制在各种情况下，可能是一种重要的用于选择性消除一组 RNA。有趣的是，Mmi1 和 YTHDF 亲属的同源物种的细胞，是用于消除减数分裂特异性转录本的方法在减数分裂期间。但是，m6A 的存在却没有报道，缺乏同源物 METTL3 和 METTL14。 m6A 的潜在存在在 mRNA 中及其在粟酒裂殖酵母中的功能作用应该是进一步调查。

hnRNP 可能是潜在的核 m6AReader。除了 YTH 结构域蛋白质和其他细胞质 mRNA 结合蛋白，pulldown 实验还鉴定了异质蛋白质核核糖核蛋白（hnRNP）类型为潜在的 m6A 选择性结合蛋白[17]。已知形成核可能影响 mRNA 局部化的核蛋白颗粒 hnRNPs 也可以阻止 hnRNPs 的结合剪接因子并影响可变剪接。额外需要进行实验来研究连接在 hnRNPs 和 m6A 之间。m6A 和 m6A 衍生修饰的反读物。甲基的存在也可能不利 RNA 结合蛋白与修饰的蛋白结合 RNA。这种反阅读机制还没有观察到 m6A。 m6A 修改很广泛分布在 mRNA 转录本的 3'UTR 中的腺苷酸由许多 RNA 结合蛋白结合的区域调节 mRNA 代谢和翻译。它是可能的某些反阅读机制存在于晚期甲基化 mRNA 的命运。 m6A 也是众所周知的通过细胞先天免疫保护 RNA 免受识别蛋白质。 Toll 样受体 3（TLR3）和 TLR7 识别未修饰的双链和单链 RNA 作为侵入性 RNA 种类并选择性地靶向它们为降解。

纳入 m6A 等转染的外源 RNA 中的 RNA 修饰可以减少先天免疫系统的识别防止不必要的退化，这会增加他们的表达。可能是一种反阅读机制在这个过程中运作。

某些 RNA 修饰，如已知假尿苷（Ψ）会导致二次和二次三级结构变化。m6A 修改减少 A：U 的基准配对能量，但这种差异可能会改变平衡 RNA 的某些二级和三级结构。该改变的结构可能对结合产生影响特定蛋白质，导致间接阅读和规。在最近的一项研究中 HuR(ELAVL1)一种众所周知的 RNA 结合蛋白影响乳腺癌中许多 mRNA 转录本的稳定性。m6A 修饰影响了 HuR 在体外与不同 RNA 探针结合的能力[42]。在这种特殊情况下，RNA 结构被改变了甲基化可能间接地促成了同源 HuR 结合位点的可能性。然而，HuR 在体内识别的共有序列是不同于含有 m6A 的序列[51,52]。该细胞连接的程度和细节 HuR 和 m6A 仍需进一步研究。

到目前为止，还没有人知道这种间接的细胞实例阅读机制和 m6A 的生物学结果。最近在发现和特征方面的突破 m6A writer，eraser 和 reader 的联系在一起与高通量分析的并行开发在转录组范围内描述这种甲基化规模，设定阶段并为功能性投资提供工具，旨在确定机制的引力 m6A 被翻译成生物学结果。过去的研究使用广谱甲基化抑制剂的产生了不确定的结果。但现在 m6A writer，eraser 和 reader 蛋白已被明确定义，这些机制的紊乱可以导致更具体表型结果和实验观察这将有助于阐明 m6A 和 m6A 的生物学作用潜在的机制。一个工具方面这项努力将是对表型水平进行分类受到 m6A 影响的人群。首先是效果水平整个生物体或组织，这些水平的研究可以揭示 m6A 的组织特异性及其相关性某些疾病和生物过程（发育，不孕，致癌，干燥，减数分裂和昼夜节律）。第二是效果途径水平（例如，p53 介导的途径， Notch 信号传导，哺乳动物的营养传感雷帕霉素复合物 1（mTORC1）和 SIS）。第三是机制层面的角色（例如，剪接体和核输出机制）。Reader 蛋白质和他们相关的识别机制至关重要揭示和理解这些角色。通过甲基化进行转录后调控目标转录物的依赖性定位。对我们来说知识，YTHDF2 的深入表征作为第一个 m6AReader 描绘了第一个成立 m6A 介导的分子通路：结合 YTHDF2 成千上万的 mRNA 转录本（也是某些 ncRNA 转录本）导致定位从可翻译池中结合的 mRNA 的衰变，从而影响翻译状态和 mRNA 的半衰期。这一发现有两个根本优点。首先，它表明了 m6A 的主要功能甲基化作为可逆标记是影响 mRNA 稳定性，在沉默甲基转移酶时观察到非常吻合 m6A 水平与

转录本丰度之间的与负相关性。事实上，这种甲基化通常与 mRNA 结合，具有较短的半衰期。其次，这个例子说明了如何选择通过结合蛋白可以读取 m6A 标记，影响靶 RNA 的定位，从而提供适用于其他潜在蛋白模型可能广泛影响 RNA 的运输，储存，稳定性，翻译和拼接。虽然转录调控具有重要意义。尽管不是明确 m6A 是否在调节中具有直接的顺式作用特异性地输出含有 m6A 的 RNA 分子或是否是扰动的间接后果 RNA 出口机制。m6A RNA 修饰参与引发硝酸盐过程中酵母细胞到双潜能状态和减数分裂饥饿。通过仔细监测甲基，最近的一项研究表明，不同阶段的情况有关甲基化对于动力学控制很重要减数分裂前期的 RNAs。虽然没有观察到半衰期的显着变化含有 m6A 的 RNA，这些 RNA 的可及性翻译可以通过互动调制具有潜在 Reader 蛋白质。类似于提出 m6A 在加速两种 RNA 中的作用哺乳动物细胞的输出和降解，m6A 可能确保 RNA 转录本的更快转换在减数分裂前期很重要。

同样，在鼠、胚胎干细胞 mESC 的另一项研究显示，m6A 甲基化加速转录物衰变，影响干细胞维持和分化。有趣的是，与繁殖相关的基因，发育调节因子在 METTL3 的靶基因中显着富集 mESC 中的 METTL14 和 METTL14。特别是，m6A 可以使用发育调节剂转录本表明甲基化对维持很重要和 mESCs 的分化。已知 mRNA 的调节在其中起关键作用胚胎发育。受精后，产妇 mRNA 需要以程序化方式降解。mRNA 上的甲基化可能会影响这一过程通过改变焦油的定位和半衰期得到 mRNA 转录本。这种甲基化可以标志着特定的 RNA 种类，因此分化动力学中的母体和合子 mRNA 之间的关系 ER。也许可以通过遗传信息通过协调的 RNA 进入几代细胞甲基化和去甲基化活性。

基于 m6A 的规则的优点和特异性。第一个被表征的 m6A 读取蛋白是已知影响超过 3,000 种不同的 mRNA 转运。我们提出可逆 RNA 甲基。一般而言，通路已演变为影响过程这涉及到大群体表达的变化基因。这个属性与潜力密切相关在 RNA 上可逆甲基化水平的优点。除了增加复杂性监管网络，这种机制可能允许迅速表达时对信号和刺激的反应一组蛋白质的含量（可以是几十个成千上万）需要快速调整。当 DNA 水平的反应（即转录）可能太慢了;当回应时蛋白质水平可能需要特定的相互作用或对数十至数千种蛋白质的修饰，其中很难实现。可逆甲基化或其他 mRNA 的修饰形式提供了最好的选项。可以经历逆转的特定序列修改，因此受到监管，可以容易地包括在一组 mRNA 转录物中（例如，在它们的 3'UTR 处）和 lncRNA 依次排列影响 RNA 的稳定性，定位和可译性，如

YTHDF2 的例子所示。

可逆性的 RNA 甲基化与 DNA 的表观遗传有许多共同特征。表达水平和“writer”，“eraser”可以调节 RNA 甲基化，和“reader”最终影响蛋白质表达。而表观遗传 DNA 和组蛋白修饰主要影响转录事件，可逆性 RNA 甲基化主要对其有影响转录后基因表达和调控可直接影响蛋白质的产生。m6A 甲基化广泛分布在生物体内的所有 RNA 上。已知其在生物体内影响 RNA 的稳定及蛋白的翻译。但在癌症中的研究还是有限的，是一个值得探究方向。

M6A 修饰相关肿瘤研究

而在人类肿瘤之中，已经有报道 m6A 修饰与肿瘤的增殖、分化、成瘤、增殖、侵袭和转移相关[52,53]。在急性髓细胞白血病(AML)中 FTO 的高度表达会导致 t(11q23)/MLL 重排, t(15;17)/PML-RARA, FLT3-ITD 及 NPM1 基因的突变，促进白血病的发生发展 [54].METTL3/14 在造血干细胞/祖细胞(HSPCs)和含 t(11q23)、t(15;17)或 t(8;21)的 AML 细胞中表达，使得 HSPCs 的终末髓样分化，促进 AML 细胞的增殖[55]。WTAP 则在细胞增殖和阻止白血病的分化中起作用[56]。

在 AML 中 c-MYC、BCL2 和 PTEN 等基因的 m6A 修饰促进了他们的翻译[56]。METTL14 通过 m6A 修饰调控其靶基因 MYB/MYC，导致了白血病的产生[55]。YTHDF2 还使 Tal1 的 mRNA 稳定并在 AML 中的扩增[57]。这些研究证实 m6A 修饰在 AML 中起到的重要作用。

据报道 METTL3/14 抑制了胶质瘤干细胞的生长，自我更新及肿瘤的发生,而 FTO 和 ALKBH5 能够通过 调节 ADAM19 和 转录因子 FOXM1 使得胶质母细胞瘤患者有着更差的生存率[58,59]。

在肺癌中 m6A 去甲基化酶 FTO 被认为是肺鳞癌(LUSC)的预后因子，他能够促进细胞增殖和侵袭，通过调控 MZF1 表达抑制细胞凋亡[60]。METTL3 作为肺癌的致癌基因，通过增加 EGFR 和 TAZ 的表达，促进细胞生长、生存和侵袭[52]。METTL3-eIF3 引起的 mRNA 环化促进肺腺癌的转译和癌变[52]。METTL3 对于促进非小细胞肺癌(non-small cell lung carcinoma, NSCLC)中赖氨酸残基 K177、K211、K212 和 K215 的肿瘤生长具有重要意义[61]。这些研究展示了 m6A 甲基化与去甲基化酶 METTL3 和 FTO 在肺癌中的重要作用。

肝细胞癌(HCC)中发现 METTL3 与 HCC 患者的不良预后相关 ，促进 HCC 增殖, 迁移侵袭能力。利用 m6A 识别蛋白 YTHDF2 导致 SOCS2 转录后沉

默[62]。但是，METTL14 却是抗转移因子，通过调控 m6a 调节 miRNA 的成熟。这些研究展示了肝癌发生的表观遗传改变。同时也让我们发现 METTL3/14 在同一肿瘤中可能起到不同的预示。

在乳腺癌中 METTL3 显示与肿瘤的侵袭性相关。METTL3 诱导哺乳动物乙型肝炎相互作用蛋白(HBXIP)的表达通过抑制肿瘤抑制因子 let-7 g 促进乳腺癌增殖[63]。还有研究显示，ALKBH5 降低了 NANOG mRNA 的 m6A 的水平，使其稳定性增强，导致乳腺癌干细胞(BCSCs)中 NANOG mRNA 和蛋白水平升高[64]。表观遗传学在肿瘤中起到的重要功能逐渐被挖掘。

综述参考文献

[1]SUZUKI MM, BIRD A. DNA methylation landscapes: provocative insights from epigenomics[J]. Nature Rev Genet,2008,9:465–476.

[2]KOHLI RM, ZHANG Y. TET enzymes, TDG and the dynamics of DNA demethylation[J]. Nature,2013,502:472–479.

[3]JONES PA. Functions of DNA methylation: islands, start sites, gene bodies and beyond[J]. Nature Rev Genet,2012,13:484–492.

[4]BRANCO MR, FICZ G, REIK W.Uncovering the role of 5-hydroxy-methylcytosine in the epigenome[J]. Nature Rev Genet,2012,13:7–13.

[5]Bhutani N, Burns D.M, Blau HM. DNA demethylation dynamics[J].Cell.2011;146,866–872.

[6]STRAHL BD, ALLIS CD. The language of covalent histone modifications[J]. Nature.2000,403:41–45.

[7]SHI Y. Histone lysine demethylases: emerging roles in development, physiology and disease[J].Nature Rev,2007,8：829–833.

[8]KLOSE RJ, KALLIN EM, ZHANG Y. JmjC-domain-containing proteins and histone demethylation[J].Nature Rev Genet,2006,7:715–727.

[9]BIRD, A. MOLECULAR BIOLOGY: Methylation Talk Between Histones and DNA[J]. Science, 2001, 294(5549):2113-2115.

[10]HE C. Grand challenge commentary: RNA epigenetics[J]. Nature Chem Biol, 2010, 6:863–865.

[11]Grosjean H , Benne R .Modification and editing of RNA[J]. 1998.

[12]Grosjean, Henri. FINE-TUNING OF RNA FUNCTIONS BY MODIFICATION AND EDITING[M]. Springer Berlin Heidelberg, 2005.

[13]MACHNICKA MA et al. MODOMICS: a database of RNA modification pathways—2013 update[J]. Nucleic Acids Res,2013,41:262–267.

[14]MOTORIN Y, HELM M. RNA nucleotide methylation[J]. Wiley Interdiscip Rev RNA,2011,2:611–631.

[15]WEI CM, GERSHOWITZ A, MOSS B.Methylated nucleotides block 5′ terminus of HeLa cell messenger-RNA[J].Cell,1975,4:379–386.

[16]KRUG RM, MORGAN MA ,SHATKIN AJ. Influenza viral mRNA contains internal N6-methyladenosine and 5′-terminal 7-methylguanosine in cap structures[J]. Virol,1976,20:45-53.

[17]ROTTMAN FM, DESROSIERS RC, FRIDERICI K.Nucleotide methylation patterns in eukaryotic mRNA[J]. Prog Nucleic Acid Res & Mol Biol,1976,19:21-38 .

[18]BEEMON,K. & KEITH.Localization of N6-methyladenosine in the Rous sarcoma virus genome[J].Mol Biol.1977,113:165–179.

[19]SCHIBLER, U., KELLEY, D. E. & PERRY, R. P. Comparison of methylated sequences in messenger RNA and heterogeneous nuclear RNA from mouse L cells[J].Mol Biol. 1977,115:695-714.

[20]WEI, C. M. & MOSS, B. Nucleotide sequences at the N6-methyladenosine sites of HeLa cell messenger ribonucleic acid[J].Biochemistry,1977,16:1672-1676.

[21]JIA G. et al. N6-methyladenosine in nuclear RNA is a major substrate of the obesity-associated FTO[J]. Nature Chem Biol,2011,7:885-887 .

[21]ZHENG, G. et al. ALKBH5 is a mammalian RNA demethylase that impacts RNA metabolism and mouse fertility[J]. Mol Cell,2013,49:18-29.

[22]NARAYAN, P. & ROTTMAN, F. M. An in vitro system for accurate methylation of internal adenosine residues in messenger RNA[J]. Science,1988,242:1159-1162.

[23]CSEPANY T.,LIN A.BALDICK C. J. JR & BEEMON, K. Sequence specificity of mRNA N6-adenosine methyltransferase.[J]. Biol Chem. 1990,265:20117–20122 .

[24]NARAYAN, P., LUDWICZAK, R. L., GOODWIN, E. C. & ROTTMAN, F. M. Context effects on N6-adenosine methylation sites in prolactin mRNA[J]. Nucleic Acids Res, 1994,22:419-426.

[25]DOMINISSINI, D. et al. Topology of the human and mouse m6A RNA methylomes revealed by m6A-seq[J]. Nature,2012,485:201-206 .
[26]MEYER, K. D. et al. Comprehensive analysis of mRNA methylation reveals enrichment in 3′ UTRs and near stop codons[J]. Cell,2012,149:1635-1646 .
[27]BOKAR, J. A., SHAMBAUGH, M. E., POLAYES, D., MATERA, A. G. & ROTTMAN, F. M. Purification and cDNA cloning of the AdoMet-binding subunit of the human mRNA (N6-adenosine)-methyltransferase[J]. RNA,1997,3:1233–1247.
[28]CARROLL, S. M., NARAYAN, P. & ROTTMAN, F. M. N6-methyladenosine residues in an intron-specific region of prolactin pre-mRNA[J]. Mol Cell Biol,1990,10: 4456–4465.
[29]KIERZEK, E. & KIERZEK, R. The thermodynamic stability of RNA duplexes and hairpins containing N6-alkyladenosines and 2-methylthio-N6-alkyladenosines[J]. Nucleic Acids Res,2003,31:4472-4480.
[30]LIU J.et al. A METTL3–METTL14 complex mediates mammalian nuclear RNA N6-adenosine methylation[J]. Nature Chem Biol,2014,10:93-95.
[31]WANG, Y. et al. N6-methyladenosine modification destabilizes developmental regulators in embryonic stem cells[J]. Nature Cell Biol, 2014,16:191-198 .
[32]ZHONG, S. et al. MTA is an Arabidopsis messenger RNA adenosine methylase and interacts with a homolog of a sex-specific splicing factor[J]. Plant Cell,2008,20: 1278–1288.
[33]AGARWALA, S. D., BLITZBLAU, H. G., HOCHWAGEN, A. & FINK, G. R. RNA methylation by the MIS complex regulates a cell fate decision in yeast[J]. PLoS, 2012,8:e1002732.
[34]Little, N. A., Hastie, N. D. & Davies, R. C. Identification of WTAP, a novel Wilms' tumour 1-associating protein. Hum. Mol.2000;9, 2231–2239.
[35]DINA, C. et al. Variation in FTO contributes to childhood obesity and severe adult obesity[J]. Nature,2007,39:724–726 .
[36]FRAYLING, T. M. et al. A common variant in the FTO gene is associated with body mass index and predisposes to childhood and adult obesity[J]. Science, 2007, 316:889–894.
[37]SCUTERI, A. et al. Genome-wide association scan shows genetic variants in the

FTO gene are associated with obesity-related traits[J]. PLoS, 2007, 3:e115.
[38]FISCHER J. et al.Inactivation of the Fto gene protects from obesity[J].Nature,2009,458:894–898.
[39]CHURCH C. et al. Overexpression of Fto leads to increased food intake and results in obesity[J]. Nature Genet,2010,42:1086–1092 .
[40]HESS, M. E. et al.The fat mass and obesity associated gene (Fto) regulates activity of the dopaminergic midbrain circuitry[J]. Nature Neurosci, 2013,16: 1042-1048.
[41]ZHENG, G. et al. Sprouts of RNA epigenetics: the discovery of mammalian RNA demethylases[J].RNA Biol,2013,10:915-918.
[42]BALTZ, A. G. et al. The mRNA-bound proteome and its global occupancy profile on protein-coding transcripts[J]. Mol Cell,2012,46:674–690.
[43]WANG, X. et al. N6-methyladenosine-dependent regulation of messenger RNA stability[J]. Nature,2014,505:117–120.
[44]SCHOENBERG, D. R. & MAQUAT, L. E. Regulation of cytoplasmic mRNA decay[J]. Nature Rev,2012,13:246–259 .
[45]ISKEN, O. & MAQUAT, L. E. The multiple lives of NMD factors: balancing roles in gene and genome regulation[J]. Nature Rev,2008,9:699–712 .
[46]KHAN, Z. et al. Primate transcript and protein expression levels evolve under compensatory selection pressures[J]. Science,2013,342:1100–1104.
[47]WU, L. et al. Variation and genetic control of protein abundance in humans[J]. Nature,2013,499:79–82.
[48]SALETORE, Y. et al. The birth of the epitranscriptome: deciphering the function of RNA modifications[J]. Genome Biol,2012,13:175 .
[49]KARIJOLICH, J. & YU, Y. T. Converting nonsense codons into sense codons by targeted pseudouridylation[J]. Nature,2011,474:395–398.
[50]FERNANDEZ, I. S. et al. Unusual base pairing during the decoding of a stop codon by the ribosome[J]. Nature,2013,500:107–110.
[51]GE, J. & YU, Y. T. RNA pseudouridylation: new insights into an old modification. Trends Biochem[J]. Sci,2013,38: 210–218.
[52]LIN S, CHOE J, DU P, TRIBOULET R, GREGORY RI. The m6A

methyltransferase METTL3 promotes translation in human Cancer cells[J]. Mol Cell, 2016, 62(3):335–45.

[53]LIU J, ECKERT MA, HARADA BT, LIU S-M, LU Z, YU K, et al. m6A mRNA methylation regulates AKT activity to promote the proliferation and tumorigenicity of endometrial cancer[J]. Nat Cell Biol,2018,20(9):1074–83.

[54]LI Z, WENG H, SU R, WENG X, ZUO Z, LI C, et al. FTO plays an oncogenic role in acute myeloid leukemia as a N6-Methyladenosine RNA demethylase[J]. Cancer Cell. 2017;31(1):127–41

[55] WENG H, HUANG H, WU H, QIN X, ZHAO BS, DONG L, et al. METTL14 inhibits hematopoietic stem/progenitor differentiation and promotes Leukemogenesis via mRNA m6A modification[J]. Cell Stem Cell,2017,22(2):191–205 e9.

[56] BANSAL H, YIHUA Q, IYER S, GANAPATHY S, PROIA D, PENALVA L, et al. WTAP is a novel oncogenic protein in acute myeloid leukemia[J]. Leukemia. 2014,28(5):1171–4

[57]LI Z, QIAN P, SHAO W, SHI H, HE XC, GOGOL M, et al. Suppression of m6A reader Ythdf2 promotes hematopoietic stem cell expansion[J].Cell Res, 2018,28(9):904–17

[58] ZHANG S, ZHAO BS, ZHOU A, LIN K, ZHENG S, LU Z, et al. M(6)a demethylase ALKBH5 maintains Tumorigenicity of glioblastoma stem-like cells by sustaining FOXM1 expression and cell proliferation program[J]. Cancer Cell, 2017,31(4):591–606.e6.

[59] CUI Q, SHI H, YE P, LI L, QU Q, SUN G, et al. M(6)a RNA methylation regulates the self-renewal and tumorigenesis of glioblastoma stem cells[J]. Cell Rep. 2017,18(11):2622–34.

[60] LIU J, REN D, DU Z, WANG H, ZHANG H, JIN Y. m6A demethylase FTO facilitates tumor progression in lung squamous cell carcinoma by regulating MZF1 expression[J]. Biochem Biophys Res Commun,2018,502(4):456–64.

[61] DU Y, HOU G, ZHANG H, DOU J, HE J, GUO Y, et al. SUMOylation of the m6ARNA methyltransferase METTL3 modulates its function[J]. Nucleic Acids Res, 2018;46(10):5195–208.

[62] CHEN M, WEI L, LAW CT, TSANG FH-C, SHEN J, CHENG CL-H, et al.

RNA N6- methyladenosine methyltransferase-like 3 promotes liver cancer progression through YTHDF2-dependent posttranscriptional silencing of SOCS2[J]. Hepatol Baltim Md. 2017;67(6):2254–70

[63]CAI X, WANG X, CAO C, GAO Y, ZHANG S, YANG Z, et al. HBXIP-elevated methyltransferase METTL3 promotes the progression of breast cancer via inhibiting tumor suppressor let-7g[J]. Cancer Lett, 2018,415:11–9.

[64] ZHANG C, SAMANTA D, LU H, BULLEN JW, ZHANG H, CHEN I, et al. Hypoxia induces the breast cancer stem cell phenotype by HIF-dependent and ALKBH5-mediated m6A-demethylation of NANOG mRNA[J]. Proc Natl Acad Sci. 2016,113(14):E2047–56.

致谢

光阴似箭，岁月如梭，转眼之间到了毕业季。回顾年研究生生涯，过程漫长却又转瞬即逝。有失败懊恼之时，也有成功喜悦的时刻。最终印在脑子里的是各位尊敬的师长训练的科学思维，印在心里的是良师益友们教会的做人品质。

首先衷心感谢我的导师张传祥教授给予我的机会，让我在天津是泌尿外科研究所学习成长，是他引领我走进科研的世界。

衷心感谢牛远杰教授的辛勤培养和悉心教导！牛老师有渊博的学识，严谨的科研态度，高超的医术，高尚的医德，用他对科研的真诚与热情指导我们前进，更用他严谨的治学要求，心系病患的崇高医德以身作则指引我们做人。在2019冠状病毒疫情前，牛老师亲自率队支援湖北，面对危险，挺身而出，这大无畏的精神，鞭策着我们继续向牛老师学习。

衷心感谢尚芝群教授手把手的指导与培养。在实验室开展课题时，是尚老师手把手的教导使我们茁壮成长。尚老师渊博学识、严谨治学、求实作风让我们学习了脚踏实地的做科研，还有大胆创新的精神让我们学习科研的探索精神。

感谢天津医科大学第二医院泌尿外科全体医护人员对于我临床实习期间给予的帮助。感谢权昌益主任、马宝杰主任、李刚主任、王勇主任等在我临床实践过程中的细致的指导。各位老师精湛的诊疗技术令人惊叹值得我们学习，而关怀患者，设身处地为患者着想的崇高医德更是值得我毕生学习。

感谢天津市泌尿外科研究所性激素国际合作实验室老师同学的帮助。感谢各位师兄在实验上对我的指导与帮助，感谢各位师弟妹的热心帮助。

感谢我的父母家人，对我的支持。

最后感谢参加论文评审及答辩的各位老师，对我三年研究生学习成果的审查，及对我的研究方向的指导。

诚挚的感谢每一位帮助过我的人！

个人简历

姓名：智超 **性别：**男

出生年月：1993 年 1 月 **籍贯：**江苏兴化

主要学习和工作经历：（从本科开始）

2012 年 9 月-2017 年 7 月 中国医科大学 临床医学专业，学士学位

2017 年 9 月-至今 天津医科大学 研究生学院 硕士在读

在学期间发表论文情况：

[1]Li G, Zhi C, Zhu D,et al. Efficacy of povidone-iodine against accidental tumor incision during nephron-sparing surgery: experimental study in patients with renal cell carcinoma[J].J Int Med Res, 2019, 47(10):4993-5002. SCI 收录 IF.1.351
