# Supplementary material for: Post-transcriptional modification of m6A methylase METTL3 regulates ERK-induced androgen-deprived treatment resistance prostate cancer
Source: Cell Death Dis. 2023 Apr 24;14(4):289. doi: 10.1038/s41419-023-05773-5 (PMC10126012; doi:10.1038/s41419-023-05773-5)
Supplement: Supplementary file 7 — Original Data File [file 41419_2023_5773_MOESM7_ESM.ppt]

## Slide 1
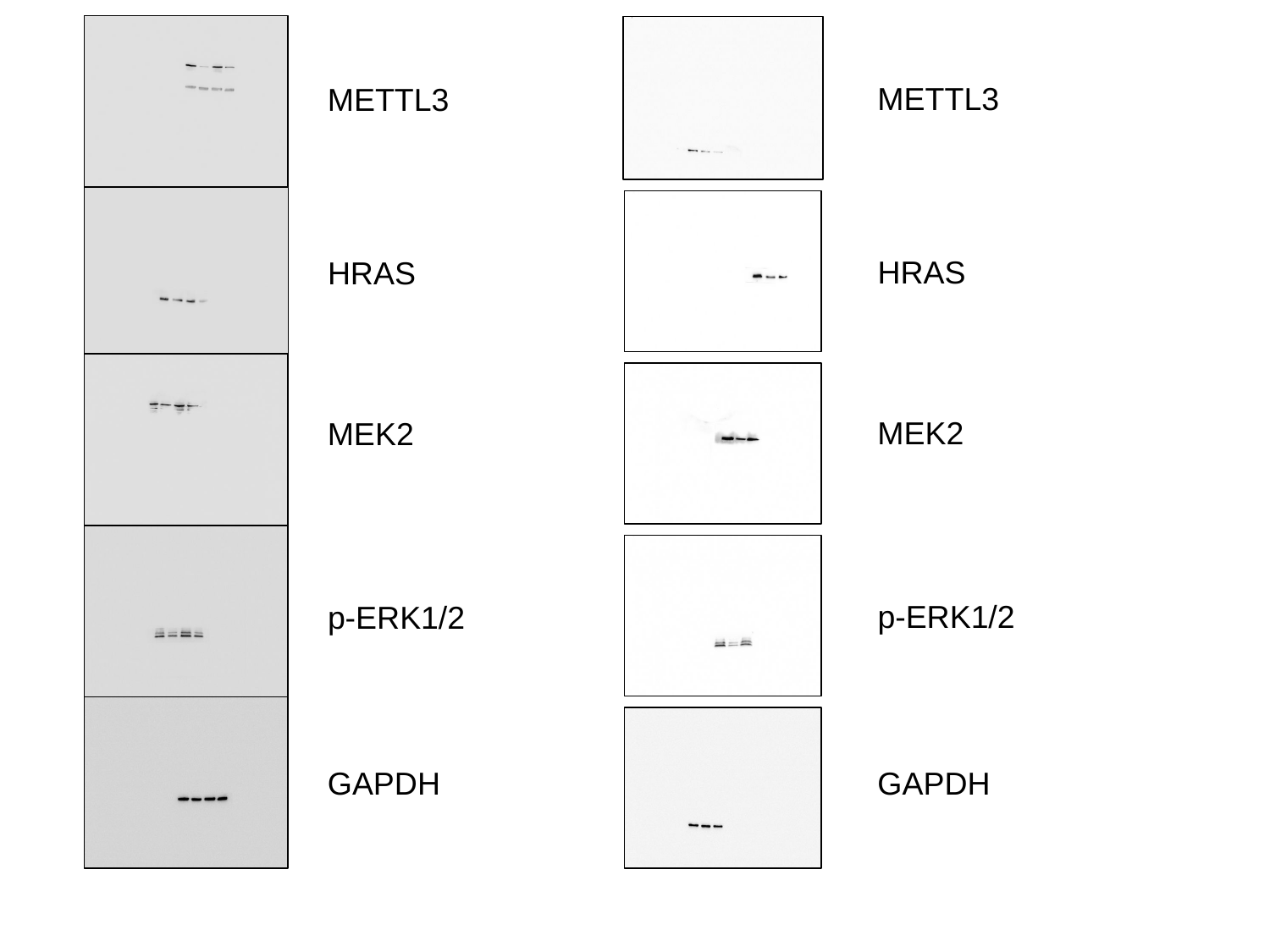

METTL3
METTL3
HRAS
HRAS
MEK2
MEK2
p-ERK1/2
p-ERK1/2
GAPDH
GAPDH

## Slide 2
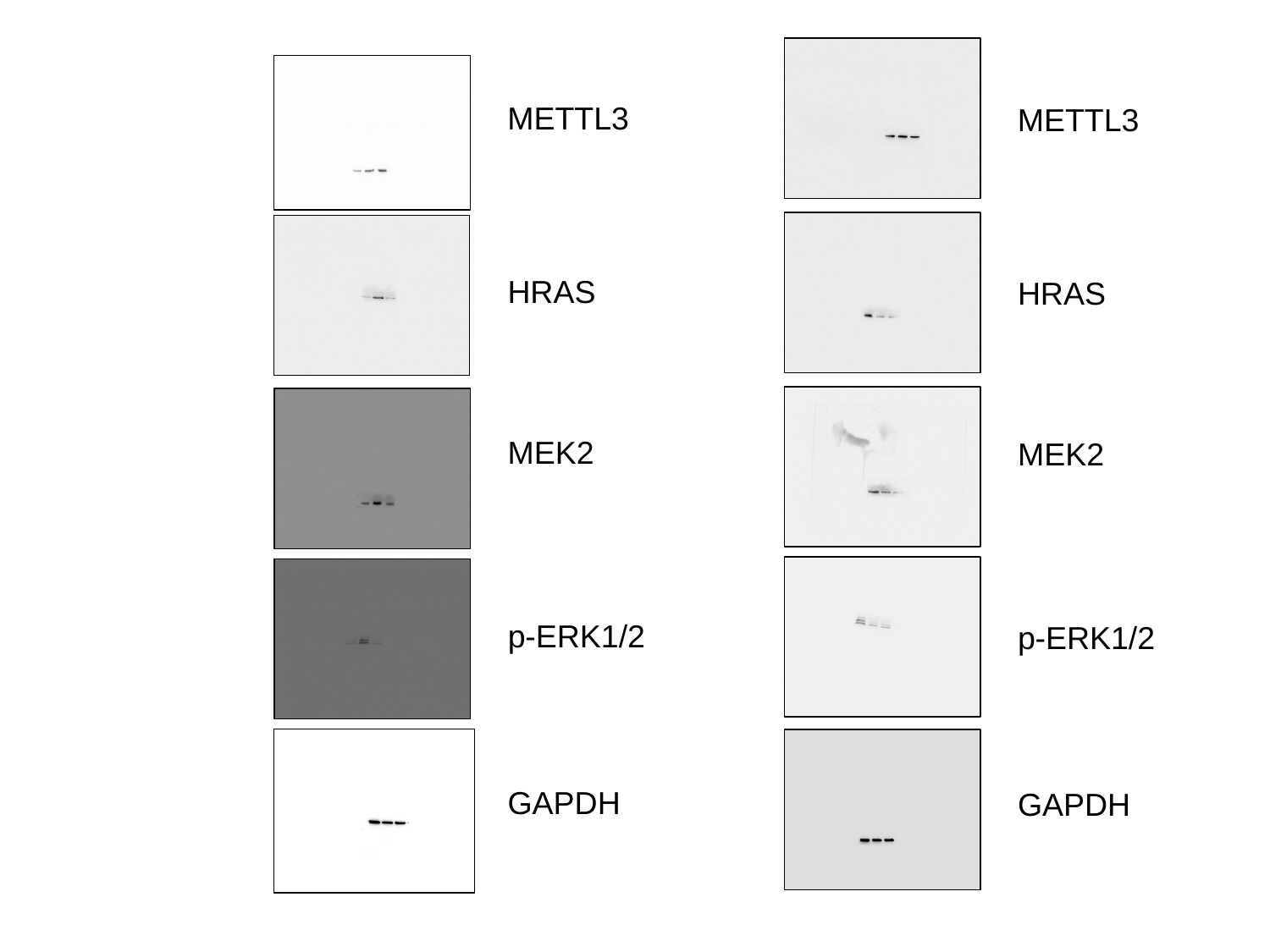

METTL3
METTL3
HRAS
HRAS
MEK2
MEK2
p-ERK1/2
p-ERK1/2
GAPDH
GAPDH

## Slide 3
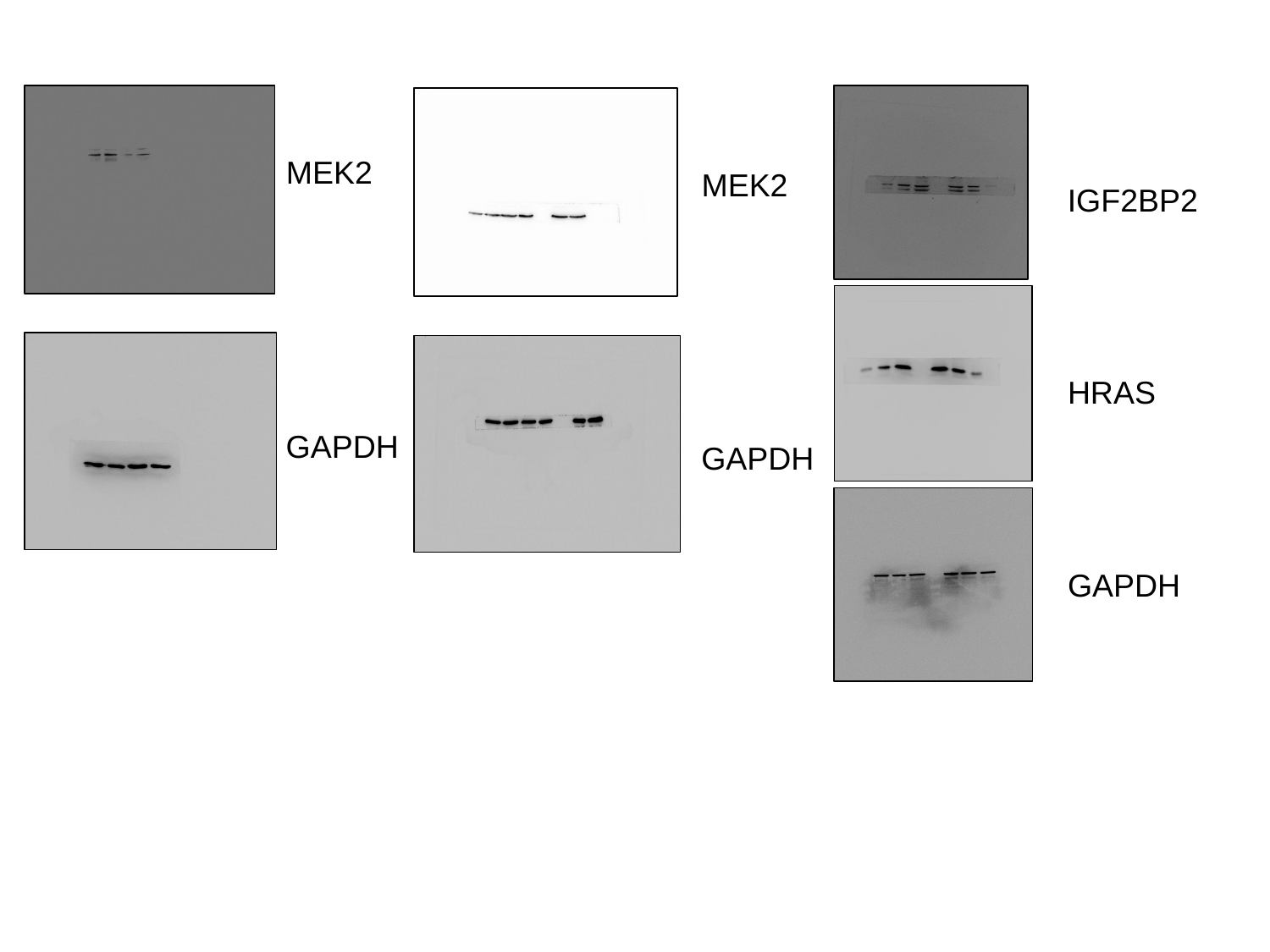

MEK2
MEK2
IGF2BP2
HRAS
GAPDH
GAPDH
GAPDH

## Slide 4
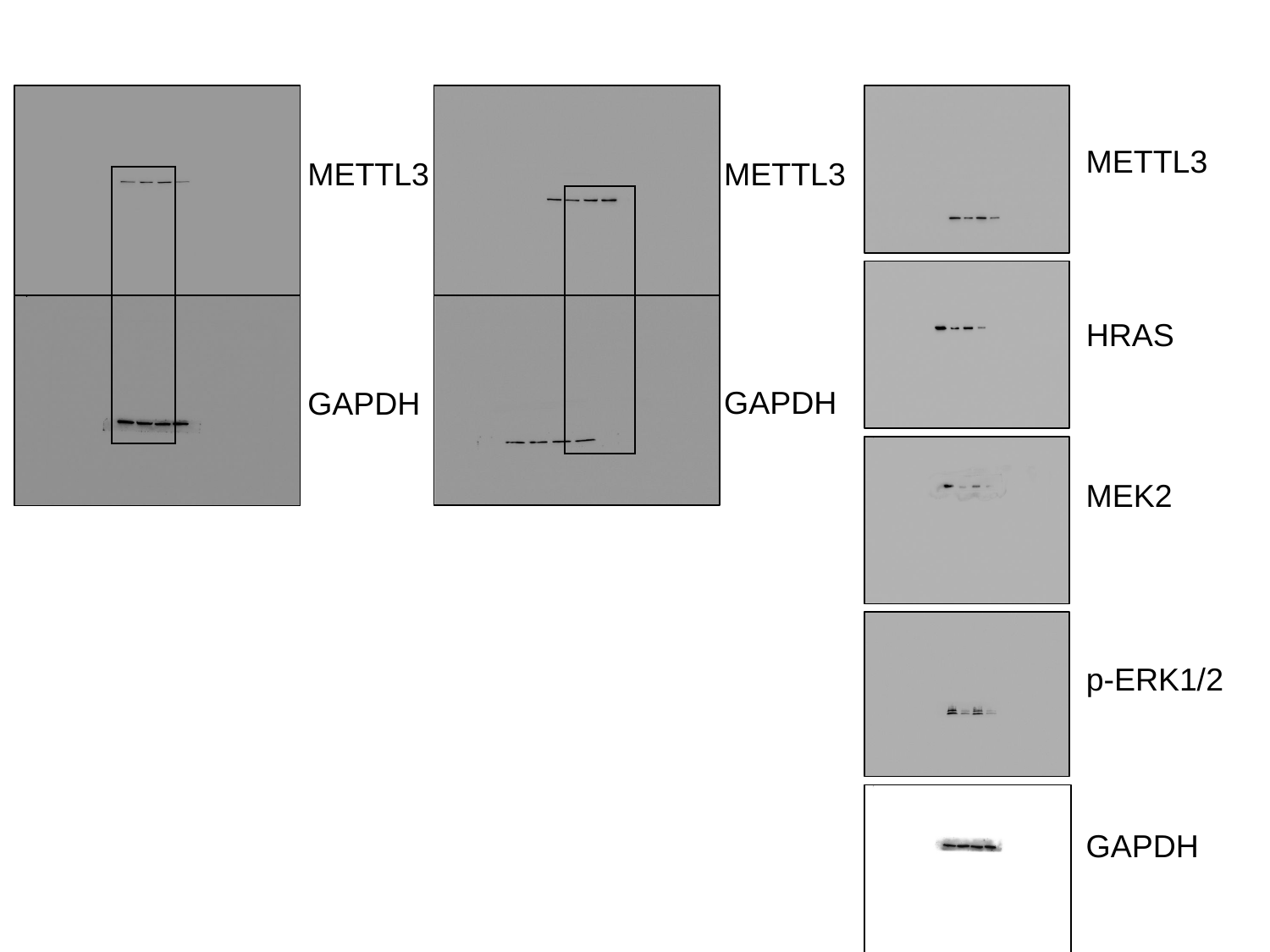

METTL3
METTL3
METTL3
HRAS
GAPDH
GAPDH
MEK2
p-ERK1/2
GAPDH

## Slide 5
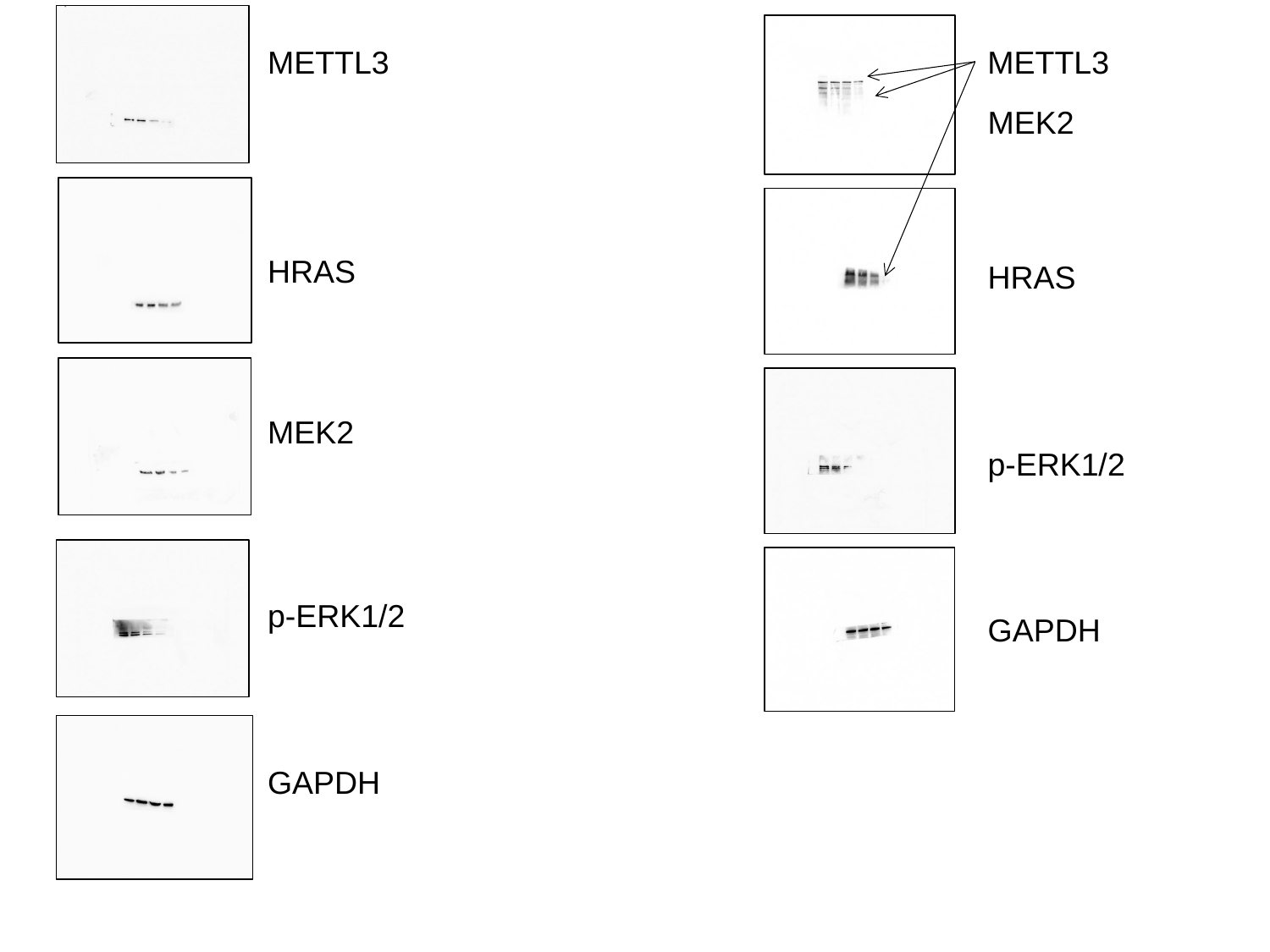

METTL3
METTL3
MEK2
HRAS
HRAS
MEK2
p-ERK1/2
p-ERK1/2
GAPDH
GAPDH
